# Urogenital congenital anomalies in children under 9 years: global disease burden analysis and projections, 1990–2021

**DOI:** 10.1016/j.jped.2026.101576

**Published:** 2026-06-15

**Authors:** Sheng Gong, Jianming Zhu, Guoping Jiang, Weiwei Ruan

**Affiliations:** Department of Pediatric Surgery, The Affiliated Women and Children’s Hospital of Ningbo University, Zhejiang, PR China

**Keywords:** Urogenital congenital anomalies, Deaths, DALYs, Age-standardized rates, SDI, Projections

## Abstract

**Objective:**

Urogenital congenital anomalies (UGCAs), including congenital anomalies of the kidney and urinary tract and external genital malformations, are major causes of early-life morbidity, preventable mortality, and long-term disability, particularly in low-resource settings. This study assessed the global burden, temporal trends, regional disparities, and future projections of UGCAs in children under 9 years using the Global Burden of Disease (GBD) 2021 dataset.

**Methods:**

GBD 2021 data were used to estimate incidence, prevalence, mortality, and disability-adjusted life years (DALYs) in children aged 0–8 years. Analyses were stratified by sex, age, Socio-demographic Index (SDI), region, and country. Age-standardized rates (ASRs), SDI associations, and decomposition analyses were conducted, and future trends were projected using ARIMA and Holt–Winters models. Estimates are presented with 95% uncertainty intervals (UIs).

**Results:**

From 1990 to 2021, global age-standardized incidence (ASIR) and prevalence rates (ASPR) remained stable, whereas mortality and DALY rates declined. In 2021, incidence was 2026,032 (95% UI: 1417,198–2787,372), prevalence 3360,751 (2594,010–4411,084), DALYs 5122,959 (4069,356–6172,763), and deaths 58,906 (46,861–70,940). ASRs were 150.46 per 100,000 for incidence, 248.91 for prevalence, 384.10 for DALYs, and 4.41 for deaths. Females had higher ASIRs and ASPRs, while males showed higher mortality and DALY rates. Incidence and prevalence increased with age, whereas mortality declined. With increasing SDI, incidence and prevalence rose, while mortality decreased. The burden is increasingly concentrated in low-SDI regions; Southern Sub-Saharan Africa was the only region with rising mortality. CAKUT remained the predominant subtype globally. Projections indicate continued declines in mortality, with stable incidence and prevalence.

**Conclusion:**

Although global mortality and DALYs from UGCAs have declined, the growing burden in low-SDI regions highlights persistent inequalities and the need for targeted interventions.

## Introduction

Urogenital congenital anomalies (UGCAs), including congenital anomalies of the kidney and urinary tract (CAKUT) and malformations of the external genitalia, are major contributors to neonatal and early-childhood morbidity and mortality worldwide, second only to preventable perinatal conditions in many settings [1]. Global estimates indicate that hundreds of thousands of children are affected annually, with many experiencing long-term renal and reproductive complications if not diagnosed and treated in a timely manner [2,3]. Despite their clinical significance, UGCAs remain under-recognized, particularly in low- and middle-income countries where birth-defect surveillance systems are limited [4,5]. Compared with advances in adult urology and nephrology, progress in pediatric UGCA management has been uneven. Care often depends on limited surgical and supportive interventions, with restricted access to prenatal screening, neonatal surgery, interventional nephrology, and long-term multidisciplinary follow-up. As a result, many affected children develop chronic complications, including chronic kidney disease, hypertension, recurrent urinary tract infections, subfertility or infertility, and psychosocial challenges [6,7]. The burden also extends to families, who frequently experience psychological stress and financial strain related to prolonged care needs.

In addition, marked geographic disparities persist in outcomes. Mortality and disability attributable to congenital anomalies remain disproportionately high in low-resource settings, whereas high-income regions benefit from early detection, timely referral, and standardized care pathways [8,9]. These inequalities highlight ongoing gaps in prevention and treatment and suggest uneven progress toward global targets for reducing preventable deaths and disability from birth defects by 2030. Although studies using the Global Burden of Disease (GBD) framework have expanded, most have focused on specific anomalies or regions rather than the full spectrum of UGCAs [10,11]. Importantly, most cases are identified in early childhood, with the majority diagnosed before 9 years of age. In this study, the authors used data from GBD 2021 to provide a comprehensive assessment of the global burden of UGCAs in children under 9 years. The authors evaluated temporal trends, regional disparities, and future projections to inform prevention strategies, optimize resource allocation, and support improvements in child health.

## Methods

### Data sources and study design

The authors used data from the Global Burden of Disease (GBD) 2021 study to assess the burden of UGCAs among children aged 0–9 years at global, regional, and national levels from 1990 to 2021, with projections to 2036. Data were obtained from the Global Health Data Exchange (GHDx), which compiles standardized estimates for 371 diseases across 204 countries and territories [12]. UGCAs were defined according to GBD cause classifications and corresponding ICD codes (ICD-10: Q50–Q56, Q60–Q64; ICD-9: 752.x, 753.x). Estimates were derived from multiple sources, including vital registration, hospital records, registries, and surveys, with adjustments for underreporting and misclassification.

The authors extracted incidence, prevalence, mortality, and disability-adjusted life years (DALYs), where DALYs comprise years of life lost (YLLs) and years lived with disability (YLDs). Socioeconomic development was measured using the Socio-demographic Index (SDI), a composite indicator of income, education, and fertility, categorized into five quintiles. This study followed GATHER guidelines; ethical approval was not required as all data were de-identified and publicly available.

### Burden estimation and stratification

The authors calculated age-standardized incidence (ASIR), prevalence (ASPR), mortality (ASMR), and DALY rates (ASDR), each with 95% uncertainty intervals (UIs), across countries, 21 GBD regions, and SDI quintiles. Analyses were stratified by sex and age groups (< 1, 1–4, and 5–9 years). For age-specific aggregation, estimates were derived by weighting each age group according to its population proportion rather than by simple summation. Specifically, for a given metric, the combined estimate was calculated as:

$$R=\sum_{i}w_{i}\times r_{i}$$

where $r_{i}$ denotes the age-specific rate for group i and $w_{i}$ represents the corresponding population weight. Absolute counts (incidence, prevalence, deaths, and DALYs) were also summarized, with DALYs further decomposed into years of life lost (YLLs) and years lived with disability (YLDs). Geographic patterns and temporal trends were visualized using standardized mapping approaches.

### SDI correlation analysis

Associations between SDI and UGCA burden indicators (counts and age-standardized rates) were assessed using Pearson correlation at global and national levels, with Spearman correlation used in sensitivity analyses to account for nonlinearity and outliers [13].

### Decomposition analysis

Changes in absolute burden from 1990 to 2021 were decomposed into three components: population growth, age structure shifts, and epidemiological changes (age-specific rates) [14]. A stepwise replacement (Das Gupta) method was applied, consistent with GBD practice. Uncertainty was propagated using 1000 posterior draws, and results are presented as medians with 95% UIs. Sensitivity analyses assessed robustness to component ordering.

### Health inequality analysis

Inequalities in ASMR and ASDR were quantified using the Slope Index of Inequality (SII) and Concentration Index (CI) [15]. SII was estimated via population-weighted regression across SDI ranks, representing absolute differences, while CI captured relative inequality (range −1 to +1). Analyses were conducted globally, by region, and by country for 1990 and 2021, with 95% confidence intervals obtained via bootstrap resampling.

### Forecasting analysis

Future trends were projected using AutoRegressive Integrated Moving Average (ARIMA) models, with Holt–Winters exponential smoothing applied as a complementary approach to assess robustness [16,17]. Both methods were selected due to their suitability for epidemiological time-series data without requiring strong parametric assumptions. Models were fitted to annual time series (1990–2021) for incidence, prevalence, mortality, and DALYs at global, regional, SDI, and national levels, where data were sufficiently complete. For ARIMA models, orders (p, d, q) were determined using a combination of autocorrelation function (ACF), partial autocorrelation function (PACF), and minimization of Akaike and Bayesian Information Criteria (AIC/BIC). Holt–Winters models were specified with additive or multiplicative components based on data structure and seasonal diagnostics. Model adequacy was evaluated using residual diagnostics, including Ljung–Box tests for independence and assessment of stationarity and residual distribution. Forecasts are presented with 95% prediction intervals.

### Statistical software

All analyses were conducted using R (version 4.3.3) and Stata 18. Mapping and visualization were performed using ArcGIS, QGIS, and R packages. Statistical significance was defined as *p* < 0.05. GBD estimates are presented with 95% UIs, and forecasts with 95% prediction intervals.

## Results

### Global burden of UGCAs in children under 9 years (1990–2021)

**Figure 1:**
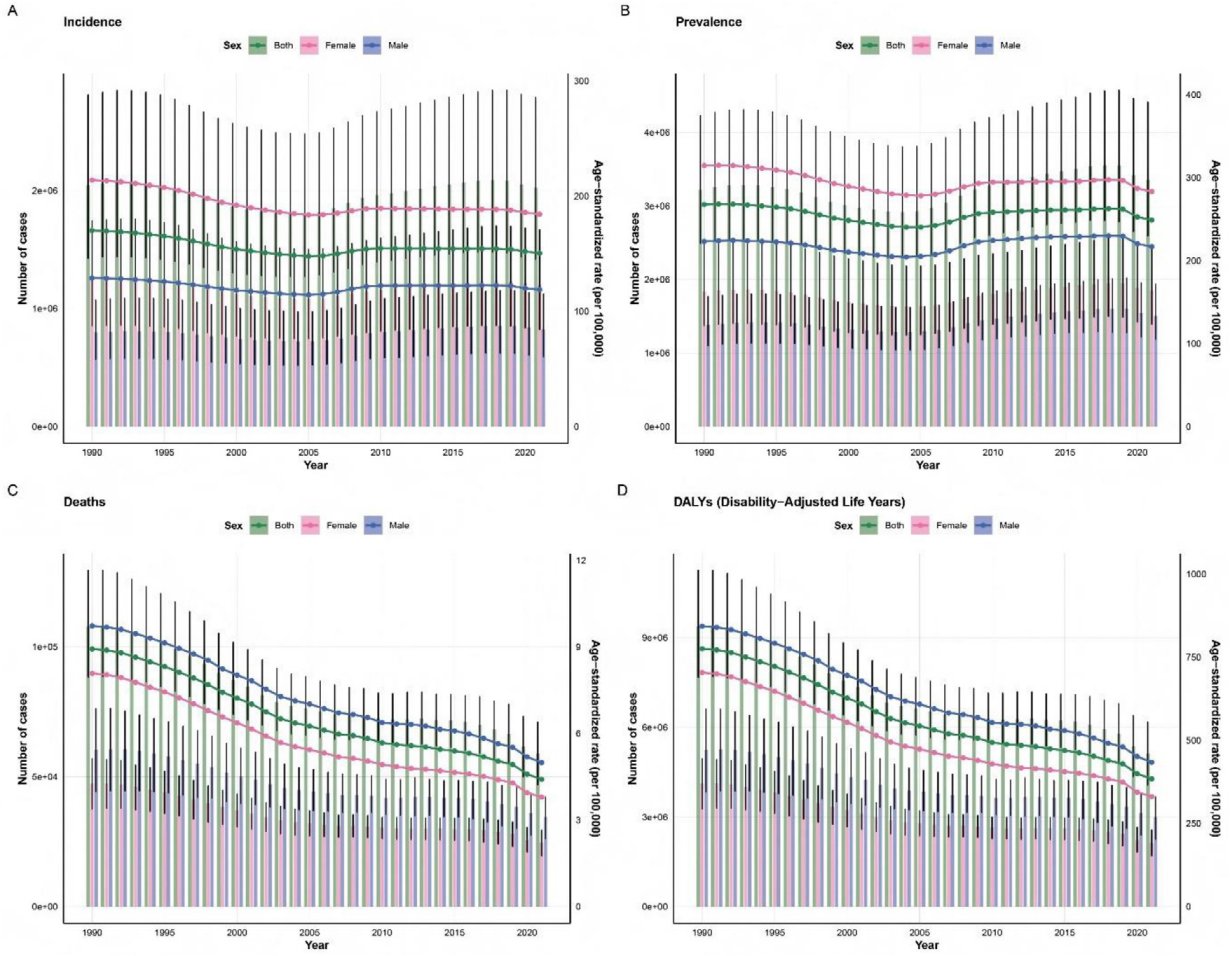
Trends in the absolute numbers and corresponding Age-Standardized Rates (ASRs) of the global disease burden for urogenital congenital anomalies (UGCAs) in children under 9 years old from 1990 to 2021 (A. Incidence; B. Prevalence; C. Deaths; D. DALYs).

From 1990 to 2021, global incidence and prevalence of UGCAs remained relatively stable, with minor fluctuations. In 2021, there were 2026,032 incident cases (95% UI: 1417,198–2787,372) and 3360,751 prevalent cases (95% UI: 2594,010–4411,084), corresponding to ASIR and ASPR of 150.46 and 248.91 per 100,000, respectively. Compared with 1990, incidence and prevalence increased slightly by 0.99% and 4.49%, with EAPCs of −0.31 and −0.06. In contrast, mortality and DALYs declined markedly. In 2021, DALYs totaled 5122,959 (95% UI: 4069,356–6172,763) and deaths 58,906 (95% UI: 46,861–70,940), with ASDR and ASMR of 384.10 and 4.41 per 100,000, respectively. Relative to 1990, DALYs and deaths decreased by 45.28% and 45.32%, with EAPCs of −2.16 and −2.18 (Figure 1 and Tables 1, 2, Table 3, Table 4).

**Table 1:** The incidence cases and age-standardized incidence rate of Urogenital Congenital Anomalies in Children Under 9 Years in 1990 and 2021, along with their temporal trend.

	Rate per 100 000(95%UI)		2021		1990–2021
	1990				
	Incidence cases	The age-standardized incidence rate	Incidence cases	The age-standardized incidence rate	EAPC
**Global**	2046,249 (1426,705 to 2811,373)	170.1 (118.48 to 233.9)	2026,032 (1417,198 to 2787,372)	150.46 (105.65 to 206.32)	−0.31 (−0.42 to −0.19)
**SDI region**					
High SDI	328,838 (229,151 to 453,634)	264.33 (184.63 to 363.9)	291,375 (208,892 to 396,274)	259.3 (186.78 to 351)	0.06 (−0.23 to 0.35)
High-middle SDI	465,341 (322,229 to 641,735)	252.91 (175.29 to 348.51)	340,168 (239,237 to 466,048)	223.28 (158.47 to 303.48)	−0.37 (−0.44 to −0.31)
Middle SDI	641,290 (447,517 to 882,132)	162.74 (113.59 to 223.83)	581,805 (404,969 to 804,552)	155.57 (109.01 to 213.91)	−0.13 (−0.19 to −0.08)
Low-middle SDI	463,021 (323,313 to 642,132)	138.94 (96.84 to 193.02)	548,809 (377,268 to 767,407)	141.88 (97.79 to 197.93)	0.26 (0.14 to 0.37)
Low SDI	145,030 (103,282 to 197,017)	87.29 (61.77 to 119.31)	261,834 (185,160 to 362,718)	82.13 (57.97 to 113.95)	−0.02 (−0.11 to 0.08)
**GBD region**					
Andean Latin America	9890 (7401 to 13,067)	96.62 (72.24 to 127.8)	11,375 (8570 to 14,970)	92.92 (70.08 to 122.16)	−0.03 (−0.06 to −0.01)
Australasia	2645 (1911 to 3578)	86.22 (62.37 to 116.54)	3091 (2229 to 4187)	82.28 (59.56 to 110.98)	−0.01 (−0.05 to 0.03)
Caribbean	7687 (5548 to 10,267)	97.84 (70.44 to 131)	7388 (5268 to 9923)	95.69 (68.31 to 128.36)	−0.03 (−0.04 to −0.02)
Central Asia	68,802 (48,105 to 94,688)	386.77 (269.31 to 534.38)	75,634 (52,655 to 104,543)	389.24 (270.77 to 538.4)	0.03 (0.03 to 0.04)
Central Europe	118,979 (82,905 to 163,472)	628.94 (440.2 to 860.47)	76,022 (56,903 to 99,481)	669.39 (502.19 to 873.84)	0.28 (−0.06 to 0.64)
Central Latin America	56,975 (40,972 to 77,304)	128.86 (92.52 to 175.12)	50,047 (35,682 to 68,822)	120.36 (86.12 to 164.84)	−0.22 (−0.25 to −0.19)
Central Sub-Saharan Africa	9988 (6889 to 13,940)	54.11 (36.98 to 76.2)	20,883 (14,008 to 29,734)	50.99 (34.17 to 72.65)	−0.17 (−0.18 to −0.16)
East Asia	445,495 (311,568 to 614,107)	199.18 (139.06 to 274.95)	303,288 (213,861 to 415,937)	170.11 (121.43 to 230.98)	−0.65 (−0.74 to −0.55)
Eastern Europe	159,804 (108,724 to 221,515)	456.6 (311.6 to 631.4)	103,270 (68,797 to 144,978)	447.91 (302.21 to 622.63)	−0.07 (−0.09 to −0.05)
Eastern Sub-Saharan Africa	34,444 (25,514 to 45,198)	52.19 (38.4 to 68.93)	58,068 (42,618 to 79,026)	47.09 (34.52 to 64.16)	−0.28 (−0.33 to −0.24)
High-income Asia Pacific	91,891 (62,620 to 130,295)	414.4 (284.89 to 582.76)	56,576 (39,727 to 78,800)	397.23 (282.06 to 547.76)	−0.21 (−0.24 to −0.17)
High-income North America	91,186 (63,375 to 126,121)	216.18 (150.13 to 299.23)	115,260 (81,137 to 159,568)	271.88 (192.11 to 374.96)	0.85 (0.28 to 1.42)
North Africa and Middle East	88,349 (62,546 to 120,826)	89.65 (63.37 to 122.79)	106,500 (74,032 to 148,741)	85.33 (59.51 to 118.84)	−0.11 (−0.13 to −0.1)
Oceania	2503 (1695 to 3534)	132.77 (89.56 to 188.17)	4797 (3259 to 6743)	133.53 (90.26 to 188.6)	0.02 (0.01 to 0.02)
South Asia	490,215 (338,255 to 681,970)	159.96 (110.32 to 222.62)	635,452 (429,260 to 892,309)	192.9 (131.04 to 269.75)	0.91 (0.73 to 1.09)
Southeast Asia	183,441 (124,998 to 257,161)	157.95 (107.76 to 221.13)	181,460 (127,248 to 251,748)	158.5 (111.47 to 219.24)	0.08 (0.05 to 0.11)
Southern Latin America	17,205 (11,844 to 23,981)	169.5 (116.73 to 236.17)	15,721 (11,587 to 21,138)	165.39 (122.97 to 220.51)	−0.56 (−0.71 to −0.4)
Southern Sub-Saharan Africa	19,810 (13,355 to 28,026)	138.34 (93.08 to 196.04)	22,581 (15,266 to 32,128)	138.99 (94.2 to 197.33)	0.03 (0.02 to 0.04)
Tropical Latin America	31,412 (23,387 to 41,045)	88.13 (65.88 to 114.7)	20,071 (14,749 to 26,818)	59.18 (43.5 to 79.05)	−1.32 (−1.45 to −1.18)
Western Europe	76,924 (55,645 to 103,482)	165.8 (120.22 to 222.54)	70,241 (51,712 to 93,036)	159.45 (117.84 to 210.33)	0 (−0.07 to 0.06)
Western Sub-Saharan Africa	38,605 (27,898 to 52,128)	60.07 (42.95 to 81.87)	88,308 (61,922 to 121,397)	58.36 (40.78 to 80.47)	−0.05 (−0.07 to −0.02)

EAPC, estimated annual percentage change; SDl, Sociodemographic Index; Ul, uncertainty interval. EAPC is expressed as 95% CIs.

**Table 2:** The prevalence cases and age-standardized prevalence rate of Urogenital Congenital Anomalies in Children Under 9 Years in 1990 and 2021, along with their temporal trend.

	Rate per 100 000(95%UI)		2021		1990–2021
	1990				
	Prevalence cases	The age-standardized prevalence rate	Prevalence cases	The age-standardized prevalence rate	EAPC
**Global**	3216,298 (2490,365 to 4228,140)	267.51 (206.98 to 351.99)	3360,751 (2594,010 to 4411,084)	248.91 (192.56 to 325.95)	−0.06 (−0.19 to 0.07)
**SDI region**					
High SDI	543,745 (425,703 to 708,729)	436.13 (341.97 to 567.32)	487,194 (391,921 to 609,915)	430.3 (346.98 to 537)	0.13 (−0.08 to 0.35)
High-middle SDI	687,777 (528,626 to 899,452)	373.68 (287.42 to 488.33)	560,377 (431,868 to 730,774)	367.76 (284.36 to 477.77)	0.11 (0.02 to 0.2)
Middle SDI	993,879 (764,059 to 1309,588)	252.2 (193.9 to 332.27)	971,055 (746,925 to 1279,554)	258.31 (199.39 to 339.11)	0.21 (0.11 to 0.3)
Low-middle SDI	721,267 (538,939 to 966,059)	216.84 (161.84 to 290.83)	878,756 (657,012 to 1184,151)	226.49 (169.66 to 304.67)	0.39 (0.26 to 0.53)
Low SDI	265,963 (203,144 to 343,198)	159.41 (121.35 to 206.68)	460,575 (346,071 to 609,246)	144.45 (108.44 to 191.27)	−0.1 (−0.22 to 0.03)
**GBD region**					
Andean Latin America	16,910 (13,831 to 20,735)	165.34 (135.17 to 202.87)	20,763 (16,589 to 25,602)	169.42 (135.34 to 208.89)	0.33 (0.26 to 0.4)
Australasia	5505 (4633 to 6607)	179.59 (151.21 to 215.37)	6493 (5346 to 7832)	173.27 (142.79 to 208.73)	0.26 (0.06 to 0.46)
Caribbean	13,948 (11,065 to 17,367)	177.81 (140.95 to 221.81)	13,286 (10,419 to 16,865)	171.8 (134.76 to 217.92)	0 (−0.03 to 0.03)
Central Asia	84,221 (61,647 to 112,937)	476.39 (347.77 to 640.61)	92,754 (67,345 to 125,347)	477.97 (346.9 to 646.2)	0.03 (0 to 0.05)
Central Europe	146,198 (106,676 to 196,901)	766.69 (560.19 to 1029.57)	87,551 (68,178 to 110,588)	766.55 (597.51 to 966.93)	0 (−0.3 to 0.3)
Central Latin America	96,694 (76,837 to 123,094)	219.38 (174.16 to 279.57)	89,580 (71,301 to 113,999)	213.67 (170.31 to 271.39)	0.04 (−0.03 to 0.1)
Central Sub-Saharan Africa	16,636 (11,683 to 22,247)	89.49 (62.75 to 120.33)	30,194 (22,242 to 41,064)	73.75 (54.29 to 100.36)	−0.56 (−0.61 to −0.52)
East Asia	713,118 (539,255 to 941,395)	318.74 (240.73 to 421.42)	586,942 (441,649 to 772,428)	331.9 (249.5 to 436.31)	0.28 (0.17 to 0.38)
Eastern Europe	210,409 (156,238 to 280,928)	599.6 (446.08 to 799.22)	131,433 (94,820 to 179,605)	563.49 (409.97 to 764.48)	−0.23 (−0.31 to −0.14)
Eastern Sub-Saharan Africa	85,279 (65,920 to 106,357)	127.04 (98.1 to 158.82)	128,534 (96,470 to 170,359)	103.98 (78.08 to 137.79)	−0.5 (−0.62 to −0.38)
High-income Asia Pacific	142,931 (108,039 to 191,133)	636.48 (483.58 to 846.04)	92,195 (72,039 to 118,263)	636.23 (499.73 to 810.96)	−0.01 (−0.05 to 0.03)
High-income North America	179,795 (142,920 to 230,717)	426.52 (338.86 to 547.67)	206,421 (162,154 to 262,145)	483.19 (380.41 to 612.2)	0.6 (0.17 to 1.03)
North Africa and Middle East	157,805 (121,346 to 205,309)	160.13 (123.04 to 208.6)	198,136 (154,441 to 257,092)	158.74 (123.99 to 205.51)	0.16 (0.09 to 0.24)
Oceania	3355 (2384 to 4692)	178.97 (126.81 to 251.06)	6558 (4716 to 9060)	183.68 (131.58 to 254.8)	0.07 (0.04 to 0.09)
South Asia	762,723 (562,004 to 1038,091)	249.03 (183.44 to 339.05)	1020,215 (749,289 to 1399,971)	307.26 (226.44 to 420.34)	1.04 (0.83 to 1.25)
Southeast Asia	256,259 (185,841 to 351,196)	220.32 (159.89 to 301.62)	256,609 (193,723 to 340,099)	223.17 (168.74 to 295.17)	0.1 (0.07 to 0.13)
Southern Latin America	25,308 (19,095 to 34,003)	249.19 (188.07 to 334.71)	24,465 (19,759 to 30,651)	254.45 (206.27 to 317.33)	−0.36 (−0.51 to −0.2)
Southern Sub-Saharan Africa	31,815 (22,820 to 44,170)	222.85 (159.63 to 309.79)	37,571 (27,386 to 51,700)	230.41 (168.25 to 316.55)	0.15 (0.11 to 0.2)
Tropical Latin America	49,276 (40,799 to 59,756)	137.87 (114.37 to 166.72)	34,633 (27,983 to 43,158)	102.08 (82.49 to 127.19)	−0.78 (−0.95 to −0.61)
Western Europe	141,276 (118,207 to 170,733)	304.54 (255.2 to 367.34)	129,329 (109,239 to 154,347)	292.89 (247.68 to 348.88)	−0.01 (−0.13 to 0.11)
Western Sub-Saharan Africa	76,838 (60,315 to 96,331)	117.89 (92.03 to 148.91)	167,089 (119,163 to 222,936)	110 (78.48 to 146.9)	−0.11 (−0.19 to −0.03)

EAPC, estimated annual percentage change; SDl, Sociodemographic Index; Ul, uncertainty interval. EAPC is expressed as 95% CIs.

**Table 3:** The Deaths cases and age-standardized Deaths rate of Urogenital Congenital Anomalies in Children Under 9 Years in 1990 and 2021, along with their temporal trend.

	Rate per 100 000(95%UI)		2021		1990–2021
	1990				
	Deaths cases	The age-standardized deaths rate	Deaths cases	The age-standardized deaths rate	EAPC
**Global**	107,732 (88,388 to 129,395)	8.93 (7.32 to 10.72)	58,906 (46,861 to 70,940)	4.41 (3.5 to 5.32)	−2.18 (−2.28 to −2.07)
**SDI region**					
High SDI	5194 (4985 to 5417)	4.18 (4.01 to 4.36)	2300 (2125 to 2479)	2.04 (1.88 to 2.2)	−2.04 (−2.15 to −1.93)
High-middle SDI	20,170 (16,807 to 23,574)	10.99 (9.16 to 12.85)	4898 (3967 to 5799)	3.22 (2.6 to 3.83)	−4.09 (−4.28 to −3.9)
Middle SDI	40,596 (32,787 to 48,560)	10.31 (8.32 to 12.33)	14,199 (11,349 to 17,253)	3.8 (3.03 to 4.64)	−3.12 (−3.26 to −2.98)
Low-middle SDI	24,754 (17,771 to 32,334)	7.39 (5.31 to 9.64)	17,514 (14,183 to 21,054)	4.55 (3.68 to 5.48)	−1.32 (−1.4 to −1.24)
Low SDI	16,943 (11,674 to 22,678)	9.89 (6.81 to 13.24)	19,945 (14,022 to 25,685)	6.21 (4.37 to 7.99)	−1.35 (−1.46 to −1.24)
**GBD region**					
Andean Latin America	1075 (874 to 1359)	10.48 (8.53 to 13.24)	682 (508 to 882)	5.56 (4.14 to 7.2)	−1.73 (−1.88 to −1.59)
Australasia	117 (109 to 125)	3.8 (3.56 to 4.07)	72 (62 to 83)	1.9 (1.64 to 2.19)	−1.87 (−2.01 to −1.74)
Caribbean	781 (536 to 1040)	9.87 (6.8 to 13.12)	568 (372 to 790)	7.36 (4.81 to 10.27)	−0.62 (−0.76 to −0.48)
Central Asia	1542 (1356 to 1766)	8.62 (7.59 to 9.84)	1075 (884 to 1313)	5.53 (4.55 to 6.75)	−1.27 (−1.36 to −1.18)
Central Europe	1265 (1176 to 1360)	6.7 (6.22 to 7.21)	263 (230 to 302)	2.3 (2.01 to 2.65)	−3.3 (−3.45 to −3.15)
Central Latin America	3859 (3579 to 4204)	8.71 (8.08 to 9.48)	1719 (1394 to 2158)	4.13 (3.33 to 5.2)	−1.8 (−2.02 to −1.58)
Central Sub-Saharan Africa	1016 (567 to 1460)	5.24 (2.96 to 7.51)	1039 (751 to 1421)	2.53 (1.83 to 3.46)	−1.99 (−2.18 to −1.81)
East Asia	36,482 (27,999 to 45,102)	16.2 (12.44 to 20.02)	7001 (5282 to 9006)	3.93 (2.94 to 5.09)	−4.72 (−4.97 to −4.47)
Eastern Europe	2908 (2745 to 3077)	8.32 (7.85 to 8.81)	652 (587 to 722)	2.88 (2.57 to 3.2)	−3.57 (−3.8 to −3.34)
Eastern Sub-Saharan Africa	8904 (6263 to 11,758)	13.07 (9.19 to 17.27)	9837 (6821 to 13,260)	7.94 (5.51 to 10.69)	−1.37 (−1.51 to −1.23)
High-income Asia Pacific	949 (850 to 1060)	4.26 (3.82 to 4.76)	272 (240 to 298)	1.91 (1.68 to 2.1)	−2.49 (−2.67 to −2.32)
High-income North America	1520 (1487 to 1554)	3.6 (3.53 to 3.69)	874 (799 to 953)	2.06 (1.88 to 2.25)	−1.43 (−1.54 to −1.32)
North Africa and Middle East	8026 (5937 to 10,385)	8.11 (6.01 to 10.49)	5104 (3978 to 6171)	4.11 (3.2 to 4.97)	−1.82 (−2.01 to −1.64)
Oceania	66 (41 to 100)	3.5 (2.16 to 5.23)	125 (81 to 193)	3.44 (2.22 to 5.3)	−0.02 (−0.24 to 0.2)
South Asia	20,564 (13,992 to 27,944)	6.7 (4.56 to 9.11)	13,070 (10,514 to 16,216)	4.02 (3.23 to 4.99)	−1.53 (−1.64 to −1.42)
Southeast Asia	7629 (4857 to 10,818)	6.59 (4.18 to 9.35)	4571 (3565 to 5679)	4 (3.11 to 4.98)	−1.5 (−1.54 to −1.45)
Southern Latin America	539 (501 to 578)	5.31 (4.93 to 5.7)	281 (240 to 331)	2.95 (2.5 to 3.48)	−1.42 (−1.62 to −1.22)
Southern Sub-Saharan Africa	497 (382 to 618)	3.45 (2.66 to 4.28)	597 (455 to 761)	3.69 (2.81 to 4.71)	0.62 (0.3 to 0.95)
Tropical Latin America	2550 (2213 to 2892)	7.22 (6.26 to 8.2)	1209 (924 to 1470)	3.56 (2.72 to 4.33)	−1.55 (−1.95 to −1.14)
Western Europe	2038 (1980 to 2095)	4.38 (4.26 to 4.51)	965 (884 to 1050)	2.18 (1.99 to 2.37)	−2.12 (−2.23 to −2.01)
Western Sub-Saharan Africa	5405 (4199 to 6626)	8.02 (6.22 to 9.84)	8933 (5360 to 12,471)	5.81 (3.49 to 8.1)	−0.8 (−0.92 to −0.68)

EAPC, estimated annual percentage change; SDl, Sociodemographic Index; Ul, uncertainty interval. EAPC is expressed as 95% CIs.

**Table 4:** The DALYs cases and age-standardized DALYs rate of Urogenital Congenital Anomalies in Children Under 9 Years in 1990 and 2021, along with their temporal trend.

	Rate per 100 000(95%UI)		2021		1990–2021
	1990				
	DALYs cases	The age-standardized DALYs rate	DALYs cases	The age-standardized DALYs rate	EAPC
**Global**	9362,347 (7680,195 to 11,251,785)	775.41 (636.21 to 931.68)	5122,959 (4069,356 to 6172,763)	384.1 (304.36 to 463.8)	−2.16 (−2.27 to −2.06)
**SDI region**					
High SDI	455,376 (436,571 to 475,572)	366.62 (351.4 to 382.99)	205,578 (189,349 to 222,446)	182.77 (168.1 to 198.04)	−1.96 (−2.08 to −1.85)
High-middle SDI	1754,703 (1461,995 to 2053,374)	956.67 (796.81 to 1119.93)	431,703 (348,498 to 514,392)	284.97 (228.81 to 341.31)	−4.02 (−4.21 to −3.84)
Middle SDI	3525,246 (2847,309 to 4218,608)	895.1 (722.94 to 1071.21)	1231,727 (983,290 to 1497,963)	330.83 (263.32 to 403.76)	−3.11 (−3.25 to −2.98)
Low-middle SDI	2144,650 (1538,759 to 2802,897)	639.57 (459.34 to 835.51)	1516,568 (1227,376 to 1822,153)	394.62 (319.01 to 474.49)	−1.32 (−1.4 to −1.24)
Low SDI	1475,906 (1018,095 to 1976,120)	859.74 (593.1 to 1151.74)	1733,105 (1215,694 to 2236,899)	539.52 (378.99 to 695.72)	−1.35 (−1.46 to −1.24)
**GBD region**					
Andean Latin America	93,027 (75,619 to 117,714)	906.68 (737.42 to 1146.67)	58,747 (43,731 to 76,152)	479.35 (356.46 to 621.89)	−1.74 (−1.89 to −1.6)
Australasia	10,215 (9559 to 10,935)	332.88 (311.5 to 356.36)	6399 (5505 to 7354)	169.36 (145.38 to 195)	−1.79 (−1.92 to −1.66)
Caribbean	67,765 (46,421 to 90,185)	855.84 (588.79 to 1137.26)	49,096 (32,076 to 68,439)	637.44 (415.35 to 889.66)	−0.62 (−0.76 to −0.48)
Central Asia	133,592 (117,454 to 153,037)	745.14 (656.37 to 851.79)	92,669 (76,173 to 113,393)	476.88 (392.2 to 583.23)	−1.27 (−1.36 to −1.18)
Central Europe	109,341 (101,523 to 117,569)	579.81 (538.02 to 624.08)	22,965 (20,063 to 26,369)	201.42 (175.57 to 231.81)	−3.27 (−3.41 to −3.12)
Central Latin America	333,723 (309,396 to 363,715)	752.63 (698.12 to 819.82)	148,732 (120,238 to 187,170)	357.52 (287.73 to 452.12)	−1.8 (−2.01 to −1.58)
Central Sub-Saharan Africa	88,650 (49,584 to 127,325)	456.28 (258.06 to 653.8)	89,780 (64,880 to 122,760)	218.59 (158.01 to 298.8)	−2.01 (−2.2 to −1.83)
East Asia	3180,427 (2440,766 to 3933,798)	1412.18 (1084.07 to 1745.91)	615,951 (461,791 to 799,564)	347.4 (258.64 to 454.38)	−4.66 (−4.91 to −4.41)
Eastern Europe	251,639 (237,606 to 266,263)	720.93 (680.37 to 763.19)	56,920 (51,062 to 63,103)	252.07 (224.82 to 281.25)	−3.53 (−3.77 to −3.3)
Eastern Sub-Saharan Africa	777,336 (547,107 to 1025,313)	1139.4 (801.61 to 1503.57)	854,781 (591,590 to 1154,554)	689.45 (477.72 to 930.45)	−1.38 (−1.52 to −1.24)
High-income Asia Pacific	82,924 (74,174 to 92,334)	373.56 (334.19 to 415.75)	24,623 (21,792 to 27,251)	173.84 (153.29 to 193)	−2.37 (−2.53 to −2.2)
High-income North America	134,556 (131,231 to 137,934)	318.91 (311.05 to 326.9)	78,021 (71,371 to 85,142)	184.24 (168.3 to 201.31)	−1.39 (−1.5 to −1.29)
North Africa and Middle East	695,517 (514,057 to 901,347)	702.61 (519.93 to 909.88)	442,450 (345,237 to 534,499)	356.34 (277.73 to 430.98)	−1.81 (−2 to −1.63)
Oceania	5771 (3572 to 8642)	303.08 (187.53 to 453.37)	10,872 (7031 to 16,759)	298.51 (193.11 to 459.8)	−0.01 (−0.24 to 0.21)
South Asia	1778,634 (1209,662 to 2416,792)	579.62 (394.26 to 787.56)	1132,588 (912,215 to 1408,128)	348.58 (280.36 to 434.29)	−1.52 (−1.63 to −1.41)
Southeast Asia	657,666 (417,475 to 935,915)	567.97 (359.75 to 809.53)	393,700 (306,305 to 489,635)	344.54 (267.55 to 429.43)	−1.5 (−1.54 to −1.45)
Southern Latin America	46,593 (43,298 to 49,993)	459.21 (426.73 to 492.74)	24,328 (20,732 to 28,629)	255.73 (216.99 to 302.31)	−1.41 (−1.61 to −1.21)
Southern Sub-Saharan Africa	43,050 (33,132 to 53,524)	298.73 (230.24 to 371.03)	51,483 (39,275 to 65,647)	318.85 (242.94 to 406.94)	0.63 (0.3 to 0.96)
Tropical Latin America	220,528 (191,548 to 250,140)	625.55 (543.1 to 710.53)	104,418 (79,858 to 127,456)	307.85 (235.42 to 375.82)	−1.56 (−1.96 to −1.15)
Western Europe	179,098 (173,848 to 184,388)	385.8 (374.42 to 397.28)	86,559 (79,158 to 94,441)	195.74 (178.61 to 213.99)	−2.04 (−2.15 to −1.94)
Western Sub-Saharan Africa	472,294 (367,530 to 578,724)	699.4 (543.23 to 857.6)	777,876 (466,216 to 1084,415)	505.4 (303.63 to 703.69)	−0.81 (−0.93 to −0.69)

EAPC, estimated annual percentage change; SDl, Sociodemographic Index; Ul, uncertainty interval. EAPC is expressed as 95% CIs.

### Global burden of UGCAs in children under 9 years by sex, age, SDI, and region

**Figure 2:**
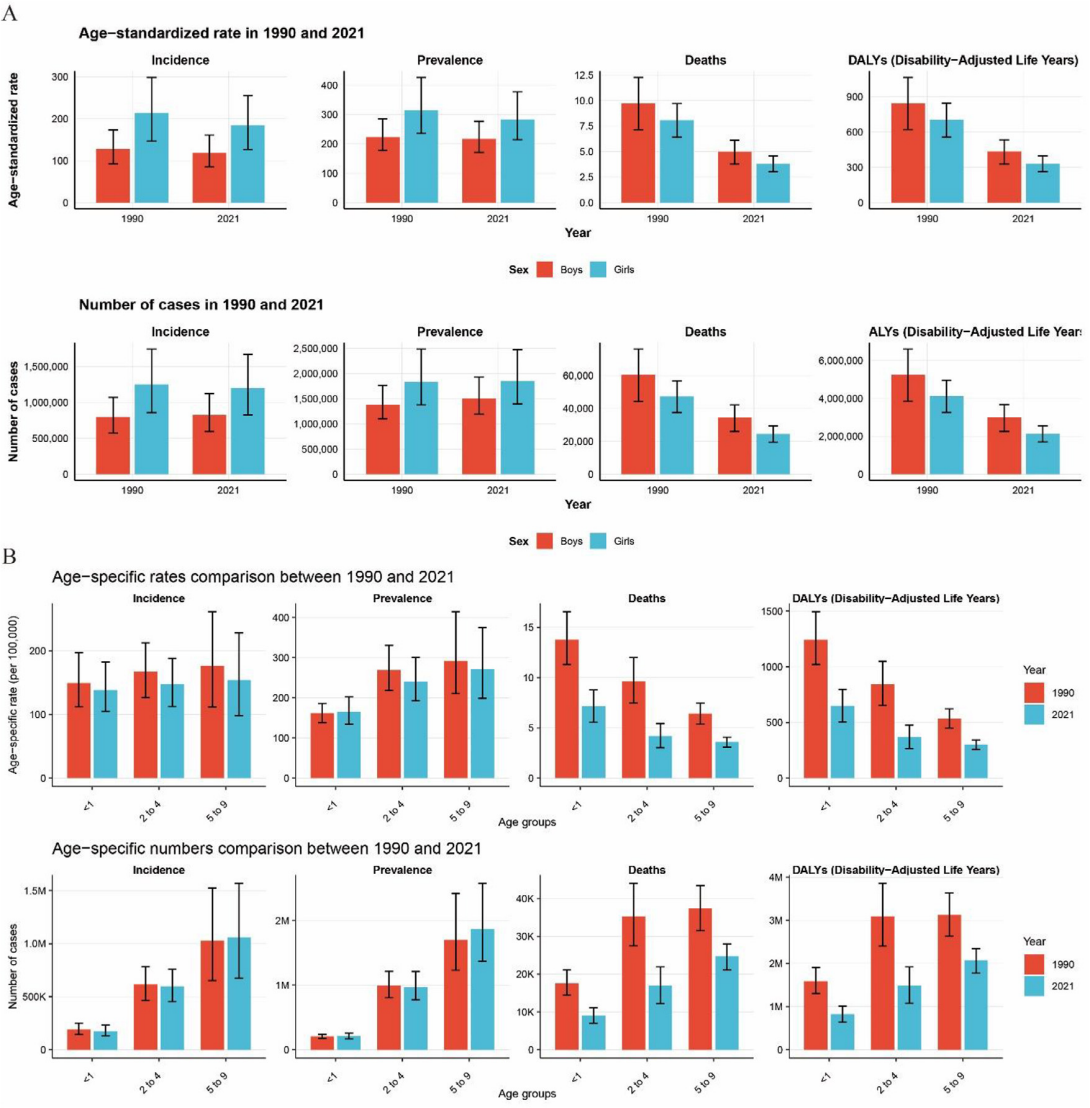
Comparison of the disease burden of urogenital congenital anomalies (UGCAs) by gender and age group for children under 9 years old in 1990 and 2021 (A. Different genders; B. Different age groups).

Trends were broadly consistent across sexes, although incidence and prevalence were higher in females, while deaths and DALYs were higher in males. In 2021, male-to-female ratios were 0.69:1 for incidence, 0.82:1 for prevalence, 1.41:1 for deaths, and 1.40:1 for DALYs; corresponding ASR ratios were 0.64:1, 0.77:1, 1.32:1, and 1.31:1. Across age groups, incidence, prevalence, deaths, and DALYs increased with age, whereas ASMR and ASDR declined, particularly in younger children. Compared with 1990, ASRs, deaths, and DALYs declined across all age groups, while total case numbers remained stable; morbidity increased in children aged 5–9 years (Figure 2).

**Figure 3:**
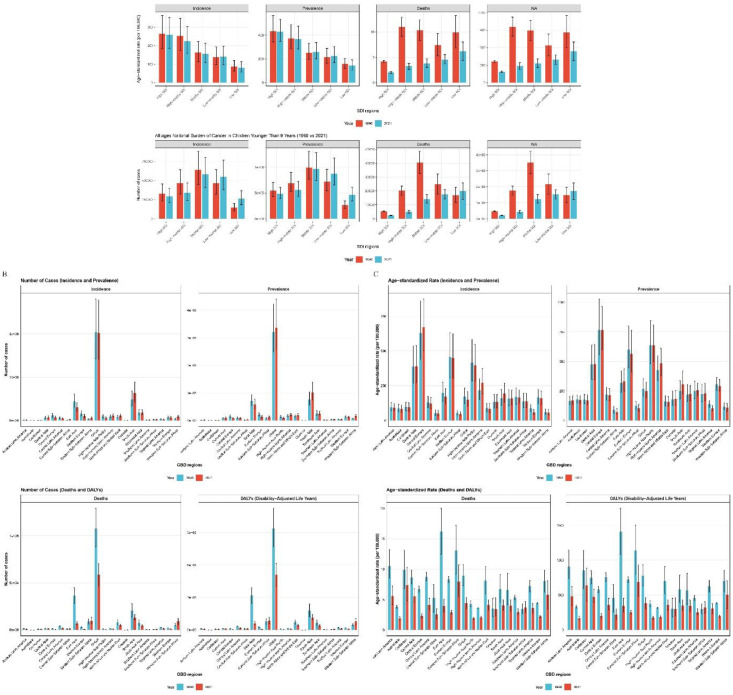
Comparison of the disease burden of urogenital congenital anomalies (UGCAs) for children under 9 years old in 1990 and 2021 across different SDI and GBD regions (A. Different SDI regions; B. Absolute numbers across different GBD regions; C. Age-Standardized Rates (ASRs) across different GBD regions).

By SDI, deaths, DALYs, and their ASRs decreased with increasing SDI, whereas ASIR and ASPR increased. Middle-SDI regions had the highest morbidity and prevalence, while high-middle and middle SDI regions experienced the greatest reductions in deaths and DALYs (Figure 3 and Tables 1, 2, Table 3, Table 4).

At the regional level, Southern Sub-Saharan Africa was the only region with increasing ASMR and ASDR. Declines were most pronounced in East Asia, Eastern and Central Europe, and high-income regions. Approximately one-third of regions showed increasing ASIRs and two-thirds increasing ASPRs, with overlapping trends in South Asia, High-income North America, Andean Latin America, and Southern Sub-Saharan Africa. In 2021, the highest ASIRs and ASPRs were observed in high-SDI regions (e.g., Central and Eastern Europe, High-income Asia Pacific, North America), whereas the highest ASMRs and ASDRs occurred in low-SDI regions (e.g., Eastern and Western Sub-Saharan Africa, Caribbean, Andean Latin America). Differences reached up to 14-fold for incidence and 10-fold for prevalence, and approximately fivefold for mortality rates. South Asia contributed the largest absolute burden, accounting for 31.36% of incident cases and ∼22% of deaths and DALYs.

**Figure 4:**
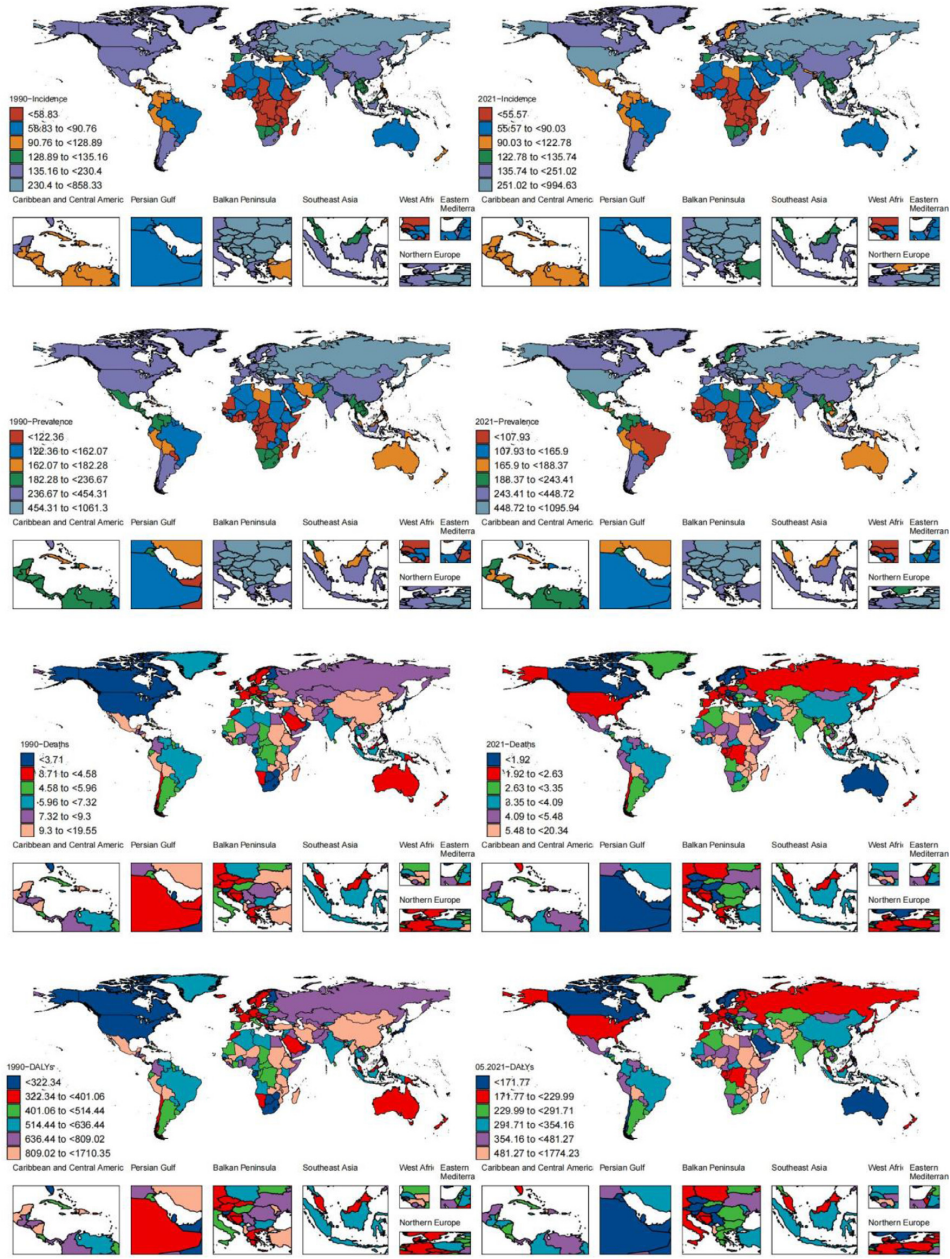
Geographical distribution of the global disease burden for urogenital congenital anomalies (UGCAs) in children under 9 years old in 1990 and 2021.

Country-level patterns were heterogeneous. High ASIRs and ASPRs were observed in parts of Europe, Central Asia, and several African countries. The highest mortality rates were concentrated in low-SDI countries, while the lowest occurred in high-SDI settings, with differences up to 18-fold. Rapid increases in ASIRs were observed in countries such as India, the United States, and Poland, whereas mortality increased in selected countries, including Zimbabwe and Botswana. Absolute burdens were highest in populous countries such as China and India (Figure 4 and Supplement Tables 1–4).

### SDI correlation analysis

**Figure 5:**
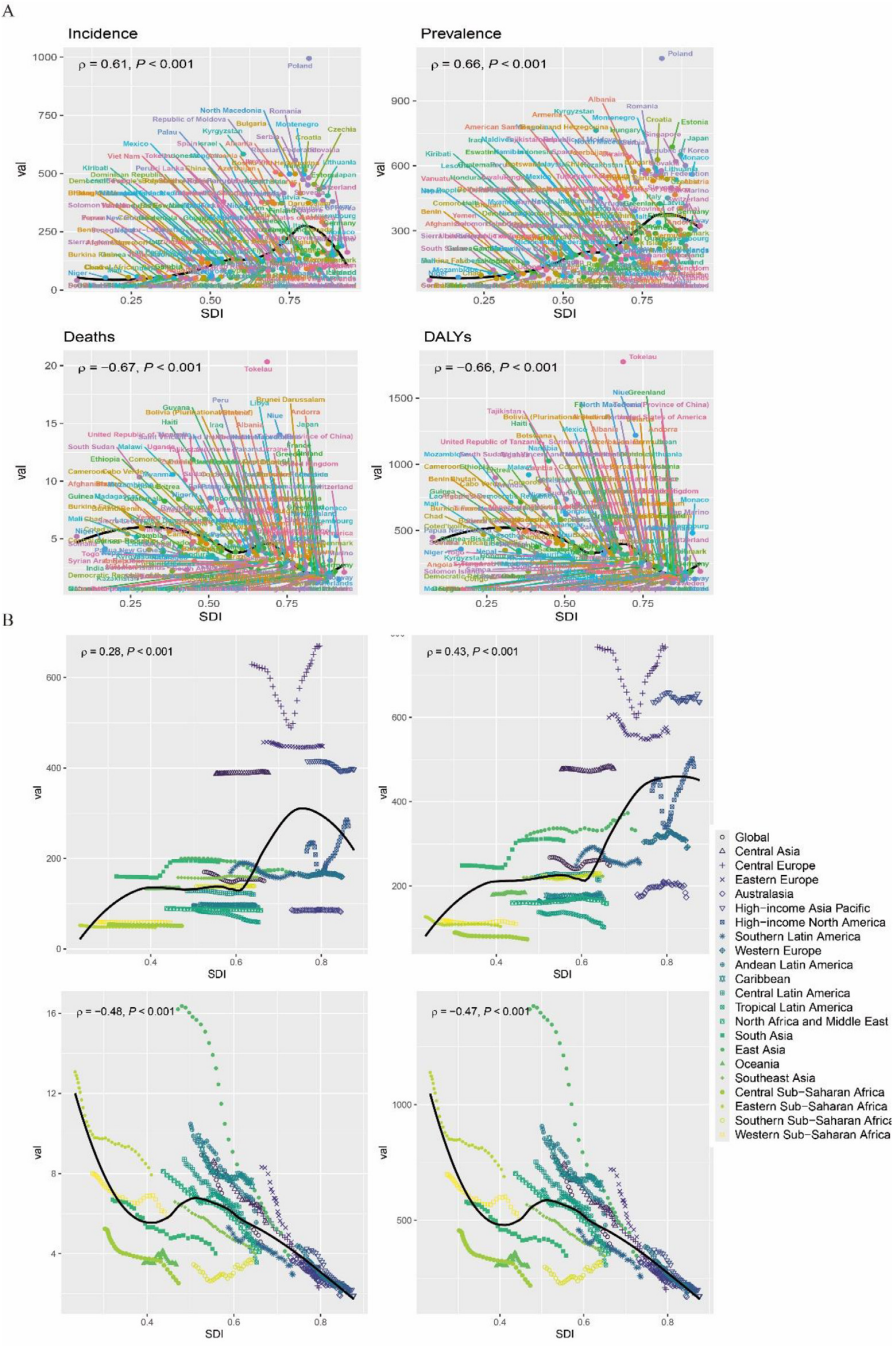
Correlation between the disease burden of urogenital congenital anomalies (UGCAs) in children under 9 years old and SDI (A. National level; B. Regional level).

UGCA burden showed consistent associations with SDI. Incidence and prevalence were positively correlated with SDI (regional *ρ* = 0.28 and 0.43; national *ρ* = 0.60 and 0.66; all *p* < 0.001), whereas deaths and DALYs were negatively correlated (regional *ρ* = −0.48 and −0.47; national *ρ* = −0.67 and −0.66; all *p* < 0.001) (Figure 5).

### Health inequities

**Figure 6:**
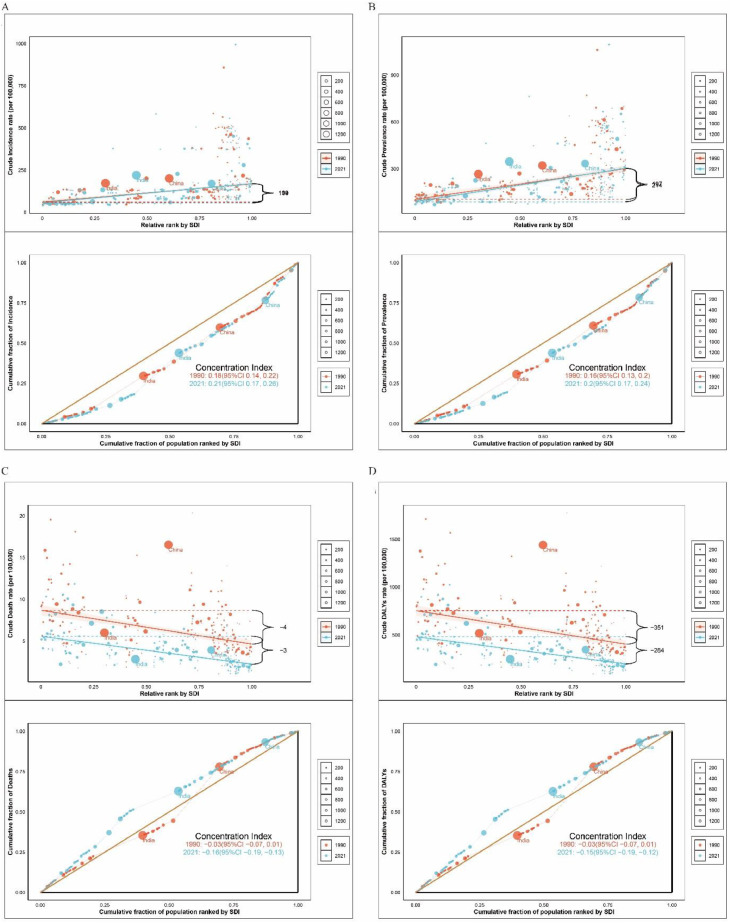
Health inequities in the changes of the disease burden for urogenital congenital anomalies (UGCAs) in children under 9 years old globally (A. Incidence; B. Prevalence; C. Deaths; D. DALYs).

Inequality analyses revealed persistent and widening disparities in the burden of UGCAs. Incidence and prevalence were increasingly concentrated in high-SDI regions, with concentration indices rising from 0.18 to 0.21 and from 0.16 to 0.20, respectively. In contrast, deaths and DALYs became more concentrated in low-SDI regions, with concentration indices declining from −0.03 to −0.16 and from −0.03 to −0.15 (Figure 6). These opposing gradients are consistent with SDI correlation analyses, which showed positive associations between SDI and incidence/prevalence but negative associations with mortality and DALYs. They are also aligned with decomposition findings, indicating that population growth and improved detection contribute to higher case identification in higher-SDI settings, whereas slower epidemiological improvements in low-SDI regions limit reductions in fatal and disability outcomes.

### Decomposition analysis

**Figure 7:**
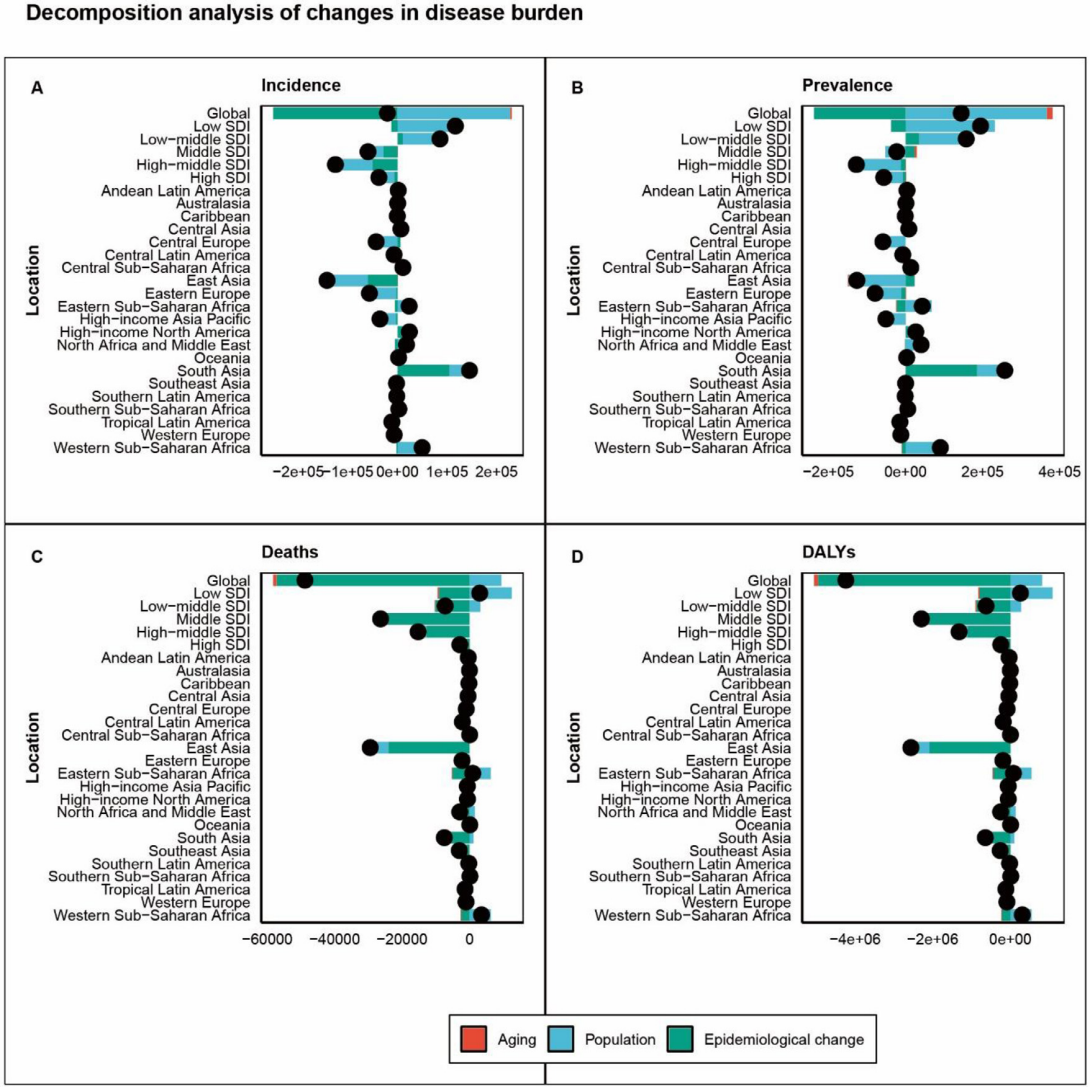
Decomposition analysis of the disease burden of urogenital congenital anomalies (UGCAs) in children under 9 years old globally in terms of aging, population growth, and epidemiological changes (A. Incidence; B. Prevalence; C. Deaths; D. DALYs).

Population growth was the main contributor to increased incidence and prevalence in low and low-middle-SDI regions (e.g., Western Sub-Saharan Africa, South Asia), but contributed negatively in higher-SDI regions. Epidemiological changes were the primary drivers of declining deaths and DALYs globally, particularly in middle and high-middle SDI regions, although their beneficial impact was weaker in low-SDI regions and reversed in South Asia. Ageing had minimal overall impact (Figure 7).

### 2035 Predictions

**Figure 8:**
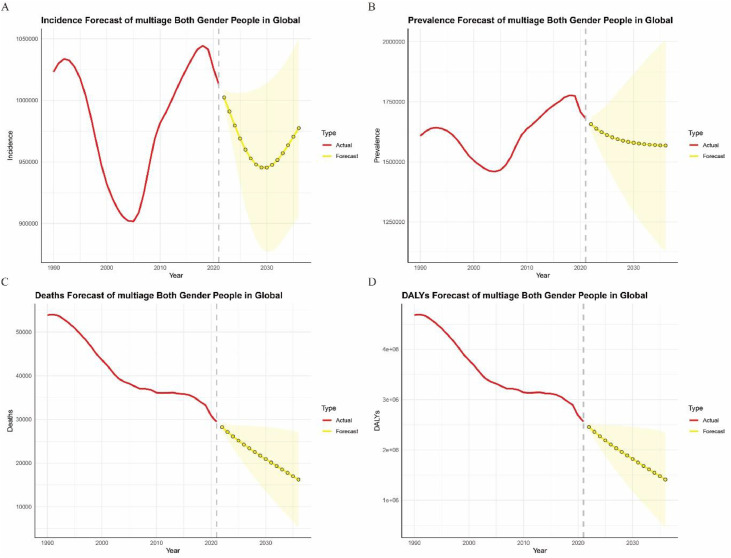
ARIMA model forecast of the disease burden of urogenital congenital anomalies (UGCAs) in children under 9 years old globally by 2035 (A. Incidence; B. Prevalence; C. Deaths; D. DALYs).

Both ARIMA and Holt–Winters models projected continued declines in UGCA-related deaths and DALYs over the forecast period. However, the two approaches yielded divergent patterns for incidence and prevalence. Specifically, ARIMA models suggested a potential increase in incidence after 2030, followed by a corresponding rise in prevalence, whereas Holt–Winters models indicated relatively stable trends. These discrepancies likely arise from differences in model structure and sensitivity to long-term trends and recent fluctuations. Accordingly, these projections should be interpreted with caution, as they reflect methodological variability rather than consistent evidence of future increases. Overall, the results highlight uncertainty in forecasting morbidity trends, while the declining trajectory of mortality appears more consistent across models (Figure 8).

## Discussion

This study provides a comprehensive synthesis of the global burden of UGCAs in children under 9 years, outlining current patterns, long-term trends since 1990, and key drivers. Three main findings emerge. First, while incidence and prevalence have remained stable or slightly increased, age-standardized mortality and disability-adjusted life years (DALYs) have declined across most regions. This divergence likely reflects population growth and improved detection alongside advances in antenatal screening, neonatal surgery, pediatric nephrology, and infection control [6,18]. Second, the burden varies markedly by sex, age, geography, and sociodemographic development, with mortality and disability disproportionately concentrated in low-SDI settings. Third, both modifiable maternal factors (e.g., pregestational diabetes, teratogenic exposure, and obesity) and health system capacity (e.g., antenatal ultrasound, surgical and nephrology services, access to dialysis/transplantation, and long-term follow-up) represent critical intervention targets [19].

The coexistence of increasing incidence/prevalence and declining mortality/DALYs suggests that expanded detection and demographic growth are enlarging the identified case pool, while improvements in care are reducing fatality and disability. Increased use of antenatal ultrasound and structured postnatal evaluation has enhanced detection of milder anomalies, particularly in high-resource settings [20,21]. Consistently, decomposition analyses indicate that population growth is the primary driver of rising case numbers, whereas reductions in age-specific fatality account for declines in deaths and DALYs, in line with Global Burden of Disease findings [3].

Age and sex gradients further clarify underlying mechanisms. Mortality is concentrated in the neonatal and early-infant period, when severe obstructive uropathies and bilateral renal dysplasia present with respiratory compromise, electrolyte imbalance, and sepsis risk [22]. Among survivors, the accumulation of nonfatal sequelae—such as recurrent urinary tract infections, renal scarring, hypertension, and growth impairment — drives increasing prevalence with age [6]. Sex differences are both biological and clinical: male-specific posterior urethral valves disproportionately increase deaths and years of life lost, whereas higher detection of vesicoureteral reflux and UTIs in girls elevates morbidity. Consequently, deaths and DALYs tend to be higher in boys, while incidence and prevalence may appear higher in girls in settings with more complete detection.

A pronounced sociodemographic gradient is evident. Higher age-standardized incidence and prevalence in high-SDI regions likely reflect more comprehensive detection and structured screening, whereas deaths and DALYs remain concentrated in low- and middle-SDI settings due to constrained access to surgery, nephrology care, and renal replacement therapies [23]. This divergence underscores persistent inequities; accordingly, outcome-based indicators—such as CKD progression, UTI-related hospitalization, and UGCA-attributable mortality—may better capture health system performance than prevalence alone [24,25].

These inequalities are closely linked to both health system capacity and demographic dynamics. In high-SDI regions, higher incidence and prevalence largely reflect more complete case ascertainment, particularly for milder conditions. In contrast, excess mortality and DALYs in low-SDI settings are driven by delayed diagnosis, limited pediatric surgical and nephrology services, and fragmented follow-up care [26] Decomposition analyses further support this interpretation, showing that although population growth increases case numbers globally, reductions in deaths and DALYs are primarily driven by epidemiological improvements that are unevenly distributed and substantially weaker in low-SDI regions. Together, these findings indicate that observed disparities arise less from differences in disease occurrence than from inequities in detection, treatment access, and health system performance. Addressing these gaps will require targeted investments in prenatal screening, pediatric surgical capacity, and long-term nephrology care in resource-limited settings.

Although no single high-impact strategy exists, several maternal exposures are consistently linked to UGCA risk, including pregestational diabetes, poor glycemic control, teratogenic medications (e.g., ACE inhibitors/ARBs), and obesity. Assisted reproductive technologies and advanced maternal age may further elevate risk, reinforcing the importance of preconception counseling and early medication review. Integrating these measures into primary and antenatal care — glycemic optimization, teratogen avoidance, and weight management — offers a scalable prevention approach across diverse settings.

Advances in genetics are refining risk stratification and clinical management. Monogenic variants (e.g., HNF1B, PAX2, EYA1, SALL1) and copy-number alterations explain a subset of cases, often accompanied by extra-renal manifestations. Targeted genetic testing can improve diagnostic precision and inform counseling; however, limited access in low-resource settings risks exacerbating existing disparities. Clinical progress has shifted UGCAs from high fatality toward chronic morbidity. Antenatal ultrasound and standardized postnatal pathways facilitate early detection and timely intervention, while advances in pediatric urology and nephrology reduce complications and help preserve renal function. Nevertheless, a substantial proportion of patients progress to chronic kidney disease, necessitating long-term follow-up and structured transition to adult care. Psychosocial and economic burdens remain considerable, underscoring the need for integrated support systems. This GBD-based analysis enables standardized cross-country comparisons over time but has important limitations. Reliance on ICD-based classification restricts phenotypic granularity, under-ascertainment in low-resource settings may introduce bias, and DALYs do not capture detailed clinical outcomes or service capacity.

## Conclusion

UGCAs serve as a sensitive indicator of pediatric health system performance and equity. Priority actions include strengthening maternal risk control and ensuring equitable access to antenatal screening, specialized care, genetic services, and long-term management to reduce CKD progression and improve outcomes.

## Ethics declaration

Ethical approval was not required for this secondary analysis of publicly available, aggregated estimates from the Global Burden of Disease (GBD) study, as no individual-level data were used.

## Authors’ contributions

Conceptualization: Sheng Gong; Methodology: Sheng Gong, Jianming Zhu,; Software and Data Curation: Sheng Gong; Formal Analysis and Visualization: Guoping Jiang, Weiwei Ruan; Validation: Guoping Jiang, Weiwei Ruan; Investigation: Guoping Jiang, Weiwei Ruan; Writing – Original Draft: Sheng Gong, Jianming Zhu; Writing – Review & Editing: Sheng Gong; Supervision: Sheng Gong; Project Administration: Sheng Gong; Funding Acquisition: Sheng Gong, Jianming Zhu. All authors have read and agreed to the published version of the manuscript.

## Funding

Medical and Health Research Project of Zhejiang Province (General Project) (2025KY1417), Ningbo Medical Key Discipline (2026-A36). The funders had no role in the study design, data collection, data analysis, data interpretation, or writing of the report. The corresponding authors had full access to all study data and had final responsibility for the decision to submit for publication.

## Data availability statement

The datasets generated and/or analyzed during the current study are derived from the Global Burden of Disease Study 2021 (GBD 2021), which is publicly available through the Institute for Health Metrics and Evaluation (IHME) GBD Results Tool at https://vizhub.healthdata.org/gbd-results/. The exact datasets used in this analysis can be replicated by applying the same selection criteria (e.g., location, year, metric, cause) as detailed in the Methods section. All data generated from the secondary analysis (e.g., calculated rates, trend analyses) are available within the article and its supplementary information files.

## Conflicts of interest

The authors declare no conflicts of interest.

## Appendix Supplementary materials

Image, application 1
