# Supplementary material for: Urogenital congenital anomalies in children under 9 years: global disease burden analysis and projections, 1990–2021
Source: J Pediatr (Rio J). 2026 Jun 15;102(5):101576. doi: 10.1016/j.jped.2026.101576 (PMC13284462; doi:10.1016/j.jped.2026.101576)
Supplement: Supplementary file 1 [file mmc1.docx]

**JPED-D-26-00008**

**Supplementary Material**

**Supplementary Table 1** The incidence cases and age-standardized incidence rate of Urogenital Congenital Anomalies in Children Under 9 Years in 1990 and 2021 across 204 countries worldwide, along with their temporal trend.

|  | Rate per 100 000(95%UI) | | 2021 | | 1990-2021 |
| --- | --- | --- | --- | --- | --- |
|  | 1990 | |  | |  |
|  | Incidence cases | The age-standardized incidence rate | Incidence cases | The age-standardized incidence rate | EAPC |

| Afghanistan | 2456 (1671 to 3435) | 82.19 (55.52 to 116) | 8118 (5582 to 11343) | 80.22 (54.9 to 112.61) | -0.03 (-0.06 to 0) |
| --- | --- | --- | --- | --- | --- |
| Albania | 3923 (2773 to 5298) | 504.01 (355.87 to 681.71) | 1468 (1028 to 1988) | 505.75 (355.26 to 683.12) | 0.01 (-0.01 to 0.02) |
| Algeria | 6212 (4344 to 8634) | 82.85 (58.04 to 114.95) | 7516 (5130 to 10548) | 80.27 (54.85 to 112.53) | -0.09 (-0.1 to -0.07) |
| American Samoa | 18 (12 to 25) | 131.8 (89.14 to 187.47) | 12 (8 to 17) | 134.76 (91.45 to 191.2) | 0.07 (0.06 to 0.08) |
| Andorra | 10 (7 to 14) | 174.35 (123.72 to 234.45) | 9 (6 to 12) | 154.44 (110.22 to 209.85) | -0.34 (-0.4 to -0.27) |
| Angola | 1954 (1335 to 2714) | 56.69 (38.43 to 79.51) | 5637 (3821 to 7990) | 51.96 (35.16 to 73.76) | -0.28 (-0.3 to -0.27) |
| Antigua and Barbuda | 11 (8 to 15) | 89.45 (62.73 to 123.27) | 10 (7 to 13) | 91.77 (64.88 to 124.91) | 0.15 (0.13 to 0.18) |
| Argentina | 11019 (7603 to 15263) | 161 (111.22 to 222.78) | 10648 (7257 to 14895) | 159.47 (109.61 to 221.27) | -0.22 (-0.36 to -0.08) |
| Armenia | 2424 (1690 to 3338) | 328.96 (228.98 to 453.63) | 1454 (1005 to 2004) | 370.32 (257.55 to 508.39) | 0.59 (0.48 to 0.7) |
| Australia | 2155 (1561 to 2913) | 85.21 (61.8 to 114.99) | 2599 (1859 to 3536) | 83.44 (59.93 to 113.05) | 0.04 (-0.01 to 0.09) |
| Austria | 2950 (2426 to 3444) | 325.81 (268.9 to 379.59) | 2523 (1986 to 3189) | 291.79 (230.06 to 368.16) | -0.32 (-0.59 to -0.06) |
| Azerbaijan | 6549 (4551 to 9048) | 381.59 (264.45 to 528.64) | 5908 (4081 to 8229) | 379.69 (264.19 to 525.05) | -0.02 (-0.03 to -0.01) |
| Bahamas | 49 (34 to 67) | 90.95 (64.33 to 124.8) | 43 (30 to 59) | 90.69 (63.72 to 124.36) | 0 (-0.03 to 0.03) |
| Bahrain | 92 (63 to 130) | 78.21 (53.48 to 110.86) | 155 (105 to 221) | 78.1 (53.4 to 110.93) | 0.02 (0 to 0.04) |
| Bangladesh | 42224 (29320 to 57861) | 119.29 (82.52 to 164.02) | 34663 (23813 to 48151) | 115.66 (79.83 to 160.06) | -0.08 (-0.1 to -0.07) |
| Barbados | 38 (27 to 52) | 94.63 (67.94 to 128.68) | 27 (19 to 37) | 93.6 (67.22 to 127.13) | 0.03 (-0.01 to 0.06) |
| Belarus | 6815 (4687 to 9516) | 416.08 (286.95 to 579.26) | 4336 (2960 to 6112) | 414.83 (285.86 to 578.72) | 0.02 (0 to 0.03) |
| Belgium | 1953 (1402 to 2651) | 163.47 (117.49 to 221.56) | 2004 (1434 to 2711) | 161.84 (116.25 to 218.17) | -0.54 (-1.2 to 0.13) |
| Belize | 54 (39 to 74) | 94.33 (67.25 to 128.1) | 71 (49 to 97) | 89.29 (62.19 to 122.84) | -0.13 (-0.15 to -0.11) |
| Benin | 1036 (739 to 1432) | 57.05 (40.35 to 79.33) | 2431 (1681 to 3404) | 55.7 (38.31 to 78.32) | -0.05 (-0.07 to -0.03) |
| Bermuda | 8 (5 to 10) | 92.82 (65.5 to 126.18) | 5 (4 to 7) | 99.89 (73.28 to 132.99) | 0.33 (0.27 to 0.38) |
| Bhutan | 212 (145 to 291) | 117.04 (79.82 to 161.3) | 143 (98 to 199) | 116.55 (80.48 to 161.84) | -0.04 (-0.07 to -0.02) |
| Bolivia (Plurinational State of) | 1960 (1424 to 2611) | 102.97 (74.62 to 137.64) | 2306 (1670 to 3175) | 97.35 (70.55 to 133.93) | -0.18 (-0.19 to -0.17) |
| Bosnia and Herzegovina | 3588 (2504 to 4903) | 498.57 (348.9 to 678.98) | 1565 (1089 to 2144) | 496.01 (346.57 to 676) | -0.01 (-0.01 to 0) |
| Botswana | 528 (354 to 756) | 129.31 (86.5 to 185.38) | 619 (419 to 884) | 131.69 (89.23 to 187.8) | 0.4 (-0.01 to 0.81) |
| Brazil | 30543 (22753 to 39921) | 88.65 (66.32 to 115.38) | 19101 (14045 to 25514) | 58.61 (43.11 to 78.27) | -1.37 (-1.51 to -1.23) |
| Brunei Darussalam | 241 (166 to 338) | 373.38 (256.41 to 525.45) | 235 (160 to 331) | 373.78 (255.32 to 524.88) | 0 (-0.02 to 0.01) |
| Bulgaria | 5511 (3865 to 7509) | 503.18 (353.9 to 683.56) | 3111 (2166 to 4253) | 498.08 (347.92 to 678.26) | -0.03 (-0.03 to -0.02) |
| Burkina Faso | 1971 (1384 to 2725) | 57.09 (39.78 to 79.46) | 4177 (2922 to 5803) | 55.81 (38.74 to 78.1) | -0.01 (-0.04 to 0.02) |
| Burundi | 979 (736 to 1283) | 50.02 (37.35 to 66.04) | 1745 (1222 to 2451) | 42.41 (29.65 to 59.67) | -0.36 (-0.44 to -0.28) |
| Cabo Verde | 61 (42 to 86) | 53.78 (36.88 to 76.58) | 53 (36 to 74) | 56.82 (39.21 to 79.41) | 0.17 (0.14 to 0.21) |
| Cambodia | 4562 (3142 to 6348) | 134.99 (92.59 to 188.82) | 4562 (3107 to 6422) | 131.66 (89.73 to 185.2) | -0.1 (-0.11 to -0.08) |
| Cameroon | 1997 (1411 to 2766) | 55.68 (38.89 to 77.79) | 5161 (3556 to 7219) | 54.86 (37.74 to 76.83) | 0.02 (-0.01 to 0.04) |
| Canada | 7796 (5380 to 10951) | 201.62 (139.43 to 282.69) | 7936 (5363 to 11217) | 197.13 (134.19 to 277.03) | -0.03 (-0.05 to -0.01) |
| Central African Republic | 489 (338 to 682) | 54.54 (37.32 to 76.72) | 834 (565 to 1173) | 52.55 (35.5 to 74.1) | -0.11 (-0.12 to -0.1) |
| Chad | 1191 (839 to 1644) | 54.82 (38.09 to 76.53) | 3605 (2500 to 5005) | 54.72 (37.66 to 76.57) | 0.03 (0.02 to 0.05) |
| Chile | 5233 (3590 to 7309) | 189.53 (129.84 to 265.14) | 4320 (3380 to 5506) | 180.76 (142.69 to 228.54) | -1.15 (-1.46 to -0.84) |
| China | 433279 (303167 to 597288) | 200.62 (140.13 to 276.97) | 295343 (208307 to 404983) | 170.5 (121.75 to 231.42) | -0.67 (-0.77 to -0.57) |
| Colombia | 9671 (6922 to 13112) | 120.25 (85.91 to 163.32) | 8374 (5978 to 11440) | 120.66 (86.26 to 164.53) | 0.19 (0.14 to 0.25) |
| Comoros | 73 (54 to 98) | 48.04 (35.44 to 64.59) | 75 (55 to 103) | 46.59 (34.04 to 63.88) | -0.11 (-0.19 to -0.03) |
| Congo | 395 (270 to 554) | 53.61 (36.42 to 75.46) | 663 (440 to 948) | 51.16 (34.13 to 72.97) | -0.16 (-0.17 to -0.15) |
| Cook Islands | 6 (4 to 8) | 130.56 (87.71 to 186.23) | 3 (2 to 5) | 134.73 (90.14 to 192.37) | 0.03 (0 to 0.05) |
| Costa Rica | 939 (674 to 1275) | 119.45 (85.69 to 162.44) | 778 (551 to 1066) | 118.51 (84.42 to 161.43) | 0.03 (0.01 to 0.04) |
| Cote d’Ivoire | 2270 (1576 to 3190) | 54.01 (37.18 to 76.39) | 4370 (2982 to 6221) | 53.18 (36.15 to 75.93) | -0.03 (-0.06 to 0) |
| Croatia | 3219 (2251 to 4453) | 507.38 (356.06 to 698.44) | 1937 (1469 to 2468) | 513.78 (390.82 to 652.98) | 0.54 (0.35 to 0.73) |
| Cuba | 1596 (1147 to 2164) | 94.36 (67.6 to 128.28) | 1077 (758 to 1475) | 92.7 (65.54 to 126.41) | 0.01 (-0.02 to 0.03) |
| Cyprus | 130 (93 to 178) | 98.51 (70.38 to 133.92) | 147 (104 to 202) | 99.24 (70.47 to 136.26) | 0.33 (0.19 to 0.47) |
| Czechia | 6091 (4219 to 8375) | 455.7 (316.81 to 624.57) | 5090 (3519 to 6997) | 453.55 (313.99 to 622.82) | -0.52 (-0.88 to -0.16) |
| Democratic People's Republic of Korea | 6754 (4774 to 9416) | 160.41 (112.39 to 225.35) | 4837 (3312 to 6849) | 156.55 (107.58 to 220.93) | -0.06 (-0.07 to -0.04) |
| Democratic Republic of the Congo | 6922 (4772 to 9649) | 53.47 (36.52 to 75.22) | 13326 (8901 to 19029) | 50.47 (33.69 to 72.11) | -0.16 (-0.17 to -0.14) |
| Denmark | 864 (616 to 1181) | 154.31 (109.8 to 211.01) | 949 (682 to 1301) | 153.9 (110.58 to 210.75) | 0.01 (-0.07 to 0.09) |
| Djibouti | 58 (43 to 79) | 47.91 (35.46 to 65.56) | 126 (89 to 175) | 44.31 (31.44 to 61.71) | -0.2 (-0.29 to -0.12) |
| Dominica | 15 (11 to 21) | 90.64 (63.91 to 124.51) | 8 (6 to 11) | 101.24 (74.08 to 135.54) | 0.39 (0.36 to 0.42) |
| Dominican Republic | 1819 (1318 to 2440) | 97.5 (70.38 to 131.3) | 1880 (1342 to 2545) | 93.62 (66.8 to 126.82) | -0.13 (-0.15 to -0.1) |
| Ecuador | 2285 (1783 to 2869) | 86.47 (67.51 to 108.6) | 2829 (2284 to 3494) | 83.97 (68.01 to 103.4) | 0.13 (0.01 to 0.26) |
| Egypt | 12851 (9043 to 17740) | 82.6 (57.85 to 114.63) | 20832 (14487 to 29417) | 80.81 (56.23 to 114.05) | 0 (-0.05 to 0.05) |
| El Salvador | 1749 (1254 to 2385) | 118.74 (84.99 to 162.26) | 1416 (1002 to 1958) | 115.07 (81.67 to 158.7) | -0.09 (-0.1 to -0.08) |
| Equatorial Guinea | 76 (53 to 107) | 52.91 (36.04 to 74.57) | 197 (133 to 280) | 50.85 (34.39 to 71.84) | -0.17 (-0.19 to -0.14) |
| Eritrea | 536 (385 to 731) | 46.69 (33.39 to 64.03) | 806 (584 to 1104) | 45.66 (33.04 to 62.69) | -0.07 (-0.12 to -0.02) |
| Estonia | 1016 (707 to 1404) | 427.37 (297.31 to 590.37) | 605 (428 to 837) | 432.42 (306.64 to 596.98) | 0.08 (0.03 to 0.13) |
| Eswatini | 356 (241 to 507) | 130.65 (88.05 to 186.78) | 365 (247 to 521) | 130.73 (88.61 to 186.08) | 0 (0 to 0.01) |
| Ethiopia | 10053 (6638 to 13761) | 56.62 (37.39 to 77.76) | 15085 (10986 to 20668) | 49.47 (35.96 to 67.89) | -0.49 (-0.59 to -0.4) |
| Fiji | 257 (173 to 366) | 133.65 (90.19 to 189.72) | 252 (171 to 357) | 136.64 (92.93 to 192.92) | 0.07 (0.05 to 0.08) |
| Finland | 1039 (719 to 1431) | 162.65 (112.99 to 223.4) | 892 (621 to 1220) | 167.46 (117.41 to 227.44) | -0.23 (-0.47 to 0) |
| France | 11822 (8342 to 16038) | 150.44 (106.39 to 203.69) | 11421 (8136 to 15563) | 154.06 (110.3 to 209.14) | 0.12 (0.1 to 0.14) |
| Gabon | 151 (103 to 213) | 52.32 (35.57 to 74.08) | 225 (152 to 319) | 51.88 (35.14 to 73.54) | 0.02 (0 to 0.04) |
| Gambia | 194 (138 to 267) | 57.26 (40.44 to 79.49) | 373 (257 to 524) | 54.4 (37.32 to 76.53) | -0.2 (-0.24 to -0.16) |
| Georgia | 2730 (1895 to 3752) | 294.39 (204.48 to 404.37) | 1113 (861 to 1418) | 222.03 (172.18 to 282.08) | -1.23 (-1.43 to -1.03) |
| Germany | 16086 (11369 to 22214) | 183.14 (129.43 to 252.9) | 14811 (10455 to 20440) | 183.43 (129.66 to 252.77) | 0.44 (0.13 to 0.75) |
| Ghana | 2782 (1963 to 3825) | 57.27 (40.14 to 79.17) | 4774 (3299 to 6767) | 53.08 (36.64 to 75.31) | -0.31 (-0.37 to -0.25) |
| Greece | 2098 (1503 to 2855) | 168.15 (121.75 to 227.07) | 1356 (1009 to 1756) | 155.92 (116.41 to 201.29) | -0.54 (-0.79 to -0.28) |
| Greenland | 20 (14 to 28) | 193.64 (131.17 to 273.29) | 15 (10 to 21) | 187.97 (125.66 to 267.13) | -0.1 (-0.12 to -0.09) |
| Grenada | 22 (15 to 30) | 93.42 (66.2 to 127.18) | 13 (9 to 18) | 93.45 (67.35 to 127.05) | 0.05 (0.02 to 0.08) |
| Guam | 40 (27 to 56) | 134.8 (91.7 to 191.3) | 33 (23 to 47) | 136.72 (93.7 to 193.4) | 0.14 (0.11 to 0.16) |
| Guatemala | 3476 (2497 to 4728) | 119.46 (85.6 to 162.92) | 3783 (2656 to 5238) | 115.26 (81.32 to 158.77) | -0.07 (-0.09 to -0.06) |
| Guinea | 1297 (950 to 1737) | 62.49 (45.17 to 84.69) | 2404 (1669 to 3384) | 55.94 (38.73 to 78.91) | -0.28 (-0.31 to -0.25) |
| Guinea-Bissau | 202 (142 to 277) | 57.63 (40.34 to 79.71) | 334 (228 to 474) | 52.69 (35.82 to 74.95) | -0.25 (-0.3 to -0.21) |
| Guyana | 183 (129 to 249) | 90.27 (63.39 to 124.1) | 132 (92 to 181) | 90.18 (63.35 to 123.94) | 0.09 (0.05 to 0.13) |
| Haiti | 2071 (1438 to 2747) | 105.85 (73.37 to 141.05) | 3013 (2127 to 4024) | 99.87 (70.38 to 133.54) | -0.12 (-0.15 to -0.09) |
| Honduras | 1873 (1349 to 2549) | 119.53 (85.93 to 163.01) | 2487 (1749 to 3444) | 113.6 (80 to 157.12) | -0.18 (-0.19 to -0.17) |
| Hungary | 6382 (4465 to 8721) | 501.81 (352.27 to 683.28) | 4546 (3172 to 6199) | 499.11 (348.77 to 679.57) | -0.01 (-0.02 to -0.01) |
| Iceland | 60 (43 to 81) | 140.76 (101.83 to 190.22) | 58 (41 to 80) | 132.18 (93.48 to 182.62) | -0.18 (-0.26 to -0.1) |
| India | 396691 (273100 to 553661) | 172.46 (118.82 to 240.55) | 515800 (344061 to 725065) | 220.08 (147.83 to 307.82) | 1.15 (0.93 to 1.36) |
| Indonesia | 91050 (61661 to 128507) | 199.64 (135.64 to 280.9) | 101140 (71427 to 138134) | 227.59 (161.14 to 310.06) | 0.62 (0.5 to 0.74) |
| Iran (Islamic Republic of) | 16401 (11605 to 22416) | 90.17 (64.08 to 122.68) | 11817 (8069 to 16608) | 85.94 (59.18 to 119.99) | 0.16 (0.06 to 0.27) |
| Iraq | 4915 (3457 to 6782) | 83.93 (58.85 to 116.26) | 7663 (5368 to 10626) | 85.4 (60.15 to 117.85) | 0.15 (0.11 to 0.18) |
| Ireland | 933 (653 to 1274) | 149.32 (105.29 to 202.72) | 932 (642 to 1279) | 146.43 (101.45 to 200.1) | 0.04 (0.01 to 0.08) |
| Israel | 1569 (1102 to 2122) | 151.89 (106.85 to 205.18) | 2651 (1840 to 3644) | 146.4 (101.68 to 201.21) | -0.04 (-0.07 to 0) |
| Italy | 12076 (8612 to 16411) | 212.38 (152.07 to 287.41) | 11553 (8699 to 14985) | 245.29 (185.89 to 316.02) | 0.44 (0.24 to 0.64) |
| Jamaica | 540 (387 to 728) | 96.06 (68.93 to 129.38) | 336 (238 to 461) | 92.07 (65.38 to 125.52) | -0.12 (-0.14 to -0.1) |
| Japan | 62812 (42813 to 88740) | 436.46 (300.06 to 611.76) | 39850 (28251 to 54829) | 404.68 (289.3 to 552.94) | -0.37 (-0.44 to -0.31) |
| Jordan | 672 (481 to 903) | 58.87 (42.07 to 79.28) | 1484 (1124 to 1919) | 63.82 (48.5 to 82.26) | 0.35 (0.3 to 0.4) |
| Kazakhstan | 13656 (9490 to 18905) | 379.32 (263.02 to 526.23) | 14107 (9727 to 19612) | 373.03 (256.97 to 519.06) | -0.04 (-0.05 to -0.03) |
| Kenya | 3685 (2652 to 5028) | 46.17 (33.04 to 63.3) | 5458 (3868 to 7634) | 44.5 (31.68 to 62.01) | -0.1 (-0.13 to -0.07) |
| Kiribati | 29 (19 to 41) | 133.15 (89.48 to 188.86) | 38 (25 to 54) | 132.53 (88.95 to 188.17) | -0.02 (-0.03 to -0.01) |
| Kuwait | 347 (247 to 475) | 88.58 (63 to 121.48) | 480 (337 to 670) | 85.34 (60.16 to 118.38) | 0.04 (-0.03 to 0.1) |
| Kyrgyzstan | 7015 (4796 to 9689) | 585.31 (398.45 to 811.74) | 9263 (6286 to 12876) | 583.22 (396.41 to 809.47) | 0.03 (0.01 to 0.05) |
| Lao People's Democratic Republic | 1784 (1228 to 2471) | 135.1 (92.63 to 188.03) | 2090 (1428 to 2933) | 131.64 (89.78 to 185.08) | -0.09 (-0.09 to -0.08) |
| Latvia | 1535 (1058 to 2134) | 387.62 (266.96 to 538.99) | 749 (510 to 1052) | 380.71 (260.54 to 531.71) | -0.25 (-0.36 to -0.14) |
| Lebanon | 589 (410 to 822) | 80.74 (56.08 to 113.26) | 722 (503 to 1011) | 84.65 (59.21 to 117.97) | 0.18 (0.14 to 0.21) |
| Lesotho | 611 (409 to 876) | 128.47 (85.83 to 184.28) | 547 (368 to 784) | 130.53 (88.2 to 186.45) | 0.07 (0.06 to 0.08) |
| Liberia | 495 (345 to 687) | 58.8 (40.68 to 82.17) | 800 (547 to 1130) | 53.65 (36.67 to 75.81) | -0.31 (-0.42 to -0.2) |
| Libya | 1060 (748 to 1470) | 85.94 (60.62 to 119.32) | 855 (599 to 1177) | 92.04 (65 to 125.73) | 0.37 (0.31 to 0.43) |
| Lithuania | 2521 (1730 to 3486) | 444.3 (304.87 to 614.4) | 1203 (819 to 1675) | 440.2 (301.24 to 610.18) | -0.56 (-0.89 to -0.24) |
| Luxembourg | 73 (52 to 100) | 162.6 (115.55 to 222.48) | 106 (75 to 147) | 158.8 (112.15 to 218.8) | -0.27 (-1.02 to 0.49) |
| Madagascar | 1865 (1380 to 2470) | 47.14 (34.63 to 62.85) | 3465 (2465 to 4832) | 42.91 (30.55 to 59.81) | -0.22 (-0.27 to -0.18) |
| Malawi | 2063 (1613 to 2666) | 60.37 (46.74 to 78.87) | 2732 (1864 to 3898) | 50.51 (34.47 to 72.02) | -0.49 (-0.55 to -0.43) |
| Malaysia | 5975 (4050 to 8442) | 130.13 (88.08 to 184.13) | 6494 (4382 to 9243) | 129.41 (87.58 to 183.59) | 0 (-0.01 to 0.02) |
| Maldives | 102 (70 to 142) | 132.4 (90.99 to 185.42) | 89 (61 to 126) | 133.53 (91.74 to 187.59) | 0.06 (0.04 to 0.07) |
| Mali | 1917 (1395 to 2571) | 61.9 (44.5 to 83.97) | 4839 (3384 to 6702) | 57.64 (40 to 80.35) | -0.2 (-0.23 to -0.17) |
| Malta | 72 (52 to 96) | 124.86 (90.1 to 167.46) | 58 (43 to 77) | 134.14 (98.22 to 176.51) | -0.03 (-0.25 to 0.19) |
| Marshall Islands | 20 (13 to 29) | 131.19 (87.93 to 187.45) | 15 (10 to 22) | 132.92 (89.54 to 188.91) | 0.05 (0.04 to 0.05) |
| Mauritania | 365 (251 to 508) | 54.29 (37.02 to 76.15) | 691 (469 to 975) | 53.64 (36.42 to 75.76) | -0.07 (-0.09 to -0.04) |
| Mauritius | 275 (186 to 393) | 129.58 (87.78 to 184.29) | 169 (114 to 241) | 128.93 (87.23 to 183.37) | -0.04 (-0.07 to -0.01) |
| Mexico | 31226 (22401 to 42381) | 137.5 (98.49 to 186.9) | 25533 (18269 to 34789) | 122.77 (88.31 to 166.38) | -0.45 (-0.5 to -0.39) |
| Micronesia (Federated States of) | 42 (28 to 59) | 132.1 (89.06 to 187.85) | 26 (17 to 37) | 132.54 (88.99 to 188.71) | 0.02 (0.01 to 0.02) |
| Monaco | 4 (3 to 6) | 179.9 (131.58 to 245.73) | 6 (5 to 8) | 186.63 (137.27 to 251.36) | -0.23 (-0.39 to -0.08) |
| Mongolia | 2434 (1702 to 3346) | 384.03 (267.26 to 530.2) | 2921 (2010 to 4072) | 373.74 (257.58 to 520.3) | -0.09 (-0.1 to -0.07) |
| Montenegro | 543 (381 to 738) | 507.51 (357.37 to 687.86) | 362 (252 to 495) | 495.76 (345.77 to 675.62) | -0.06 (-0.07 to -0.05) |
| Morocco | 5284 (3604 to 7507) | 78.06 (53.11 to 111.21) | 5060 (3413 to 7231) | 77.1 (52.13 to 109.94) | -0.01 (-0.02 to 0) |
| Mozambique | 2392 (1784 to 3189) | 53 (39.31 to 71.03) | 4571 (3227 to 6514) | 45.37 (32.01 to 64.69) | -0.4 (-0.46 to -0.34) |
| Myanmar | 13755 (9456 to 19222) | 137.46 (94.56 to 191.88) | 13953 (9699 to 19543) | 133.92 (93.22 to 187.31) | -0.1 (-0.11 to -0.09) |
| Namibia | 548 (372 to 776) | 130.74 (88.19 to 186.15) | 746 (508 to 1058) | 134.33 (91.54 to 190.09) | 0.11 (0.09 to 0.13) |
| Nauru | 4 (3 to 6) | 134.07 (91.15 to 189.82) | 4 (3 to 5) | 135.2 (92.14 to 191.11) | 0.02 (0.02 to 0.03) |
| Nepal | 5408 (3773 to 7332) | 89.3 (62.01 to 121.66) | 6625 (4718 to 9055) | 107.9 (76.88 to 147.4) | 0.32 (-0.15 to 0.79) |
| Netherlands | 2804 (2009 to 3784) | 153.7 (110.07 to 207.49) | 2584 (1804 to 3509) | 148.25 (103.68 to 200.89) | -0.11 (-0.14 to -0.09) |
| New Zealand | 489 (348 to 665) | 91.2 (64.72 to 124.25) | 492 (363 to 654) | 76.67 (56.81 to 101.6) | -0.27 (-0.36 to -0.19) |
| Nicaragua | 1578 (1147 to 2135) | 122.61 (89.04 to 166) | 1530 (1076 to 2110) | 115.42 (81.39 to 158.75) | -0.15 (-0.16 to -0.13) |
| Niger | 1809 (1295 to 2465) | 59.38 (42.09 to 81.61) | 4927 (3379 to 6906) | 53.08 (36.11 to 74.89) | -0.35 (-0.39 to -0.32) |
| Nigeria | 17982 (13153 to 24428) | 63.56 (45.98 to 87.29) | 44418 (31022 to 61604) | 62.68 (43.68 to 87.14) | 0.02 (-0.01 to 0.06) |
| Niue | 1 (1 to 1) | 134.41 (90.77 to 190.85) | 0 (0 to 1) | 166.01 (118.84 to 224.47) | 0.3 (0.16 to 0.45) |
| North Macedonia | 1760 (1237 to 2392) | 505.6 (356.61 to 685.08) | 1056 (735 to 1446) | 497.78 (348.28 to 677.67) | -0.03 (-0.04 to -0.02) |
| Northern Mariana Islands | 11 (8 to 16) | 132.45 (88.95 to 188.8) | 9 (6 to 13) | 132.55 (89.46 to 188.44) | 0.02 (0 to 0.03) |
| Norway | 1045 (740 to 1432) | 195.91 (138.67 to 268.93) | 1123 (779 to 1565) | 188.7 (131.68 to 261.23) | -0.39 (-0.6 to -0.18) |
| Oman | 470 (320 to 662) | 77.36 (52.39 to 109.28) | 676 (454 to 967) | 77.25 (52.11 to 110.21) | 0.05 (0.01 to 0.09) |
| Pakistan | 45680 (31986 to 62936) | 130.82 (91.34 to 180.77) | 78221 (55219 to 107837) | 133.28 (94.14 to 183.66) | 0.11 (0.09 to 0.14) |
| Palau | 4 (3 to 6) | 132.42 (89.21 to 188.6) | 3 (2 to 4) | 132.41 (89.38 to 188.3) | -0.01 (-0.02 to -0.01) |
| Palestine | 588 (412 to 813) | 83.91 (58.47 to 116.84) | 1055 (743 to 1460) | 83.78 (59.19 to 115.55) | 0.08 (0.04 to 0.13) |
| Panama | 688 (496 to 934) | 121.49 (87.67 to 164.79) | 937 (678 to 1278) | 121.63 (88.28 to 165.08) | -0.03 (-0.04 to -0.01) |
| Papua New Guinea | 1596 (1081 to 2249) | 132.74 (89.42 to 187.98) | 3723 (2533 to 5228) | 133.42 (90.19 to 188.46) | 0.01 (0 to 0.02) |
| Paraguay | 869 (613 to 1195) | 73.28 (51.65 to 100.98) | 969 (684 to 1341) | 73.15 (51.81 to 100.9) | 0.06 (0.01 to 0.11) |
| Peru | 5645 (4082 to 7605) | 99.24 (71.71 to 133.79) | 6240 (4460 to 8589) | 95.98 (68.62 to 132.08) | -0.04 (-0.07 to -0.01) |
| Philippines | 21329 (14851 to 29032) | 121.59 (84.44 to 165.86) | 17571 (12504 to 24072) | 77.67 (55.37 to 106.18) | -1.95 (-2.28 to -1.62) |
| Poland | 54114 (37208 to 75068) | 858.33 (594.96 to 1182.3) | 37842 (29136 to 47680) | 994.63 (766.92 to 1251.46) | 0.84 (0.16 to 1.53) |
| Portugal | 1768 (1274 to 2397) | 139.45 (101.19 to 187.97) | 1313 (952 to 1780) | 153.39 (111.47 to 207.62) | 0.42 (0.3 to 0.54) |
| Puerto Rico | 591 (420 to 808) | 91.62 (65.23 to 125.06) | 227 (157 to 317) | 89.72 (62.9 to 123.26) | 0.02 (-0.01 to 0.05) |
| Qatar | 78 (53 to 110) | 87.2 (58.97 to 123.07) | 310 (211 to 437) | 88.54 (60.22 to 124.86) | -0.06 (-0.19 to 0.07) |
| Republic of Korea | 27225 (18532 to 38537) | 373.3 (256.35 to 523.84) | 14262 (9604 to 20269) | 379.72 (259.97 to 530.77) | 0.09 (0.08 to 0.1) |
| Republic of Moldova | 3652 (2548 to 5048) | 428.46 (299.07 to 591.8) | 1389 (953 to 1952) | 415.4 (287.14 to 578.81) | -0.08 (-0.11 to -0.06) |
| Romania | 19832 (14052 to 27112) | 564.92 (400.66 to 771.6) | 10668 (7506 to 14678) | 557.28 (393.4 to 764.27) | -0.17 (-0.26 to -0.08) |
| Russian Federation | 109079 (73912 to 151225) | 461.03 (313.4 to 637.57) | 77470 (51444 to 108720) | 450.71 (302.84 to 626.77) | -0.08 (-0.1 to -0.06) |
| Rwanda | 1298 (982 to 1707) | 52 (39.15 to 68.77) | 1537 (1106 to 2122) | 45.15 (32.49 to 62.41) | -0.52 (-0.61 to -0.44) |
| Saint Kitts and Nevis | 9 (6 to 12) | 91.12 (64.61 to 124.85) | 6 (4 to 8) | 92.19 (65.42 to 125.17) | 0.08 (0.05 to 0.11) |
| Saint Lucia | 32 (23 to 44) | 92.2 (65.04 to 125.77) | 17 (12 to 24) | 93.09 (66.3 to 125.88) | 0.03 (0 to 0.05) |
| Saint Vincent and the Grenadines | 26 (19 to 35) | 95.55 (69.4 to 129.39) | 15 (11 to 21) | 95.18 (68.61 to 128.45) | -0.01 (-0.05 to 0.03) |
| Samoa | 65 (45 to 92) | 134.8 (92.05 to 190.51) | 76 (51 to 107) | 135.3 (91.54 to 191.21) | 0.01 (0 to 0.02) |
| San Marino | 5 (4 to 7) | 202.77 (147.07 to 265.17) | 4 (3 to 6) | 166.28 (120.96 to 224.95) | -0.57 (-0.61 to -0.53) |
| Sao Tome and Principe | 23 (16 to 31) | 56.65 (39.82 to 78.52) | 27 (18 to 38) | 51.92 (34.99 to 73.87) | -0.24 (-0.32 to -0.17) |
| Saudi Arabia | 3669 (2521 to 5146) | 78.7 (54.01 to 110.55) | 3822 (2547 to 5506) | 75.39 (50.45 to 108.17) | -0.16 (-0.18 to -0.15) |
| Senegal | 1515 (1067 to 2098) | 56.74 (39.59 to 79.15) | 2328 (1595 to 3297) | 53.19 (36.37 to 75.46) | -0.19 (-0.22 to -0.16) |
| Serbia | 6959 (4871 to 9525) | 497.68 (349.37 to 678.78) | 3717 (2580 to 5141) | 474.1 (330.79 to 651.92) | -0.91 (-1.43 to -0.39) |
| Seychelles | 21 (14 to 30) | 130.15 (88.54 to 185.09) | 20 (14 to 29) | 129.76 (88.42 to 184.68) | 0.02 (0 to 0.03) |
| Sierra Leone | 800 (572 to 1085) | 58.84 (41.49 to 80.75) | 1383 (958 to 1926) | 54.94 (37.87 to 76.83) | -0.22 (-0.25 to -0.19) |
| Singapore | 1613 (1103 to 2277) | 381.08 (261.79 to 535.44) | 2228 (1531 to 3123) | 384.24 (264.86 to 536.91) | 0.08 (0.05 to 0.11) |
| Slovakia | 4086 (2832 to 5649) | 481.36 (334.74 to 662.73) | 2722 (1885 to 3751) | 481.37 (333.56 to 662.87) | 0.09 (-0.3 to 0.48) |
| Slovenia | 1069 (742 to 1482) | 409.58 (285.83 to 565.13) | 832 (578 to 1150) | 408.53 (284.79 to 562.96) | -0.49 (-1.14 to 0.16) |
| Solomon Islands | 144 (97 to 204) | 131.08 (87.87 to 186.46) | 238 (160 to 338) | 131.74 (88.31 to 187.36) | 0.02 (0.01 to 0.03) |
| Somalia | 1275 (899 to 1737) | 45.42 (31.82 to 62.36) | 3070 (2156 to 4270) | 40.88 (28.54 to 57.2) | -0.25 (-0.34 to -0.17) |
| South Africa | 13365 (8937 to 19061) | 142.55 (95.16 to 203.58) | 14499 (9639 to 20804) | 142.3 (94.95 to 203.61) | 0 (-0.02 to 0.02) |
| South Sudan | 941 (678 to 1269) | 49.96 (35.82 to 67.77) | 1444 (1047 to 1952) | 49.08 (35.49 to 66.57) | 0.12 (-0.03 to 0.28) |
| Spain | 6179 (4480 to 8348) | 134.56 (98.31 to 180.36) | 5348 (4155 to 6641) | 132.97 (103.93 to 164) | -0.11 (-0.15 to -0.06) |
| Sri Lanka | 4886 (3345 to 6907) | 133.21 (91.58 to 187.46) | 4273 (2907 to 6120) | 130.21 (88.98 to 185.48) | -0.1 (-0.12 to -0.07) |
| Sudan | 5559 (3810 to 7720) | 87.5 (59.76 to 122.11) | 9479 (6599 to 13200) | 84.26 (58.75 to 117.18) | -0.08 (-0.09 to -0.06) |
| Suriname | 84 (59 to 113) | 93.86 (66.32 to 126.87) | 87 (62 to 119) | 93.84 (66.8 to 127.61) | 0.02 (-0.01 to 0.04) |
| Sweden | 1903 (1351 to 2577) | 180.94 (127.8 to 246.13) | 1453 (1040 to 1983) | 121.65 (87.32 to 165.54) | -1.47 (-1.59 to -1.35) |
| Switzerland | 1305 (946 to 1777) | 166.97 (121.02 to 227.28) | 1441 (1036 to 1983) | 161.59 (116.42 to 221.86) | 0.29 (0.05 to 0.54) |
| Syrian Arab Republic | 3437 (2372 to 4832) | 82.45 (56.84 to 116.04) | 1701 (1158 to 2397) | 80.36 (54.91 to 112.73) | -0.02 (-0.07 to 0.02) |
| Taiwan (Province of China) | 5462 (3731 to 7763) | 155.94 (107.43 to 219.91) | 3108 (2128 to 4406) | 158.11 (109.38 to 222.05) | 0.07 (0.05 to 0.09) |
| Tajikistan | 6527 (4539 to 8950) | 382.62 (264.13 to 528.55) | 9666 (6735 to 13358) | 378.64 (263.16 to 524.43) | -0.05 (-0.06 to -0.03) |
| Thailand | 14604 (10061 to 20446) | 135 (93.36 to 188.2) | 8134 (5542 to 11513) | 133.69 (91.67 to 187.81) | -0.04 (-0.06 to -0.03) |
| Timor-Leste | 336 (232 to 466) | 133.85 (91.72 to 187.38) | 463 (316 to 650) | 130.47 (89.01 to 183.77) | -0.1 (-0.12 to -0.08) |
| Togo | 697 (485 to 972) | 54.5 (37.76 to 76.43) | 1214 (825 to 1717) | 52.77 (35.85 to 74.65) | -0.09 (-0.11 to -0.08) |
| Tokelau | 1 (0 to 1) | 133.44 (89.91 to 189.87) | 0 (0 to 1) | 176.58 (121.46 to 246.67) | 0.31 (0.07 to 0.54) |
| Tonga | 39 (27 to 55) | 134.55 (91.83 to 190.27) | 37 (26 to 53) | 136.64 (93.51 to 192.97) | 0.04 (0.02 to 0.05) |
| Trinidad and Tobago | 261 (185 to 356) | 93.42 (66.43 to 126.83) | 161 (114 to 221) | 92.04 (65.37 to 125.13) | 0 (-0.02 to 0.02) |
| Tunisia | 1834 (1291 to 2525) | 86.3 (60.82 to 118.7) | 1533 (1053 to 2164) | 82.12 (56.67 to 115.38) | -0.13 (-0.15 to -0.12) |
| Turkey | 17248 (12139 to 23777) | 125.87 (88.62 to 173.45) | 14944 (10259 to 20973) | 122.8 (85.2 to 171.01) | -0.26 (-0.38 to -0.14) |
| Turkmenistan | 4105 (2865 to 5660) | 380.48 (264.09 to 527.44) | 3966 (2741 to 5505) | 375.96 (259.79 to 522.08) | -0.02 (-0.03 to -0.01) |
| Tuvalu | 3 (2 to 5) | 131.71 (89.14 to 186.33) | 3 (2 to 5) | 131.96 (89.29 to 187.73) | 0.01 (0 to 0.01) |
| Uganda | 3224 (2441 to 4285) | 51.11 (38.26 to 68.72) | 6943 (4949 to 9635) | 49.81 (35.46 to 69.23) | -0.01 (-0.07 to 0.05) |
| Ukraine | 35187 (23994 to 48754) | 459.97 (314.62 to 635.86) | 17517 (11635 to 24665) | 451.53 (305.46 to 627.49) | -0.05 (-0.06 to -0.04) |
| United Arab Emirates | 345 (237 to 487) | 77.76 (53.32 to 109.98) | 714 (473 to 1030) | 76.53 (51.08 to 109.75) | 0.02 (-0.02 to 0.05) |
| United Kingdom | 10113 (7265 to 13667) | 135.27 (97.12 to 182.93) | 7436 (5486 to 9796) | 97.45 (72.16 to 127.83) | -0.5 (-0.75 to -0.24) |
| United Republic of Tanzania | 4537 (3462 to 5892) | 51.81 (39.22 to 67.77) | 8309 (6108 to 11337) | 48.77 (35.8 to 66.64) | -0.02 (-0.1 to 0.06) |
| United States of America | 83368 (57875 to 115459) | 217.61 (150.91 to 301.68) | 107307 (75702 to 148783) | 279.65 (198 to 386.3) | 0.92 (0.3 to 1.55) |
| United States Virgin Islands | 19 (14 to 26) | 90.82 (63.74 to 124.22) | 7 (5 to 10) | 88.23 (61.6 to 121.65) | -0.01 (-0.05 to 0.02) |
| Uruguay | 952 (646 to 1349) | 174.9 (118.89 to 247.49) | 752 (505 to 1075) | 172.75 (117.31 to 244.85) | -0.06 (-0.07 to -0.04) |
| Uzbekistan | 23363 (16268 to 32235) | 377.38 (261.25 to 523.54) | 27236 (18993 to 37633) | 377.3 (262.15 to 523.06) | 0.01 (0 to 0.01) |
| Vanuatu | 64 (43 to 91) | 130.29 (87.26 to 185.87) | 108 (73 to 153) | 131.49 (88.54 to 187.24) | 0.03 (0.02 to 0.04) |
| Venezuela (Bolivarian Republic of) | 5775 (4136 to 7891) | 118.47 (84.74 to 162.08) | 5209 (3719 to 7153) | 118.54 (84.76 to 162.48) | 0.05 (0.02 to 0.07) |
| Viet Nam | 24496 (16784 to 34471) | 132.37 (90.72 to 186.23) | 22248 (15120 to 31489) | 132 (90.11 to 185.93) | -0.01 (-0.01 to 0) |
| Yemen | 4196 (2876 to 5906) | 81.34 (55.52 to 114.96) | 7463 (5137 to 10473) | 79.6 (54.87 to 111.56) | -0.04 (-0.06 to -0.02) |
| Zambia | 1439 (1091 to 1870) | 52.87 (39.66 to 69.38) | 2652 (1926 to 3657) | 46.25 (33.59 to 63.78) | -0.37 (-0.42 to -0.32) |
| Zimbabwe | 4402 (2971 to 6267) | 130.66 (87.94 to 186.39) | 5804 (3974 to 8231) | 133.89 (91.69 to 189.9) | 0.11 (0.09 to 0.13) |

EAPC, estimated annual percentage change, SDl, Sociodemographic Index; Ul, uncertainty interval. EAPC is expressed as 95% CIs.

**Supplementary Table 2** The prevalence cases and age-standardized prevalence rate of Urogenital Congenital Anomalies in Children Under 9 Years in 1990 and 2021 across 204 countries worldwide, along with their temporal trend.

|  | Rate per 100 000(95%UI) | | 2021 | | 1990-2021 |
| --- | --- | --- | --- | --- | --- |
|  | 1990 | |  | |  |
|  | Prevalence cases | The age-standardized prevalence rate | Prevalence cases | The age-standardized prevalence rate | EAPC |

| Afghanistan | 3936 (2581 to 5542) | 131.86 (87.03 to 186.14) | 12861 (9443 to 17424) | 127.39 (93.4 to 173.17) | 0.03 (-0.06 to 0.11) |
| --- | --- | --- | --- | --- | --- |
| Albania | 4733 (3515 to 6229) | 609.51 (452.38 to 802.79) | 1860 (1385 to 2436) | 639.2 (476.97 to 835.47) | 0.2 (0.12 to 0.28) |
| Algeria | 11306 (8665 to 14956) | 150.74 (115.63 to 199.25) | 13959 (10520 to 18724) | 149.05 (112.37 to 199.83) | 0.03 (-0.03 to 0.09) |
| American Samoa | 24 (17 to 33) | 178.49 (127.18 to 249.62) | 17 (12 to 24) | 195.24 (139.49 to 271.05) | 0.3 (0.29 to 0.32) |
| Andorra | 26 (18 to 38) | 461.76 (310.25 to 665.25) | 18 (13 to 23) | 304.68 (233.28 to 391.88) | -1.12 (-1.37 to -0.87) |
| Angola | 3678 (2449 to 5186) | 104.93 (70.08 to 147.92) | 8570 (6317 to 11624) | 78.99 (58.15 to 107.27) | -0.93 (-0.96 to -0.89) |
| Antigua and Barbuda | 17 (13 to 23) | 141.85 (109.28 to 184.57) | 18 (14 to 22) | 162.47 (128.24 to 206.72) | 0.69 (0.6 to 0.78) |
| Argentina | 16451 (12522 to 22031) | 240.07 (182.86 to 321.25) | 16520 (12368 to 22091) | 244.86 (184.44 to 325.45) | -0.03 (-0.13 to 0.07) |
| Armenia | 2919 (2152 to 3950) | 396.95 (292.33 to 537.62) | 1820 (1363 to 2390) | 459.89 (344.89 to 602.16) | 0.8 (0.66 to 0.95) |
| Australia | 4561 (3840 to 5468) | 180.5 (152.09 to 216.14) | 5455 (4463 to 6628) | 175.49 (143.72 to 212.98) | 0.28 (0.07 to 0.49) |
| Austria | 4516 (4150 to 4909) | 496.25 (455.99 to 539.6) | 4042 (3568 to 4543) | 466.06 (411.47 to 523.87) | -0.14 (-0.32 to 0.04) |
| Azerbaijan | 8016 (5826 to 10770) | 468.94 (340.23 to 631.02) | 7557 (5547 to 10189) | 480.53 (353.96 to 645.63) | 0.07 (0.04 to 0.1) |
| Bahamas | 82 (64 to 105) | 151.91 (118.19 to 193.84) | 74 (57 to 96) | 153.58 (119.75 to 199.05) | 0.08 (-0.04 to 0.2) |
| Bahrain | 149 (113 to 200) | 127.28 (96.15 to 170.56) | 273 (206 to 363) | 137 (103.86 to 181.58) | 0.43 (0.34 to 0.52) |
| Bangladesh | 69357 (49096 to 93394) | 196.71 (139.29 to 265.4) | 57016 (42286 to 77407) | 188.79 (140.39 to 255.59) | -0.1 (-0.14 to -0.05) |
| Barbados | 72 (58 to 90) | 176.72 (142.74 to 221.23) | 53 (41 to 68) | 180.71 (141 to 231.98) | 0.26 (0.15 to 0.38) |
| Belarus | 8431 (6121 to 11407) | 512.91 (373.08 to 692.64) | 5614 (4099 to 7591) | 530.28 (389.2 to 713.41) | 0.23 (0.15 to 0.3) |
| Belgium | 3673 (2982 to 4577) | 307.4 (249.76 to 382.8) | 3806 (3044 to 4700) | 305.35 (244.27 to 376.72) | -0.49 (-0.96 to -0.02) |
| Belize | 94 (75 to 118) | 163.41 (130.21 to 205.66) | 114 (88 to 149) | 143.61 (111.34 to 186.95) | -0.29 (-0.37 to -0.21) |
| Benin | 2030 (1517 to 2645) | 110.64 (82.48 to 144.73) | 4485 (3280 to 6036) | 102.24 (74.72 to 137.74) | -0.21 (-0.26 to -0.15) |
| Bermuda | 14 (11 to 18) | 166.83 (130.48 to 214.26) | 12 (10 to 15) | 231.64 (182.8 to 291.71) | 1.37 (1.18 to 1.55) |
| Bhutan | 341 (243 to 466) | 189.06 (134.43 to 258.55) | 238 (175 to 322) | 194.3 (142.44 to 262.44) | 0.01 (-0.06 to 0.08) |
| Bolivia (Plurinational State of) | 3416 (2630 to 4328) | 179.77 (138.38 to 228.28) | 3981 (3094 to 5143) | 167.91 (130.57 to 216.85) | -0.23 (-0.26 to -0.2) |
| Bosnia and Herzegovina | 4133 (2967 to 5561) | 570.34 (409.98 to 766.23) | 1823 (1307 to 2451) | 572.02 (410.64 to 766.92) | 0.05 (0.01 to 0.08) |
| Botswana | 866 (616 to 1221) | 212.67 (151.1 to 300.1) | 1080 (775 to 1486) | 229.31 (164.54 to 315.23) | 0.69 (0.19 to 1.2) |
| Brazil | 47878 (39589 to 58040) | 138.55 (114.79 to 167.5) | 32923 (26601 to 41064) | 101 (81.61 to 125.96) | -0.84 (-1.01 to -0.66) |
| Brunei Darussalam | 345 (253 to 469) | 539.97 (394.99 to 735.47) | 349 (258 to 476) | 551.67 (408.63 to 749.46) | 0.11 (0.09 to 0.13) |
| Bulgaria | 6570 (4790 to 8760) | 596.55 (435.59 to 793.99) | 3642 (2607 to 4874) | 578.43 (414.58 to 772.37) | -0.11 (-0.13 to -0.08) |
| Burkina Faso | 3891 (2847 to 5120) | 111.41 (81.33 to 147.23) | 7772 (5698 to 10231) | 102.97 (75.39 to 136.06) | -0.09 (-0.17 to -0.01) |
| Burundi | 2387 (1873 to 3004) | 120.15 (94.15 to 151.54) | 3248 (2237 to 4791) | 78.82 (54.38 to 115.93) | -0.9 (-1.11 to -0.69) |
| Cabo Verde | 94 (68 to 131) | 83.59 (60.13 to 115.77) | 105 (78 to 141) | 113.19 (83.76 to 152.71) | 0.99 (0.88 to 1.1) |
| Cambodia | 6236 (4476 to 8421) | 185.67 (133.04 to 251.64) | 6272 (4545 to 8527) | 180.81 (131.1 to 245.64) | -0.14 (-0.19 to -0.1) |
| Cameroon | 3708 (2753 to 4834) | 102.34 (75.66 to 134.17) | 9272 (6855 to 12223) | 98.49 (72.78 to 129.93) | 0.04 (-0.04 to 0.13) |
| Canada | 16905 (13250 to 22055) | 436.88 (342.88 to 568.97) | 16365 (12620 to 21677) | 404.02 (312.75 to 532.67) | -0.08 (-0.16 to 0.01) |
| Central African Republic | 824 (569 to 1125) | 91.19 (63.01 to 124.98) | 1289 (926 to 1738) | 81.16 (58.2 to 109.63) | -0.35 (-0.39 to -0.31) |
| Chad | 2123 (1546 to 2794) | 96.48 (69.76 to 127.97) | 6307 (4601 to 8270) | 95.28 (69.27 to 125.5) | 0.07 (0.03 to 0.1) |
| Chile | 7445 (5452 to 10311) | 270.5 (197.88 to 375.16) | 6799 (6127 to 7546) | 280.72 (253.03 to 311.75) | -0.92 (-1.26 to -0.57) |
| China | 693696 (524688 to 915519) | 321.1 (242.56 to 424.45) | 573827 (431804 to 755905) | 334.12 (251.14 to 439.72) | 0.27 (0.17 to 0.38) |
| Colombia | 16549 (13044 to 21054) | 206.75 (162.8 to 263.33) | 16077 (12699 to 20436) | 230.55 (182.12 to 292.93) | 0.97 (0.78 to 1.16) |
| Comoros | 169 (126 to 222) | 109.79 (81.69 to 144.27) | 172 (126 to 229) | 106.36 (77.94 to 141.95) | -0.13 (-0.32 to 0.06) |
| Congo | 647 (475 to 868) | 87.45 (64.06 to 117.66) | 980 (710 to 1339) | 75.54 (54.86 to 103) | -0.51 (-0.55 to -0.47) |
| Cook Islands | 8 (6 to 11) | 177.24 (124.61 to 248.8) | 5 (3 to 7) | 210.71 (145.63 to 312.22) | 0.13 (-0.02 to 0.27) |
| Costa Rica | 1709 (1371 to 2151) | 217.8 (174.68 to 274.4) | 1480 (1162 to 1883) | 223.74 (176.03 to 283.67) | 0.26 (0.2 to 0.32) |
| Cote d’Ivoire | 4023 (2944 to 5456) | 95.19 (69.46 to 129.56) | 7545 (5303 to 10615) | 91.82 (64.51 to 129.12) | -0.06 (-0.17 to 0.05) |
| Croatia | 3964 (2925 to 5295) | 618.15 (456.81 to 823.73) | 2389 (1916 to 2936) | 628.71 (504.54 to 772.33) | 0.59 (0.36 to 0.82) |
| Cuba | 2971 (2408 to 3715) | 176.1 (142.49 to 220.62) | 2056 (1618 to 2598) | 175.18 (137.96 to 220.86) | 0.2 (0.11 to 0.3) |
| Cyprus | 225 (176 to 289) | 169.78 (132.53 to 218) | 279 (208 to 365) | 188.38 (140.7 to 246.88) | 1.33 (0.84 to 1.82) |
| Czechia | 7252 (5328 to 9653) | 538.91 (396.54 to 715.8) | 6059 (4442 to 8041) | 538.56 (394.99 to 714.22) | -0.51 (-0.87 to -0.15) |
| Democratic People's Republic of Korea | 10541 (7759 to 14051) | 251.97 (184.42 to 337.98) | 7611 (5407 to 10497) | 245.69 (174.89 to 338.24) | 0 (-0.05 to 0.06) |
| Democratic Republic of the Congo | 11134 (7857 to 14882) | 85.62 (60.2 to 115.12) | 18704 (13479 to 25574) | 70.87 (51.05 to 96.95) | -0.52 (-0.57 to -0.46) |
| Denmark | 1505 (1209 to 1885) | 268.87 (215.93 to 337.1) | 1748 (1397 to 2191) | 283.13 (226.3 to 354.79) | 0.22 (0.09 to 0.36) |
| Djibouti | 135 (101 to 178) | 110.39 (82.94 to 145.35) | 271 (186 to 381) | 95.34 (65.33 to 133.78) | -0.35 (-0.55 to -0.15) |
| Dominica | 25 (19 to 32) | 147.43 (114.13 to 189.83) | 17 (14 to 22) | 211.69 (168.24 to 265.32) | 1.26 (1.18 to 1.34) |
| Dominican Republic | 3153 (2476 to 4029) | 169.47 (132.83 to 217.05) | 3279 (2562 to 4204) | 163.48 (127.75 to 209.65) | -0.13 (-0.21 to -0.04) |
| Ecuador | 3587 (3106 to 4147) | 135.74 (117.54 to 156.94) | 5121 (4518 to 5877) | 151.38 (133.39 to 173.99) | 0.66 (0.56 to 0.75) |
| Egypt | 21873 (16407 to 29404) | 140.75 (105.29 to 189.95) | 36580 (27786 to 47969) | 141.9 (107.84 to 186.01) | 0.19 (0.06 to 0.32) |
| El Salvador | 2795 (2173 to 3640) | 190.69 (148.12 to 248.6) | 2351 (1790 to 3062) | 189.57 (144.5 to 246.61) | 0.02 (-0.02 to 0.06) |
| Equatorial Guinea | 120 (84 to 164) | 82.96 (57.89 to 113.76) | 304 (215 to 419) | 78.21 (55.51 to 107.55) | -0.3 (-0.38 to -0.22) |
| Eritrea | 1199 (873 to 1594) | 103.08 (75.21 to 137.08) | 1739 (1259 to 2380) | 98.2 (71.1 to 134.33) | -0.17 (-0.29 to -0.05) |
| Estonia | 1430 (1078 to 1861) | 601.22 (453.17 to 782.21) | 965 (715 to 1276) | 687.35 (510.17 to 909.04) | 0.6 (0.37 to 0.83) |
| Eswatini | 594 (425 to 825) | 219.4 (156.79 to 305.65) | 628 (454 to 870) | 224.03 (162.3 to 310.33) | 0.08 (0.04 to 0.12) |
| Ethiopia | 23730 (14425 to 32379) | 132.06 (80.96 to 179.86) | 31468 (23756 to 42109) | 102.88 (77.64 to 137.64) | -0.96 (-1.19 to -0.73) |
| Fiji | 351 (247 to 494) | 181.37 (127.7 to 255.02) | 369 (267 to 508) | 199.29 (144.66 to 274.14) | 0.28 (0.25 to 0.32) |
| Finland | 1612 (1241 to 2063) | 251.15 (193.68 to 321.04) | 1621 (1295 to 2014) | 300.48 (240.44 to 371.94) | 0.28 (0.14 to 0.43) |
| France | 20826 (16919 to 25964) | 265.19 (215.81 to 330.17) | 22458 (18278 to 27806) | 303.78 (247.72 to 374.97) | 0.61 (0.53 to 0.69) |
| Gabon | 233 (169 to 313) | 80.57 (58.12 to 108.76) | 347 (251 to 472) | 80.15 (58 to 108.76) | 0.12 (0.06 to 0.19) |
| Gambia | 412 (293 to 546) | 119.83 (85.36 to 159.5) | 709 (507 to 971) | 103.05 (73.66 to 141.15) | -0.61 (-0.75 to -0.47) |
| Georgia | 3387 (2515 to 4522) | 364.96 (271.06 to 486.95) | 1306 (1057 to 1602) | 258.9 (209.88 to 317.19) | -1.41 (-1.59 to -1.22) |
| Germany | 25857 (20346 to 32963) | 294.38 (231.64 to 375.29) | 25121 (19647 to 31751) | 310.85 (243.27 to 392.49) | 0.48 (0.2 to 0.76) |
| Ghana | 5146 (3809 to 6729) | 105.57 (78.01 to 138.44) | 7744 (5632 to 10557) | 86.09 (62.58 to 117.41) | -0.88 (-1.07 to -0.69) |
| Greece | 4034 (3230 to 5087) | 323.03 (259.35 to 405.43) | 2502 (2086 to 2978) | 287.76 (240.14 to 342.12) | -0.44 (-0.64 to -0.24) |
| Greenland | 35 (26 to 48) | 344.78 (250.28 to 473.01) | 26 (18 to 36) | 323.72 (231.58 to 450.82) | -0.16 (-0.2 to -0.12) |
| Grenada | 38 (30 to 48) | 163.2 (129.61 to 207.26) | 24 (19 to 31) | 171.55 (137.69 to 218.66) | 0.31 (0.22 to 0.4) |
| Guam | 58 (42 to 78) | 198.18 (144.63 to 269.74) | 53 (39 to 73) | 217.29 (157.6 to 298.94) | 0.69 (0.56 to 0.81) |
| Guatemala | 5718 (4428 to 7350) | 197.65 (152.87 to 254.48) | 6216 (4764 to 8150) | 187.03 (143.67 to 244.5) | -0.06 (-0.1 to -0.02) |
| Guinea | 3168 (2217 to 4173) | 148.56 (104.1 to 196.38) | 4659 (3210 to 6610) | 107.98 (74.47 to 153.15) | -0.81 (-0.89 to -0.72) |
| Guinea-Bissau | 397 (290 to 530) | 112.34 (82 to 150.37) | 534 (387 to 729) | 84.27 (60.95 to 115.22) | -0.82 (-0.96 to -0.68) |
| Guyana | 269 (205 to 348) | 134.95 (102.9 to 175.94) | 211 (164 to 275) | 145.03 (112.73 to 188.46) | 0.45 (0.33 to 0.57) |
| Haiti | 3928 (2701 to 5130) | 200.73 (138.9 to 262.28) | 5424 (3945 to 7132) | 179.99 (131.01 to 236.75) | -0.2 (-0.28 to -0.13) |
| Honduras | 2974 (2296 to 3840) | 190.99 (147.3 to 246.82) | 3872 (2904 to 5104) | 176.18 (132.2 to 232.1) | -0.29 (-0.31 to -0.27) |
| Hungary | 7555 (5476 to 10073) | 589.88 (428.13 to 784.98) | 5398 (3900 to 7178) | 590.84 (427.07 to 784.98) | 0.01 (-0.03 to 0.05) |
| Iceland | 129 (106 to 158) | 303.67 (249.58 to 370.38) | 109 (84 to 141) | 248.92 (193.59 to 321.7) | -0.11 (-0.4 to 0.19) |
| India | 613063 (450710 to 839031) | 266.23 (195.83 to 364.17) | 820447 (590067 to 1141870) | 346.18 (249.91 to 479.97) | 1.25 (1 to 1.49) |
| Indonesia | 123651 (86439 to 174123) | 269.79 (188.92 to 378.92) | 136366 (100623 to 181861) | 305.11 (225.31 to 406.24) | 0.56 (0.45 to 0.68) |
| Iran (Islamic Republic of) | 30263 (23463 to 39025) | 166.89 (129.54 to 214.79) | 23316 (16855 to 31424) | 169.28 (121.52 to 229.12) | 0.67 (0.41 to 0.93) |
| Iraq | 8759 (6604 to 11521) | 150 (113.01 to 197.72) | 16044 (11990 to 21398) | 179.8 (134.45 to 239.61) | 0.88 (0.77 to 0.98) |
| Ireland | 1618 (1280 to 2041) | 258.39 (204.99 to 324.72) | 1571 (1236 to 2010) | 245.26 (193.47 to 312.63) | 0.3 (0.14 to 0.46) |
| Israel | 2780 (2225 to 3469) | 269.16 (215.61 to 335.56) | 4396 (3442 to 5582) | 242.68 (190.06 to 308.07) | 0.04 (-0.14 to 0.22) |
| Italy | 24009 (19255 to 30075) | 423.15 (340.05 to 528.71) | 20169 (16791 to 23905) | 425.1 (354.39 to 502.89) | -0.17 (-0.4 to 0.05) |
| Jamaica | 1027 (834 to 1295) | 182.49 (148.31 to 229.73) | 617 (484 to 796) | 167.11 (131.11 to 215.22) | -0.24 (-0.31 to -0.16) |
| Japan | 99715 (76035 to 133486) | 686.31 (526.05 to 912.81) | 65054 (51607 to 82500) | 651.41 (518.71 to 822.33) | -0.26 (-0.33 to -0.19) |
| Jordan | 1243 (1001 to 1542) | 108.94 (87.64 to 135.25) | 3486 (2779 to 4385) | 149.98 (119.22 to 189.01) | 1.26 (1.12 to 1.41) |
| Kazakhstan | 16769 (12212 to 22519) | 467.28 (339.84 to 628.29) | 16761 (11896 to 22825) | 443.89 (314.86 to 604.8) | -0.12 (-0.17 to -0.08) |
| Kenya | 8033 (5951 to 10518) | 99.44 (73.62 to 130.52) | 10938 (8146 to 14722) | 89.89 (66.95 to 120.9) | -0.04 (-0.22 to 0.15) |
| Kiribati | 38 (27 to 53) | 176.82 (124.23 to 248.3) | 51 (35 to 72) | 176.9 (123.3 to 250.85) | -0.03 (-0.05 to -0.01) |
| Kuwait | 817 (659 to 1024) | 208.22 (167.89 to 261) | 1115 (865 to 1427) | 198.5 (154.09 to 253.82) | 0.39 (0.16 to 0.61) |
| Kyrgyzstan | 9150 (6528 to 12609) | 770.65 (548.48 to 1065.5) | 12173 (8673 to 16879) | 763.54 (544.4 to 1057.54) | 0.01 (-0.01 to 0.03) |
| Lao People's Democratic Republic | 2435 (1682 to 3319) | 185.45 (128.06 to 253.44) | 2811 (2035 to 3857) | 177.6 (128.45 to 244.07) | -0.15 (-0.17 to -0.14) |
| Latvia | 1865 (1372 to 2545) | 471.16 (346.52 to 642.97) | 894 (644 to 1236) | 451.54 (326.13 to 622.15) | -0.34 (-0.49 to -0.19) |
| Lebanon | 1048 (803 to 1381) | 143.77 (110.02 to 190.04) | 1599 (1115 to 2280) | 188.35 (130.84 to 270.09) | 0.98 (0.83 to 1.12) |
| Lesotho | 994 (711 to 1403) | 209.48 (149.69 to 295.9) | 922 (663 to 1290) | 218.44 (157.35 to 304.9) | 0.18 (0.16 to 0.2) |
| Liberia | 1030 (708 to 1389) | 120.59 (83.14 to 162.92) | 1368 (1008 to 1841) | 91.71 (67.57 to 123.43) | -0.93 (-1.26 to -0.6) |
| Libya | 2070 (1585 to 2706) | 167.76 (128.37 to 219.41) | 2127 (1549 to 2831) | 232.01 (168.38 to 309.4) | 1.47 (1.28 to 1.66) |
| Lithuania | 3096 (2271 to 4151) | 545.71 (400.29 to 731.65) | 1475 (1066 to 1986) | 536.11 (388.21 to 720.01) | -0.63 (-1.02 to -0.24) |
| Luxembourg | 126 (100 to 160) | 279.96 (223.01 to 355.49) | 181 (143 to 232) | 270.15 (213.95 to 345.85) | -0.48 (-0.99 to 0.03) |
| Madagascar | 4216 (3233 to 5408) | 104.99 (80.3 to 135.05) | 6751 (4961 to 9276) | 83.67 (61.48 to 114.97) | -0.54 (-0.65 to -0.42) |
| Malawi | 7316 (5631 to 9166) | 207.58 (159.69 to 260.53) | 8152 (4725 to 13206) | 151.21 (87.45 to 245.28) | -0.81 (-0.94 to -0.67) |
| Malaysia | 7991 (5769 to 10991) | 174.48 (125.85 to 240.2) | 9115 (6598 to 12370) | 180.82 (131.16 to 244.85) | 0.2 (0.14 to 0.27) |
| Maldives | 138 (98 to 188) | 180.45 (128.08 to 246.91) | 140 (104 to 189) | 209.7 (156.17 to 281.86) | 0.65 (0.58 to 0.72) |
| Mali | 4245 (2904 to 5678) | 134.05 (91.96 to 179.84) | 9116 (6317 to 12856) | 107.7 (74.75 to 151.8) | -0.61 (-0.68 to -0.55) |
| Malta | 144 (113 to 179) | 249.3 (195.67 to 310) | 147 (114 to 189) | 337.68 (261.46 to 435.09) | 0.67 (0.31 to 1.04) |
| Marshall Islands | 27 (18 to 38) | 171.08 (118.07 to 242.52) | 21 (15 to 29) | 179.19 (125.32 to 251.59) | 0.15 (0.13 to 0.17) |
| Mauritania | 649 (468 to 920) | 95.72 (68.88 to 135.89) | 1207 (873 to 1624) | 93.73 (67.78 to 126.1) | -0.18 (-0.27 to -0.09) |
| Mauritius | 365 (261 to 500) | 171.09 (122.49 to 234.15) | 231 (167 to 315) | 175.15 (126.92 to 238.51) | -0.01 (-0.16 to 0.13) |
| Mexico | 53230 (42626 to 68378) | 234.96 (187.99 to 302.18) | 45741 (36414 to 58001) | 217.74 (173.64 to 275.36) | -0.28 (-0.37 to -0.19) |
| Micronesia (Federated States of) | 56 (39 to 79) | 176.13 (123.63 to 248.2) | 36 (25 to 50) | 178.46 (125.85 to 251.14) | 0.06 (0.04 to 0.07) |
| Monaco | 12 (8 to 18) | 513.73 (329.59 to 770.51) | 18 (13 to 25) | 560.74 (381.3 to 766.06) | -0.88 (-1.35 to -0.41) |
| Mongolia | 3162 (2301 to 4206) | 499.8 (362.78 to 667.04) | 3511 (2512 to 4806) | 448.21 (321.13 to 613) | -0.36 (-0.41 to -0.31) |
| Montenegro | 685 (513 to 892) | 637.17 (477.82 to 828.57) | 422 (304 to 564) | 574.83 (414.15 to 768.41) | -0.22 (-0.26 to -0.18) |
| Morocco | 8476 (6269 to 11439) | 125.34 (92.56 to 169.52) | 8358 (6092 to 11578) | 127.31 (92.93 to 176.17) | 0.17 (0.12 to 0.22) |
| Mozambique | 5450 (3996 to 7290) | 119.29 (87.44 to 159.67) | 8957 (6252 to 13214) | 88.78 (61.98 to 130.92) | -0.74 (-0.87 to -0.61) |
| Myanmar | 19404 (13358 to 26800) | 193.73 (133.35 to 267.44) | 19894 (14510 to 27179) | 190.68 (139.18 to 260.26) | -0.09 (-0.12 to -0.06) |
| Namibia | 928 (674 to 1281) | 223.17 (161.61 to 309.15) | 1386 (1022 to 1898) | 249.09 (183.91 to 340.83) | 0.44 (0.36 to 0.52) |
| Nauru | 6 (4 to 8) | 184.52 (131.6 to 257.76) | 5 (4 to 7) | 195.92 (140.77 to 270.66) | 0.16 (0.14 to 0.18) |
| Nepal | 8398 (6018 to 11257) | 138.95 (99.46 to 186.82) | 10003 (7612 to 13020) | 162.77 (123.89 to 211.8) | 0.25 (-0.23 to 0.72) |
| Netherlands | 5333 (4347 to 6597) | 292.27 (238.17 to 361.61) | 4518 (3612 to 5657) | 258.59 (206.92 to 323.5) | -0.37 (-0.48 to -0.25) |
| New Zealand | 944 (766 to 1169) | 175.97 (142.69 to 218.09) | 1037 (868 to 1250) | 162.41 (136.01 to 195.21) | 0.13 (-0.09 to 0.36) |
| Nicaragua | 2774 (2190 to 3529) | 215.82 (170.27 to 274.71) | 2586 (1963 to 3395) | 193.92 (147.35 to 254.35) | -0.18 (-0.23 to -0.13) |
| Niger | 3793 (2767 to 4965) | 122.46 (89.21 to 161.11) | 8018 (5842 to 10901) | 86.14 (62.58 to 117.46) | -1.13 (-1.23 to -1.03) |
| Nigeria | 36314 (28852 to 45311) | 126.23 (99.6 to 158.88) | 89912 (60683 to 124353) | 126.4 (85.43 to 174.75) | 0.18 (0.07 to 0.28) |
| Niue | 1 (1 to 2) | 189.46 (135.7 to 265.45) | 1 (1 to 1) | 391.94 (292.36 to 545.81) | 1.07 (0.6 to 1.55) |
| North Macedonia | 2124 (1553 to 2795) | 607.06 (444.43 to 797.14) | 1247 (897 to 1679) | 581.06 (419.06 to 780.19) | -0.01 (-0.06 to 0.03) |
| Northern Mariana Islands | 15 (10 to 20) | 176.18 (123.75 to 248.26) | 13 (9 to 18) | 184.62 (130.98 to 257.24) | 0.19 (0.15 to 0.23) |
| Norway | 1854 (1504 to 2320) | 347.82 (281.95 to 435.62) | 1814 (1418 to 2331) | 303.9 (238.59 to 388.77) | -0.46 (-0.61 to -0.3) |
| Oman | 732 (540 to 989) | 120.87 (88.93 to 164.01) | 1139 (847 to 1547) | 130.06 (96.92 to 176.08) | 0.48 (0.32 to 0.63) |
| Pakistan | 71563 (53830 to 95845) | 205.32 (154.11 to 275.69) | 132510 (101491 to 174666) | 225.74 (172.95 to 297.45) | 0.49 (0.41 to 0.57) |
| Palau | 5 (4 to 8) | 179.9 (126.69 to 250.55) | 4 (3 to 5) | 183.76 (130.28 to 257.18) | 0.05 (0.03 to 0.06) |
| Palestine | 1080 (797 to 1452) | 153.7 (113.52 to 207.24) | 2143 (1654 to 2762) | 170.45 (131.66 to 219.37) | 0.62 (0.47 to 0.76) |
| Panama | 1230 (972 to 1560) | 217.07 (171.52 to 275.14) | 1857 (1466 to 2367) | 239.94 (189.57 to 305.26) | 0.24 (0.19 to 0.28) |
| Papua New Guinea | 2129 (1511 to 2990) | 178.29 (126.1 to 251.35) | 5056 (3623 to 7001) | 182.57 (130.21 to 254.05) | 0.06 (0.03 to 0.08) |
| Paraguay | 1399 (1097 to 1822) | 118.06 (92.56 to 153.89) | 1710 (1297 to 2255) | 129.12 (97.89 to 170.35) | 0.51 (0.37 to 0.64) |
| Peru | 9907 (7877 to 12476) | 174.21 (138.44 to 219.51) | 11661 (8686 to 15321) | 179.32 (133.55 to 235.59) | 0.41 (0.3 to 0.52) |
| Philippines | 30703 (22919 to 40345) | 175.35 (130.73 to 230.87) | 26852 (20965 to 34834) | 118.56 (92.67 to 153.63) | -1.6 (-1.86 to -1.34) |
| Poland | 67872 (47957 to 93981) | 1061.3 (752.69 to 1462.6) | 41909 (33347 to 51640) | 1095.94 (872.11 to 1349.32) | 0.29 (-0.28 to 0.87) |
| Portugal | 3393 (2812 to 4187) | 267.43 (222.19 to 328.61) | 2521 (2077 to 3053) | 294.2 (242.46 to 356.02) | 0.28 (0.08 to 0.48) |
| Puerto Rico | 1033 (828 to 1312) | 159.8 (128.21 to 202.58) | 403 (311 to 526) | 157.35 (122.34 to 203.24) | 0.31 (0.18 to 0.43) |
| Qatar | 122 (91 to 166) | 137.75 (102.59 to 188) | 551 (409 to 739) | 157.71 (117.05 to 211.64) | 0.45 (0.35 to 0.55) |
| Republic of Korea | 40410 (29818 to 55295) | 542.41 (401.79 to 737.62) | 23176 (17153 to 31652) | 601.41 (448.35 to 813.25) | 0.46 (0.4 to 0.52) |
| Republic of Moldova | 4921 (3735 to 6519) | 576.92 (438.11 to 763.99) | 1775 (1302 to 2389) | 525.69 (387.45 to 703.92) | -0.25 (-0.37 to -0.13) |
| Romania | 24316 (17991 to 32390) | 691.58 (511.88 to 920.67) | 12831 (9407 to 17208) | 666.31 (489.12 to 891.8) | -0.28 (-0.38 to -0.18) |
| Russian Federation | 145587 (108221 to 194126) | 613.69 (457.17 to 816.78) | 98495 (70765 to 134587) | 567.07 (410.99 to 769.59) | -0.31 (-0.41 to -0.21) |
| Rwanda | 3302 (2551 to 4181) | 130.79 (101.02 to 165.81) | 3309 (2458 to 4494) | 97.09 (72.15 to 131.85) | -1.06 (-1.26 to -0.85) |
| Saint Kitts and Nevis | 14 (11 to 18) | 145.56 (113 to 187.7) | 10 (8 to 13) | 163.37 (127.41 to 210.11) | 0.51 (0.41 to 0.62) |
| Saint Lucia | 54 (42 to 69) | 154.42 (120.47 to 197.66) | 32 (26 to 41) | 168.48 (134.47 to 213.06) | 0.26 (0.16 to 0.35) |
| Saint Vincent and the Grenadines | 48 (38 to 61) | 175.67 (140.63 to 224.86) | 29 (24 to 37) | 178.58 (144.57 to 224.39) | 0.05 (-0.08 to 0.18) |
| Samoa | 95 (69 to 129) | 196.82 (143.01 to 268.36) | 112 (80 to 154) | 200.32 (143.69 to 275.57) | 0.03 (0 to 0.06) |
| San Marino | 17 (12 to 24) | 698.43 (485.75 to 977.6) | 11 (8 to 15) | 415.34 (301.93 to 566.3) | -1.41 (-1.53 to -1.28) |
| Sao Tome and Principe | 43 (33 to 57) | 108.7 (82.7 to 142.75) | 44 (30 to 62) | 85.04 (58.96 to 120.11) | -0.67 (-0.94 to -0.4) |
| Saudi Arabia | 5932 (4422 to 8085) | 127.3 (94.82 to 173.71) | 6021 (4352 to 8314) | 118.36 (85.78 to 162.97) | -0.25 (-0.32 to -0.18) |
| Senegal | 2889 (2173 to 3755) | 107.02 (80.16 to 139.72) | 3889 (2826 to 5256) | 88.82 (64.5 to 120.14) | -0.56 (-0.66 to -0.46) |
| Serbia | 8531 (6361 to 11234) | 606.08 (452.49 to 796.23) | 4404 (3211 to 5941) | 554.94 (405.39 to 746.3) | -1.09 (-1.63 to -0.54) |
| Seychelles | 28 (20 to 38) | 173.44 (124.28 to 238.14) | 28 (20 to 38) | 178.63 (130.6 to 244.64) | 0.15 (0.1 to 0.21) |
| Sierra Leone | 1673 (1212 to 2202) | 120.39 (87.07 to 159.18) | 2433 (1781 to 3261) | 96.39 (70.43 to 129.44) | -0.75 (-0.83 to -0.67) |
| Singapore | 2461 (1824 to 3318) | 576.08 (428.26 to 773.65) | 3616 (2736 to 4774) | 620.25 (470.15 to 817.19) | 0.37 (0.31 to 0.43) |
| Slovakia | 4828 (3510 to 6523) | 563.92 (410.43 to 760.43) | 3270 (2425 to 4362) | 577.62 (428.56 to 770.25) | 0.19 (-0.22 to 0.6) |
| Slovenia | 1297 (959 to 1716) | 491.84 (364.48 to 648.8) | 1022 (762 to 1351) | 498.33 (372.38 to 656.92) | -0.31 (-0.93 to 0.31) |
| Solomon Islands | 186 (130 to 262) | 170.96 (118.81 to 242.52) | 315 (223 to 443) | 175.21 (123.57 to 246.71) | 0.08 (0.05 to 0.11) |
| Somalia | 2815 (1916 to 3853) | 98.41 (67.18 to 134.77) | 5493 (3742 to 7582) | 72.7 (49.63 to 100.19) | -0.73 (-0.96 to -0.49) |
| South Africa | 21028 (15001 to 29463) | 224.85 (160.23 to 315.43) | 23236 (16673 to 32567) | 226.83 (163.16 to 317.14) | 0.05 (-0.02 to 0.11) |
| South Sudan | 2336 (1624 to 3117) | 121.88 (84.91 to 162.73) | 3525 (2558 to 4628) | 118.89 (86.34 to 156.03) | 0.34 (-0.02 to 0.71) |
| Spain | 12563 (10333 to 15372) | 274.61 (226.3 to 335.08) | 11711 (10047 to 13601) | 289.97 (248.71 to 336.68) | 0 (-0.17 to 0.17) |
| Sri Lanka | 6929 (5162 to 9416) | 187.69 (140.22 to 254.21) | 6076 (4363 to 8337) | 183.81 (132.34 to 251.45) | -0.13 (-0.22 to -0.04) |
| Sudan | 10113 (6775 to 13992) | 158.36 (106.39 to 219.54) | 17501 (13110 to 23370) | 155.64 (116.65 to 207.71) | 0.08 (0.02 to 0.13) |
| Suriname | 139 (109 to 179) | 155.39 (121.56 to 200.21) | 153 (119 to 197) | 162.38 (126.82 to 209.47) | 0.21 (0.14 to 0.28) |
| Sweden | 3814 (3060 to 4749) | 361.3 (289.29 to 451.05) | 2758 (2245 to 3414) | 230.4 (187.78 to 284.84) | -1.5 (-1.66 to -1.34) |
| Switzerland | 2793 (2259 to 3391) | 357.32 (288.97 to 433.88) | 2889 (2337 to 3554) | 323.61 (261.89 to 397.87) | -0.38 (-0.55 to -0.2) |
| Syrian Arab Republic | 5722 (4228 to 7625) | 137.35 (101.46 to 183.12) | 3082 (2264 to 4178) | 145.31 (106.58 to 196.89) | 0.42 (0.28 to 0.57) |
| Taiwan (Province of China) | 8881 (6600 to 11798) | 251.05 (187.68 to 331.09) | 5504 (4100 to 7267) | 279.67 (209.81 to 366.11) | 0.47 (0.37 to 0.57) |
| Tajikistan | 8027 (5896 to 10652) | 474.84 (347.22 to 633.74) | 11769 (8493 to 15961) | 462.73 (333.47 to 628.36) | -0.15 (-0.2 to -0.09) |
| Thailand | 21785 (16284 to 29224) | 200.07 (149.98 to 267.54) | 12917 (9718 to 17112) | 211.22 (159.6 to 278.18) | 0.13 (0.07 to 0.19) |
| Timor-Leste | 452 (309 to 614) | 182.07 (124.37 to 248.7) | 616 (438 to 842) | 174.3 (123.85 to 238.63) | -0.16 (-0.21 to -0.11) |
| Togo | 1207 (914 to 1600) | 94.05 (70.98 to 125.07) | 1967 (1402 to 2691) | 85.51 (60.94 to 116.98) | -0.3 (-0.36 to -0.24) |
| Tokelau | 1 (1 to 1) | 183.3 (129.82 to 256.47) | 1 (1 to 2) | 462.14 (300.08 to 697.71) | 1.07 (0.33 to 1.82) |
| Tonga | 56 (40 to 78) | 196.58 (140.49 to 272.78) | 59 (43 to 82) | 214.57 (156.11 to 300.91) | 0.19 (0.11 to 0.27) |
| Trinidad and Tobago | 466 (373 to 588) | 165.34 (132.68 to 208.18) | 297 (233 to 383) | 168.49 (131.84 to 216.57) | 0.26 (0.18 to 0.33) |
| Tunisia | 3767 (2920 to 4863) | 177.43 (137.59 to 228.91) | 3133 (2353 to 4208) | 168.03 (126.3 to 225.66) | -0.07 (-0.13 to -0.02) |
| Turkey | 33118 (25287 to 43388) | 241.65 (184.54 to 316.53) | 31663 (24024 to 41220) | 260.25 (198.04 to 337.13) | 0.28 (0.19 to 0.38) |
| Turkmenistan | 5004 (3649 to 6687) | 466.97 (339.21 to 626.51) | 4757 (3433 to 6462) | 451.22 (325.56 to 613.07) | -0.06 (-0.09 to -0.03) |
| Tuvalu | 4 (3 to 6) | 177.11 (124.45 to 248.5) | 5 (3 to 6) | 179.27 (126.81 to 251.34) | 0.04 (0.02 to 0.06) |
| Uganda | 8571 (6357 to 11555) | 131.87 (97.83 to 177.47) | 18071 (11933 to 26946) | 128.92 (85.46 to 191.58) | 0.11 (-0.04 to 0.25) |
| Ukraine | 45079 (32904 to 60986) | 587.66 (429.68 to 793.97) | 22214 (15836 to 30752) | 562.49 (404.98 to 772.6) | -0.1 (-0.15 to -0.05) |
| United Arab Emirates | 539 (401 to 726) | 121.73 (90.47 to 163.88) | 1133 (807 to 1581) | 120.89 (86.46 to 168.01) | 0.24 (0.12 to 0.36) |
| United Kingdom | 20302 (17109 to 24606) | 271.46 (228.67 to 329.17) | 14807 (12664 to 17525) | 193.77 (166.01 to 228.75) | -0.48 (-0.69 to -0.28) |
| United Republic of Tanzania | 11697 (9147 to 14595) | 131.27 (102.5 to 164.33) | 20317 (14720 to 28274) | 118.81 (86.14 to 165.17) | 0.06 (-0.13 to 0.24) |
| United States of America | 162851 (129386 to 208986) | 425.45 (337.78 to 546.43) | 190027 (149442 to 240533) | 491.47 (387.3 to 620.86) | 0.66 (0.19 to 1.14) |
| United States Virgin Islands | 32 (25 to 42) | 151.4 (116.34 to 196) | 12 (9 to 16) | 144.65 (109.44 to 193.32) | 0.12 (-0.01 to 0.25) |
| Uruguay | 1411 (1061 to 1884) | 258.79 (194.93 to 345.28) | 1144 (844 to 1548) | 259.53 (192.56 to 348.46) | -0.03 (-0.12 to 0.06) |
| Uzbekistan | 27787 (19879 to 37469) | 452.82 (322.71 to 612.87) | 33100 (24000 to 44840) | 461.17 (333.7 to 625.85) | 0.07 (0.05 to 0.09) |
| Vanuatu | 82 (57 to 116) | 168.8 (117.25 to 239.45) | 142 (99 to 201) | 173.45 (121.09 to 246.08) | 0.1 (0.08 to 0.13) |
| Venezuela (Bolivarian Republic of) | 9714 (7634 to 12459) | 199.93 (156.99 to 256.62) | 9399 (7234 to 12129) | 213.04 (163.94 to 274.78) | 0.34 (0.25 to 0.42) |
| Viet Nam | 35772 (26253 to 48518) | 193.29 (141.89 to 262.11) | 34934 (25312 to 47334) | 206.99 (150.36 to 279.72) | 0.23 (0.2 to 0.26) |
| Yemen | 6654 (4698 to 9185) | 129.08 (91.06 to 178.65) | 11868 (8764 to 16104) | 126.5 (93.5 to 171.46) | 0.06 (-0.01 to 0.13) |
| Zambia | 3862 (3034 to 4802) | 138.26 (108.42 to 172.3) | 6011 (4369 to 8325) | 104.82 (76.19 to 145.17) | -0.77 (-0.88 to -0.65) |
| Zimbabwe | 7405 (5366 to 10360) | 220.62 (159.61 to 309.15) | 10319 (7584 to 14115) | 238.05 (174.97 to 325.64) | 0.33 (0.26 to 0.39) |

EAPC, estimated annual percentage change, SDl, Sociodemographic Index; Ul, uncertainty interval. EAPC is expressed as 95% CIs.

**Supplementary Table 3** The deaths cases and age-standardized deaths rate of Urogenital Congenital Anomalies in Children Under 9 Years in 1990 and 2021 across 204 countries worldwide, along with their temporal trend.

|  | Rate per 100 000(95%UI) | | 2021 | | 1990-2021 |
| --- | --- | --- | --- | --- | --- |
|  | 1990 | |  | |  |
|  | Deaths cases | The age-standardized deaths rate | Deaths cases | The age-standardized deaths rate | EAPC |

| Afghanistan | 265 (98 to 445) | 8.6 (3.28 to 14.36) | 637 (419 to 941) | 6.24 (4.12 to 9.17) | -0.71 (-0.96 to -0.47) |
| --- | --- | --- | --- | --- | --- |
| Albania | 85 (57 to 108) | 10.94 (7.32 to 13.82) | 17 (12 to 23) | 5.92 (4.33 to 8.06) | -1.99 (-2.27 to -1.72) |
| Algeria | 468 (328 to 644) | 6.26 (4.38 to 8.62) | 255 (179 to 348) | 2.72 (1.92 to 3.73) | -2.46 (-2.61 to -2.32) |
| American Samoa | 0 (0 to 0) | 2.11 (1.57 to 2.78) | 0 (0 to 0) | 2.26 (1.5 to 3.18) | 0.43 (0.31 to 0.56) |
| Andorra | 0 (0 to 1) | 7.05 (4.33 to 10.31) | 0 (0 to 0) | 2.35 (1.75 to 3.02) | -3.03 (-3.27 to -2.78) |
| Angola | 266 (131 to 420) | 7.29 (3.64 to 11.43) | 334 (221 to 477) | 3.06 (2.02 to 4.37) | -2.66 (-2.86 to -2.45) |
| Antigua and Barbuda | 0 (0 to 1) | 3.57 (2.81 to 4.41) | 0 (0 to 0) | 3.53 (2.91 to 4.22) | 0.7 (0.43 to 0.96) |
| Argentina | 392 (358 to 427) | 5.74 (5.24 to 6.25) | 222 (186 to 264) | 3.32 (2.76 to 3.95) | -1.29 (-1.48 to -1.11) |
| Armenia | 57 (46 to 69) | 7.76 (6.3 to 9.3) | 15 (11 to 19) | 3.74 (2.82 to 5) | -1.29 (-1.56 to -1.01) |
| Australia | 94 (87 to 102) | 3.72 (3.44 to 4.01) | 59 (50 to 69) | 1.89 (1.6 to 2.2) | -1.81 (-1.95 to -1.67) |
| Austria | 36 (33 to 40) | 3.97 (3.6 to 4.36) | 15 (13 to 18) | 1.75 (1.49 to 2.05) | -2.16 (-2.38 to -1.94) |
| Azerbaijan | 189 (140 to 252) | 11.01 (8.15 to 14.67) | 121 (86 to 171) | 7.65 (5.4 to 10.89) | -1.31 (-1.62 to -0.99) |
| Bahamas | 2 (2 to 3) | 4.25 (3.38 to 5.26) | 1 (1 to 2) | 2.9 (2.18 to 3.93) | -1.19 (-1.52 to -0.86) |
| Bahrain | 4 (3 to 5) | 3.43 (2.75 to 4.25) | 3 (2 to 4) | 1.5 (1.17 to 1.95) | -2.52 (-2.72 to -2.32) |
| Bangladesh | 3368 (1682 to 5310) | 9.43 (4.73 to 14.87) | 1376 (994 to 1863) | 4.56 (3.3 to 6.17) | -2.27 (-2.39 to -2.14) |
| Barbados | 2 (2 to 3) | 5.96 (4.88 to 7.14) | 1 (1 to 2) | 3.79 (2.61 to 5.32) | -0.82 (-1.14 to -0.5) |
| Belarus | 88 (74 to 103) | 5.35 (4.51 to 6.3) | 28 (21 to 37) | 2.69 (1.95 to 3.56) | -1.58 (-2.02 to -1.14) |
| Belgium | 55 (50 to 61) | 4.6 (4.15 to 5.11) | 34 (29 to 40) | 2.72 (2.27 to 3.19) | -2.07 (-2.51 to -1.63) |
| Belize | 4 (3 to 5) | 7 (5.98 to 8.14) | 2 (2 to 3) | 2.78 (2.23 to 3.47) | -2.48 (-2.72 to -2.23) |
| Benin | 140 (100 to 180) | 7.41 (5.29 to 9.56) | 253 (149 to 364) | 5.65 (3.34 to 8.13) | -0.68 (-0.81 to -0.54) |
| Bermuda | 0 (0 to 0) | 4.33 (3.31 to 5.43) | 0 (0 to 0) | 3.3 (2.57 to 4.05) | -0.16 (-0.52 to 0.19) |
| Bhutan | 14 (7 to 20) | 7.43 (3.86 to 11.16) | 6 (4 to 9) | 5.01 (3.32 to 7.35) | -1.55 (-1.86 to -1.23) |
| Bolivia (Plurinational State of) | 293 (190 to 409) | 15.28 (9.94 to 21.27) | 199 (145 to 268) | 8.41 (6.14 to 11.34) | -1.93 (-2 to -1.87) |
| Bosnia and Herzegovina | 32 (23 to 40) | 4.34 (3.2 to 5.54) | 7 (5 to 8) | 2.08 (1.62 to 2.61) | -2.11 (-2.37 to -1.85) |
| Botswana | 11 (7 to 16) | 2.7 (1.8 to 3.82) | 18 (12 to 27) | 3.87 (2.56 to 5.69) | 1.79 (1.48 to 2.1) |
| Brazil | 2475 (2135 to 2815) | 7.25 (6.25 to 8.26) | 1150 (879 to 1402) | 3.53 (2.7 to 4.3) | -1.59 (-2.01 to -1.17) |
| Brunei Darussalam | 3 (3 to 4) | 4.99 (3.9 to 6.33) | 2 (2 to 3) | 3.47 (2.69 to 4.48) | -0.37 (-0.65 to -0.09) |
| Bulgaria | 73 (63 to 82) | 6.64 (5.75 to 7.45) | 18 (15 to 23) | 2.92 (2.35 to 3.6) | -2.35 (-2.65 to -2.05) |
| Burkina Faso | 261 (185 to 343) | 7.27 (5.14 to 9.56) | 459 (291 to 642) | 5.9 (3.76 to 8.25) | -0.28 (-0.46 to -0.11) |
| Burundi | 250 (188 to 331) | 12.38 (9.31 to 16.47) | 243 (124 to 417) | 5.87 (3.01 to 10.04) | -1.63 (-1.97 to -1.28) |
| Cabo Verde | 5 (3 to 7) | 4.45 (2.77 to 6.37) | 4 (3 to 6) | 4.27 (2.95 to 6.46) | -0.45 (-0.71 to -0.19) |
| Cambodia | 286 (144 to 451) | 8.39 (4.28 to 13.16) | 163 (120 to 221) | 4.7 (3.46 to 6.39) | -2.16 (-2.31 to -2.02) |
| Cameroon | 230 (161 to 301) | 6.12 (4.28 to 8.03) | 460 (308 to 626) | 4.86 (3.26 to 6.62) | -0.24 (-0.43 to -0.04) |
| Canada | 130 (121 to 141) | 3.37 (3.14 to 3.64) | 73 (63 to 86) | 1.81 (1.56 to 2.13) | -1.61 (-1.77 to -1.46) |
| Central African Republic | 54 (29 to 83) | 5.8 (3.09 to 8.84) | 65 (40 to 98) | 4.06 (2.48 to 6.06) | -1.01 (-1.13 to -0.9) |
| Chad | 131 (88 to 176) | 5.65 (3.76 to 7.63) | 369 (242 to 503) | 5.42 (3.54 to 7.39) | 0.17 (0.07 to 0.27) |
| Chile | 118 (108 to 130) | 4.29 (3.91 to 4.7) | 47 (40 to 55) | 1.96 (1.67 to 2.3) | -2.01 (-2.27 to -1.75) |
| China | 35950 (27585 to 44528) | 16.53 (12.69 to 20.47) | 6819 (5146 to 8774) | 3.93 (2.95 to 5.1) | -4.79 (-5.05 to -4.53) |
| Colombia | 642 (554 to 733) | 7.99 (6.9 to 9.13) | 285 (221 to 361) | 4.08 (3.15 to 5.18) | -0.81 (-1.28 to -0.33) |
| Comoros | 16 (11 to 22) | 10.49 (7.01 to 14.38) | 13 (9 to 19) | 8.25 (5.45 to 11.7) | -0.81 (-1.09 to -0.53) |
| Congo | 36 (23 to 50) | 4.74 (3.03 to 6.64) | 32 (23 to 43) | 2.46 (1.78 to 3.33) | -2.11 (-2.29 to -1.93) |
| Cook Islands | 0 (0 to 0) | 1.07 (0.73 to 1.52) | 0 (0 to 0) | 1.11 (0.6 to 1.86) | -2.77 (-3.73 to -1.8) |
| Costa Rica | 43 (39 to 47) | 5.47 (5 to 6.01) | 21 (17 to 24) | 3.12 (2.61 to 3.71) | -1.24 (-1.43 to -1.05) |
| Cote d’Ivoire | 207 (144 to 290) | 4.75 (3.3 to 6.66) | 300 (177 to 507) | 3.61 (2.14 to 6.09) | -0.62 (-0.86 to -0.37) |
| Croatia | 27 (23 to 31) | 4.15 (3.53 to 4.86) | 9 (7 to 11) | 2.38 (1.88 to 3.03) | -1.52 (-1.83 to -1.22) |
| Cuba | 90 (81 to 101) | 5.32 (4.78 to 5.97) | 36 (29 to 44) | 3.04 (2.42 to 3.7) | -1.17 (-1.39 to -0.95) |
| Cyprus | 4 (3 to 6) | 3.23 (2.51 to 4.21) | 2 (2 to 3) | 1.44 (1.03 to 1.9) | -1.47 (-1.9 to -1.04) |
| Czechia | 61 (54 to 70) | 4.55 (4 to 5.17) | 19 (15 to 24) | 1.72 (1.36 to 2.17) | -2.74 (-2.99 to -2.48) |
| Democratic People's Republic of Korea | 404 (267 to 570) | 9.27 (6.14 to 13.05) | 144 (89 to 237) | 4.73 (2.92 to 7.76) | -1.95 (-2.15 to -1.76) |
| Democratic Republic of the Congo | 642 (352 to 922) | 4.73 (2.61 to 6.78) | 588 (406 to 835) | 2.22 (1.53 to 3.16) | -1.92 (-2.15 to -1.7) |
| Denmark | 23 (21 to 26) | 4.2 (3.76 to 4.64) | 12 (10 to 15) | 1.94 (1.58 to 2.42) | -2.67 (-3.04 to -2.31) |
| Djibouti | 13 (9 to 18) | 10.44 (7.41 to 14.43) | 19 (11 to 30) | 6.69 (3.85 to 10.44) | -1.15 (-1.53 to -0.77) |
| Dominica | 1 (1 to 1) | 4.76 (3.64 to 6.04) | 1 (1 to 1) | 8.41 (6.21 to 10.97) | 2.14 (1.93 to 2.35) |
| Dominican Republic | 188 (133 to 241) | 9.97 (7.08 to 12.74) | 110 (75 to 157) | 5.46 (3.74 to 7.82) | -1.87 (-2.07 to -1.67) |
| Ecuador | 157 (136 to 182) | 5.95 (5.15 to 6.88) | 153 (118 to 197) | 4.52 (3.48 to 5.82) | -0.46 (-0.9 to -0.03) |
| Egypt | 1176 (848 to 1875) | 7.47 (5.39 to 11.88) | 1063 (815 to 1401) | 4.12 (3.16 to 5.44) | -1.1 (-1.53 to -0.67) |
| El Salvador | 118 (91 to 151) | 7.99 (6.18 to 10.21) | 42 (30 to 59) | 3.38 (2.43 to 4.75) | -2.61 (-2.74 to -2.48) |
| Equatorial Guinea | 7 (4 to 11) | 4.67 (2.45 to 7) | 9 (5 to 15) | 2.29 (1.21 to 3.92) | -2.8 (-3.04 to -2.57) |
| Eritrea | 118 (78 to 165) | 10.02 (6.62 to 14.01) | 150 (97 to 221) | 8.44 (5.45 to 12.42) | -0.5 (-0.7 to -0.31) |
| Estonia | 15 (13 to 17) | 6.22 (5.48 to 6.97) | 3 (2 to 4) | 2.15 (1.7 to 2.65) | -3.15 (-3.62 to -2.67) |
| Eswatini | 10 (6 to 15) | 3.61 (2.34 to 5.36) | 11 (7 to 15) | 3.86 (2.68 to 5.35) | 0.56 (0.34 to 0.78) |
| Ethiopia | 2882 (1149 to 4607) | 15.87 (6.36 to 25.37) | 2537 (1665 to 3928) | 8.27 (5.42 to 12.77) | -2.32 (-2.59 to -2.05) |
| Fiji | 7 (4 to 10) | 3.57 (2.3 to 5.14) | 8 (5 to 11) | 4.19 (2.72 to 6.07) | 0.32 (0.07 to 0.57) |
| Finland | 17 (15 to 19) | 2.62 (2.34 to 2.9) | 11 (9 to 13) | 1.92 (1.61 to 2.33) | -1.64 (-1.99 to -1.3) |
| France | 308 (287 to 330) | 3.92 (3.65 to 4.2) | 181 (155 to 207) | 2.43 (2.08 to 2.79) | -1.46 (-1.7 to -1.22) |
| Gabon | 11 (7 to 14) | 3.55 (2.42 to 4.78) | 11 (7 to 16) | 2.54 (1.64 to 3.77) | -0.56 (-0.78 to -0.33) |
| Gambia | 24 (17 to 32) | 6.88 (4.85 to 8.99) | 30 (19 to 47) | 4.32 (2.81 to 6.76) | -1.71 (-2 to -1.41) |
| Georgia | 64 (52 to 76) | 6.93 (5.65 to 8.24) | 13 (9 to 17) | 2.6 (1.86 to 3.44) | -2.16 (-2.51 to -1.81) |
| Germany | 378 (352 to 404) | 4.3 (4.01 to 4.59) | 176 (154 to 199) | 2.17 (1.9 to 2.47) | -2 (-2.24 to -1.75) |
| Ghana | 385 (239 to 549) | 7.73 (4.79 to 11.06) | 322 (200 to 491) | 3.56 (2.21 to 5.44) | -2.9 (-3.35 to -2.46) |
| Greece | 50 (46 to 54) | 3.94 (3.59 to 4.26) | 22 (19 to 26) | 2.58 (2.16 to 3.01) | -0.76 (-1.04 to -0.49) |
| Greenland | 1 (1 to 1) | 7.16 (4.99 to 9.53) | 0 (0 to 0) | 2.81 (2.1 to 3.74) | -2.88 (-3.15 to -2.61) |
| Grenada | 1 (1 to 2) | 6.29 (5.09 to 7.75) | 1 (0 to 1) | 4.25 (3.39 to 5.32) | -0.45 (-0.69 to -0.2) |
| Guam | 1 (0 to 1) | 2.15 (1.65 to 2.72) | 0 (0 to 1) | 1.82 (1.24 to 2.56) | 1.1 (0.62 to 1.58) |
| Guatemala | 257 (227 to 291) | 8.79 (7.75 to 9.94) | 129 (103 to 160) | 3.94 (3.13 to 4.92) | -1.96 (-2.15 to -1.77) |
| Guinea | 246 (177 to 326) | 11.18 (7.99 to 14.82) | 255 (142 to 424) | 5.85 (3.26 to 9.71) | -1.6 (-1.77 to -1.43) |
| Guinea-Bissau | 29 (20 to 39) | 7.98 (5.42 to 10.88) | 25 (14 to 38) | 3.84 (2.2 to 5.86) | -1.93 (-2.29 to -1.57) |
| Guyana | 11 (9 to 14) | 5.47 (4.35 to 6.65) | 6 (4 to 8) | 4.03 (3.05 to 5.29) | 0.47 (-0.07 to 1.01) |
| Haiti | 360 (144 to 574) | 18.08 (7.34 to 28.79) | 359 (175 to 571) | 11.86 (5.82 to 18.83) | -1.01 (-1.18 to -0.84) |
| Honduras | 142 (112 to 180) | 9.08 (7.18 to 11.53) | 75 (45 to 119) | 3.43 (2.05 to 5.45) | -3.23 (-3.37 to -3.08) |
| Hungary | 63 (57 to 70) | 4.95 (4.49 to 5.45) | 17 (14 to 21) | 1.88 (1.52 to 2.36) | -2.57 (-2.79 to -2.36) |
| Iceland | 2 (2 to 2) | 4.2 (3.66 to 4.78) | 1 (0 to 1) | 1.32 (1.01 to 1.68) | -2.49 (-2.92 to -2.05) |
| India | 13753 (9651 to 18623) | 5.99 (4.2 to 8.11) | 6468 (4803 to 8515) | 2.82 (2.1 to 3.72) | -2.4 (-2.55 to -2.26) |
| Indonesia | 2788 (1518 to 4367) | 6.16 (3.32 to 9.7) | 1752 (1188 to 2408) | 3.94 (2.66 to 5.43) | -1.39 (-1.47 to -1.32) |
| Iran (Islamic Republic of) | 1738 (1268 to 2311) | 9.65 (7.03 to 12.87) | 512 (324 to 689) | 3.68 (2.3 to 5) | -2.37 (-2.84 to -1.9) |
| Iraq | 455 (307 to 643) | 7.78 (5.25 to 10.98) | 407 (294 to 548) | 4.59 (3.3 to 6.18) | -1.3 (-1.53 to -1.07) |
| Ireland | 23 (21 to 26) | 3.69 (3.3 to 4.11) | 10 (8 to 11) | 1.5 (1.26 to 1.76) | -2.16 (-2.46 to -1.87) |
| Israel | 56 (51 to 61) | 5.41 (4.92 to 5.93) | 35 (29 to 42) | 1.94 (1.61 to 2.32) | -2.7 (-2.92 to -2.48) |
| Italy | 303 (289 to 318) | 5.32 (5.08 to 5.59) | 113 (97 to 129) | 2.38 (2.03 to 2.75) | -2.81 (-2.97 to -2.65) |
| Jamaica | 39 (32 to 46) | 6.9 (5.77 to 8.12) | 14 (11 to 17) | 3.73 (2.89 to 4.76) | -1.85 (-2.04 to -1.66) |
| Japan | 499 (480 to 517) | 3.48 (3.35 to 3.61) | 191 (176 to 206) | 1.94 (1.78 to 2.1) | -1.72 (-1.89 to -1.56) |
| Jordan | 69 (56 to 86) | 6.09 (4.94 to 7.54) | 87 (65 to 115) | 3.73 (2.77 to 4.96) | -1.84 (-2.06 to -1.62) |
| Kazakhstan | 297 (261 to 334) | 8.24 (7.24 to 9.27) | 109 (88 to 134) | 2.88 (2.32 to 3.56) | -2.86 (-3.16 to -2.55) |
| Kenya | 551 (401 to 728) | 6.72 (4.9 to 8.87) | 571 (416 to 776) | 4.74 (3.44 to 6.46) | -0.25 (-0.62 to 0.11) |
| Kiribati | 1 (1 to 1) | 3.83 (2.37 to 5.56) | 1 (0 to 2) | 2.96 (1.33 to 5.63) | -0.94 (-1.13 to -0.75) |
| Kuwait | 19 (16 to 23) | 4.88 (4.2 to 5.77) | 11 (9 to 13) | 1.9 (1.55 to 2.35) | -2.12 (-2.52 to -1.72) |
| Kyrgyzstan | 76 (61 to 95) | 6.33 (5.08 to 7.86) | 50 (41 to 61) | 3.15 (2.57 to 3.83) | -1.88 (-2.23 to -1.52) |
| Lao People's Democratic Republic | 118 (44 to 209) | 8.81 (3.39 to 15.57) | 78 (51 to 114) | 4.92 (3.24 to 7.19) | -1.89 (-1.98 to -1.8) |
| Latvia | 23 (20 to 26) | 5.76 (5.14 to 6.47) | 4 (3 to 5) | 1.96 (1.53 to 2.45) | -2.37 (-2.72 to -2.02) |
| Lebanon | 35 (25 to 48) | 4.8 (3.45 to 6.49) | 25 (15 to 39) | 2.94 (1.74 to 4.61) | -1.64 (-1.85 to -1.44) |
| Lesotho | 12 (8 to 17) | 2.51 (1.78 to 3.47) | 15 (10 to 21) | 3.56 (2.4 to 5.1) | 1.72 (1.47 to 1.98) |
| Liberia | 75 (45 to 103) | 8.53 (5.2 to 11.7) | 60 (36 to 87) | 4.04 (2.42 to 5.79) | -2.37 (-2.99 to -1.74) |
| Libya | 90 (67 to 117) | 7.25 (5.45 to 9.46) | 62 (43 to 87) | 6.8 (4.63 to 9.56) | 0.14 (-0.03 to 0.31) |
| Lithuania | 30 (27 to 33) | 5.28 (4.74 to 5.86) | 7 (6 to 9) | 2.64 (2.18 to 3.19) | -2.04 (-2.42 to -1.65) |
| Luxembourg | 2 (2 to 3) | 5.25 (4.61 to 5.95) | 1 (1 to 2) | 2.08 (1.7 to 2.57) | -3.56 (-4.14 to -2.98) |
| Madagascar | 403 (301 to 516) | 9.86 (7.33 to 12.64) | 483 (318 to 709) | 6 (3.95 to 8.81) | -1.25 (-1.43 to -1.08) |
| Malawi | 698 (527 to 888) | 19.55 (14.71 to 24.95) | 569 (273 to 1020) | 10.57 (5.07 to 18.95) | -1.71 (-1.89 to -1.52) |
| Malaysia | 186 (119 to 267) | 4.07 (2.6 to 5.83) | 111 (74 to 152) | 2.2 (1.46 to 3.01) | -1.69 (-2.11 to -1.26) |
| Maldives | 4 (2 to 7) | 5.6 (2.78 to 9.23) | 2 (1 to 3) | 2.89 (2 to 4.01) | -1.88 (-2.03 to -1.74) |
| Mali | 316 (217 to 428) | 9.6 (6.57 to 13.03) | 450 (255 to 756) | 5.17 (2.94 to 8.66) | -1.72 (-1.87 to -1.57) |
| Malta | 2 (2 to 3) | 3.85 (2.86 to 4.51) | 1 (1 to 2) | 3.23 (2.56 to 3.95) | -0.73 (-1.34 to -0.12) |
| Marshall Islands | 0 (0 to 0) | 1.87 (1.2 to 2.74) | 0 (0 to 0) | 2.28 (1.38 to 3.47) | 0.48 (0.1 to 0.87) |
| Mauritania | 36 (24 to 54) | 5.13 (3.44 to 7.7) | 44 (27 to 64) | 3.41 (2.11 to 4.97) | -1.44 (-1.72 to -1.16) |
| Mauritius | 6 (5 to 7) | 2.72 (2.43 to 3.07) | 2 (2 to 2) | 1.47 (1.21 to 1.74) | -2.11 (-2.55 to -1.67) |
| Mexico | 2146 (1937 to 2415) | 9.42 (8.51 to 10.6) | 875 (690 to 1106) | 4.21 (3.3 to 5.35) | -2.13 (-2.41 to -1.85) |
| Micronesia (Federated States of) | 1 (1 to 1) | 2.99 (1.95 to 4.3) | 0 (0 to 1) | 1.88 (1.18 to 2.91) | -1.36 (-1.51 to -1.2) |
| Monaco | 0 (0 to 0) | 5.61 (3.93 to 7.7) | 0 (0 to 0) | 5.26 (4.1 to 6.61) | -2.05 (-2.8 to -1.3) |
| Mongolia | 85 (60 to 115) | 13.17 (9.23 to 17.83) | 34 (25 to 44) | 4.31 (3.15 to 5.64) | -3.45 (-3.8 to -3.09) |
| Montenegro | 7 (5 to 8) | 6.36 (4.98 to 7.91) | 1 (1 to 2) | 1.91 (1 to 2.94) | -3.45 (-3.87 to -3.03) |
| Morocco | 280 (184 to 392) | 4.11 (2.7 to 5.75) | 141 (93 to 209) | 2.15 (1.42 to 3.2) | -1.68 (-1.9 to -1.46) |
| Mozambique | 695 (477 to 946) | 14.96 (10.24 to 20.41) | 823 (450 to 1463) | 8.13 (4.45 to 14.45) | -1.59 (-1.79 to -1.39) |
| Myanmar | 1019 (391 to 1761) | 10.22 (3.9 to 17.67) | 614 (395 to 912) | 5.9 (3.79 to 8.78) | -1.98 (-2.12 to -1.84) |
| Namibia | 17 (11 to 24) | 3.99 (2.68 to 5.58) | 27 (18 to 41) | 4.98 (3.29 to 7.49) | 1.37 (0.98 to 1.77) |
| Nauru | 0 (0 to 0) | 3.7 (2.65 to 5) | 0 (0 to 0) | 3.67 (2.49 to 5.18) | -0.09 (-0.46 to 0.28) |
| Nepal | 464 (251 to 708) | 7.53 (4.09 to 11.45) | 216 (153 to 303) | 3.52 (2.49 to 4.93) | -2.18 (-2.34 to -2.02) |
| Netherlands | 71 (65 to 76) | 3.88 (3.55 to 4.19) | 33 (29 to 39) | 1.9 (1.63 to 2.21) | -2.56 (-2.76 to -2.37) |
| New Zealand | 23 (20 to 25) | 4.21 (3.76 to 4.72) | 13 (11 to 15) | 1.95 (1.7 to 2.26) | -2.13 (-2.36 to -1.89) |
| Nicaragua | 131 (100 to 168) | 10.13 (7.77 to 13.05) | 43 (31 to 60) | 3.25 (2.34 to 4.55) | -2.98 (-3.19 to -2.77) |
| Niger | 297 (203 to 402) | 9.3 (6.35 to 12.57) | 398 (226 to 622) | 4.14 (2.36 to 6.46) | -2.55 (-2.78 to -2.31) |
| Nigeria | 2638 (2031 to 3349) | 8.84 (6.8 to 11.23) | 5123 (2778 to 7456) | 7.14 (3.88 to 10.38) | -0.42 (-0.55 to -0.29) |
| Niue | 0 (0 to 0) | 3.06 (2.09 to 4.4) | 0 (0 to 0) | 14.02 (10.55 to 18.64) | 1.61 (0.45 to 2.79) |
| North Macedonia | 32 (25 to 39) | 9.18 (7.24 to 11.32) | 7 (5 to 9) | 3.09 (2.12 to 4.26) | -2.74 (-3.16 to -2.32) |
| Northern Mariana Islands | 0 (0 to 0) | 1.35 (0.94 to 1.92) | 0 (0 to 0) | 1.13 (0.79 to 1.58) | -0.05 (-0.26 to 0.15) |
| Norway | 22 (21 to 24) | 4.2 (3.93 to 4.49) | 9 (8 to 10) | 1.48 (1.28 to 1.72) | -2.77 (-3.01 to -2.53) |
| Oman | 19 (14 to 27) | 3.19 (2.3 to 4.35) | 12 (9 to 16) | 1.44 (1.06 to 1.82) | -1.77 (-2.13 to -1.41) |
| Pakistan | 2966 (2000 to 4078) | 8.42 (5.69 to 11.58) | 5003 (3762 to 6551) | 8.53 (6.42 to 11.18) | 0.6 (0.4 to 0.8) |
| Palau | 0 (0 to 0) | 2.04 (1.41 to 2.85) | 0 (0 to 0) | 1.43 (1.06 to 1.96) | -0.92 (-1.08 to -0.76) |
| Palestine | 48 (32 to 68) | 6.79 (4.51 to 9.46) | 50 (37 to 65) | 3.94 (2.95 to 5.16) | -1.16 (-1.45 to -0.87) |
| Panama | 46 (39 to 55) | 8.22 (6.93 to 9.65) | 40 (32 to 49) | 5.2 (4.19 to 6.43) | -1.56 (-1.72 to -1.4) |
| Papua New Guinea | 45 (24 to 73) | 3.72 (1.95 to 6.01) | 101 (61 to 165) | 3.57 (2.16 to 5.8) | -0.09 (-0.35 to 0.18) |
| Paraguay | 75 (59 to 96) | 6.35 (4.94 to 8.11) | 59 (42 to 83) | 4.46 (3.13 to 6.34) | -0.62 (-0.99 to -0.25) |
| Peru | 624 (492 to 831) | 10.96 (8.63 to 14.57) | 330 (219 to 464) | 5.07 (3.37 to 7.13) | -2.12 (-2.27 to -1.97) |
| Philippines | 1354 (907 to 1911) | 7.7 (5.17 to 10.84) | 1098 (846 to 1376) | 4.85 (3.73 to 6.09) | -0.88 (-1.08 to -0.69) |
| Poland | 393 (363 to 418) | 6.3 (5.8 to 6.7) | 77 (66 to 88) | 2.01 (1.71 to 2.32) | -3.5 (-3.66 to -3.33) |
| Portugal | 89 (80 to 97) | 6.94 (6.28 to 7.62) | 23 (19 to 27) | 2.64 (2.25 to 3.1) | -3.62 (-4.02 to -3.22) |
| Puerto Rico | 25 (22 to 28) | 3.9 (3.48 to 4.36) | 3 (3 to 4) | 1.42 (1.16 to 1.73) | -2.25 (-2.55 to -1.95) |
| Qatar | 2 (2 to 3) | 2.64 (1.95 to 3.58) | 4 (3 to 6) | 1.21 (0.9 to 1.65) | -2.28 (-2.44 to -2.11) |
| Republic of Korea | 429 (326 to 537) | 5.77 (4.38 to 7.23) | 69 (47 to 90) | 1.86 (1.23 to 2.45) | -3.82 (-4.1 to -3.54) |
| Republic of Moldova | 104 (90 to 121) | 12.21 (10.55 to 14.2) | 13 (10 to 17) | 4.01 (3.13 to 5.14) | -3.07 (-3.51 to -2.63) |
| Romania | 320 (281 to 358) | 9.12 (8.02 to 10.22) | 54 (46 to 63) | 2.82 (2.38 to 3.3) | -3.68 (-3.96 to -3.39) |
| Russian Federation | 1935 (1867 to 2008) | 8.2 (7.91 to 8.52) | 439 (393 to 484) | 2.6 (2.31 to 2.87) | -4.07 (-4.4 to -3.73) |
| Rwanda | 360 (265 to 467) | 14.14 (10.44 to 18.34) | 244 (162 to 357) | 7.16 (4.75 to 10.45) | -2.39 (-2.65 to -2.12) |
| Saint Kitts and Nevis | 1 (0 to 1) | 5.65 (4.76 to 6.63) | 0 (0 to 0) | 4.18 (3.27 to 5.28) | -0.58 (-0.79 to -0.37) |
| Saint Lucia | 2 (2 to 2) | 5.4 (4.37 to 6.52) | 1 (1 to 1) | 3.96 (3.05 to 5.15) | -1 (-1.23 to -0.76) |
| Saint Vincent and the Grenadines | 2 (2 to 3) | 7.38 (5.87 to 9.37) | 1 (1 to 1) | 5.03 (4.04 to 6.2) | -1.13 (-1.5 to -0.75) |
| Samoa | 2 (1 to 3) | 4 (2.56 to 5.68) | 2 (1 to 3) | 2.75 (1.56 to 4.65) | -1.19 (-1.31 to -1.06) |
| San Marino | 0 (0 to 0) | 7.38 (5.35 to 9.94) | 0 (0 to 0) | 3.13 (2.17 to 4.33) | -2.35 (-2.49 to -2.21) |
| Sao Tome and Principe | 3 (2 to 4) | 6.4 (4.48 to 8.79) | 1 (1 to 2) | 2.15 (1.12 to 3.56) | -3.17 (-3.75 to -2.58) |
| Saudi Arabia | 204 (145 to 282) | 4.37 (3.1 to 6.03) | 57 (39 to 84) | 1.13 (0.76 to 1.67) | -4.45 (-4.54 to -4.37) |
| Senegal | 190 (144 to 248) | 6.83 (5.15 to 8.91) | 167 (103 to 263) | 3.8 (2.34 to 5.96) | -1.56 (-1.82 to -1.3) |
| Serbia | 105 (76 to 138) | 7.48 (5.39 to 9.83) | 15 (11 to 20) | 1.86 (1.38 to 2.47) | -5.1 (-5.58 to -4.61) |
| Seychelles | 1 (0 to 1) | 3.4 (2.53 to 4.48) | 0 (0 to 0) | 2.09 (1.54 to 2.77) | -0.91 (-1.42 to -0.4) |
| Sierra Leone | 124 (85 to 166) | 8.54 (5.86 to 11.45) | 132 (87 to 187) | 5.13 (3.39 to 7.3) | -1.54 (-1.68 to -1.39) |
| Singapore | 18 (16 to 20) | 4.23 (3.75 to 4.77) | 9 (7 to 10) | 1.5 (1.24 to 1.79) | -2.48 (-2.87 to -2.09) |
| Slovakia | 38 (33 to 45) | 4.46 (3.83 to 5.21) | 16 (12 to 20) | 2.74 (2.06 to 3.58) | -1.46 (-1.58 to -1.34) |
| Slovenia | 9 (8 to 10) | 3.28 (2.89 to 3.69) | 2 (2 to 3) | 1.18 (0.97 to 1.44) | -2.81 (-3.13 to -2.49) |
| Solomon Islands | 3 (2 to 4) | 2.51 (1.48 to 3.78) | 4 (2 to 7) | 2.31 (1.34 to 3.65) | -0.19 (-0.53 to 0.15) |
| Somalia | 268 (156 to 398) | 9.14 (5.33 to 13.59) | 400 (215 to 637) | 5.2 (2.79 to 8.27) | -1.3 (-1.7 to -0.89) |
| South Africa | 333 (238 to 438) | 3.54 (2.54 to 4.64) | 284 (220 to 355) | 2.8 (2.17 to 3.51) | -0.61 (-0.91 to -0.31) |
| South Sudan | 233 (152 to 326) | 11.98 (7.8 to 16.74) | 309 (200 to 433) | 10.35 (6.72 to 14.52) | 0.09 (-0.42 to 0.6) |
| Spain | 216 (199 to 234) | 4.63 (4.26 to 5.01) | 99 (88 to 113) | 2.42 (2.15 to 2.76) | -2.29 (-2.4 to -2.17) |
| Sri Lanka | 190 (147 to 247) | 5.12 (3.96 to 6.65) | 59 (41 to 84) | 1.76 (1.24 to 2.52) | -3.87 (-4.33 to -3.41) |
| Sudan | 772 (354 to 1276) | 11.82 (5.49 to 19.48) | 749 (502 to 1067) | 6.68 (4.47 to 9.52) | -1.51 (-1.67 to -1.35) |
| Suriname | 6 (4 to 8) | 7.17 (4.93 to 8.99) | 5 (4 to 7) | 5.8 (4.26 to 7.56) | -0.51 (-0.74 to -0.28) |
| Sweden | 44 (39 to 48) | 4.16 (3.75 to 4.6) | 20 (17 to 24) | 1.69 (1.43 to 2.03) | -2.51 (-2.68 to -2.34) |
| Switzerland | 29 (27 to 32) | 3.74 (3.39 to 4.12) | 19 (16 to 22) | 2.1 (1.76 to 2.52) | -2.12 (-2.45 to -1.78) |
| Syrian Arab Republic | 297 (188 to 416) | 7.11 (4.53 to 9.96) | 64 (44 to 91) | 3.02 (2.03 to 4.3) | -2.5 (-2.84 to -2.15) |
| Taiwan (Province of China) | 128 (118 to 139) | 3.65 (3.36 to 3.98) | 37 (32 to 44) | 1.93 (1.62 to 2.26) | -1.72 (-1.94 to -1.51) |
| Tajikistan | 203 (144 to 280) | 11.66 (8.3 to 15.98) | 208 (148 to 293) | 8.14 (5.78 to 11.44) | -1.34 (-1.58 to -1.1) |
| Thailand | 696 (504 to 911) | 6.36 (4.6 to 8.34) | 190 (148 to 236) | 3.1 (2.4 to 3.86) | -2.63 (-2.91 to -2.36) |
| Timor-Leste | 21 (9 to 36) | 8.23 (3.47 to 13.96) | 16 (10 to 24) | 4.55 (2.91 to 6.87) | -2.12 (-2.41 to -1.83) |
| Togo | 68 (50 to 90) | 5.17 (3.83 to 6.86) | 81 (44 to 128) | 3.51 (1.91 to 5.58) | -1.08 (-1.2 to -0.95) |
| Tokelau | 0 (0 to 0) | 3.01 (1.91 to 4.49) | 0 (0 to 0) | 20.34 (10.96 to 32.9) | 1.02 (-0.96 to 3.04) |
| Tonga | 1 (1 to 1) | 3.22 (2.18 to 4.45) | 1 (1 to 1) | 2.98 (2.04 to 4.52) | -0.43 (-0.77 to -0.08) |
| Trinidad and Tobago | 18 (15 to 21) | 6.3 (5.3 to 7.35) | 7 (5 to 9) | 3.87 (3.01 to 5.05) | -1.22 (-1.4 to -1.04) |
| Tunisia | 150 (113 to 194) | 7.07 (5.31 to 9.17) | 51 (36 to 70) | 2.74 (1.94 to 3.8) | -2.82 (-2.91 to -2.73) |
| Turkey | 1523 (1058 to 2055) | 11.13 (7.72 to 15.01) | 446 (343 to 577) | 3.72 (2.85 to 4.82) | -3.52 (-3.77 to -3.26) |
| Turkmenistan | 107 (89 to 126) | 9.75 (8.15 to 11.43) | 60 (49 to 73) | 5.68 (4.6 to 6.88) | -1.27 (-1.52 to -1.02) |
| Tuvalu | 0 (0 to 0) | 3.73 (1.94 to 5.98) | 0 (0 to 0) | 2.06 (1.38 to 2.97) | -1.69 (-1.83 to -1.56) |
| Uganda | 823 (586 to 1170) | 12.37 (8.79 to 17.56) | 1418 (790 to 2295) | 10.08 (5.65 to 16.27) | -0.43 (-0.64 to -0.23) |
| Ukraine | 713 (594 to 841) | 9.32 (7.76 to 10.99) | 158 (127 to 192) | 4.17 (3.28 to 5.14) | -2.5 (-2.7 to -2.31) |
| United Arab Emirates | 16 (12 to 23) | 3.71 (2.61 to 5.16) | 15 (10 to 21) | 1.6 (1.07 to 2.27) | -1.72 (-2.08 to -1.37) |
| United Kingdom | 305 (294 to 317) | 4.08 (3.93 to 4.24) | 146 (132 to 158) | 1.91 (1.72 to 2.06) | -1.86 (-2.16 to -1.56) |
| United Republic of Tanzania | 1182 (905 to 1470) | 13.09 (10.02 to 16.29) | 1602 (1064 to 2300) | 9.34 (6.22 to 13.39) | -0.48 (-0.73 to -0.23) |
| United States of America | 1389 (1358 to 1421) | 3.63 (3.54 to 3.71) | 801 (732 to 874) | 2.09 (1.9 to 2.28) | -1.41 (-1.52 to -1.31) |
| United States Virgin Islands | 1 (1 to 1) | 3.85 (2.77 to 5.01) | 0 (0 to 0) | 1.63 (0.91 to 2.67) | -1.23 (-1.8 to -0.66) |
| Uruguay | 29 (25 to 32) | 5.25 (4.68 to 5.92) | 12 (9 to 15) | 2.65 (2.13 to 3.34) | -2.38 (-2.73 to -2.03) |
| Uzbekistan | 463 (372 to 568) | 7.41 (5.97 to 9.06) | 466 (372 to 580) | 6.47 (5.17 to 8.03) | -0.22 (-0.33 to -0.11) |
| Vanuatu | 1 (1 to 1) | 1.89 (1.16 to 2.85) | 1 (1 to 2) | 1.78 (1.05 to 2.66) | 0.1 (-0.34 to 0.53) |
| Venezuela (Bolivarian Republic of) | 334 (308 to 361) | 6.85 (6.31 to 7.41) | 210 (155 to 273) | 4.77 (3.51 to 6.22) | -0.7 (-1.02 to -0.38) |
| Viet Nam | 949 (678 to 1262) | 5.14 (3.66 to 6.83) | 479 (313 to 686) | 2.86 (1.87 to 4.1) | -1.74 (-1.83 to -1.64) |
| Yemen | 388 (196 to 642) | 7.4 (3.76 to 12.2) | 447 (294 to 642) | 4.78 (3.15 to 6.87) | -1.24 (-1.38 to -1.09) |
| Zambia | 405 (309 to 512) | 14.23 (10.83 to 18) | 447 (283 to 681) | 7.8 (4.94 to 11.88) | -1.69 (-1.85 to -1.53) |
| Zimbabwe | 113 (75 to 152) | 3.33 (2.21 to 4.46) | 242 (166 to 336) | 5.58 (3.83 to 7.75) | 3.03 (2.33 to 3.73) |

EAPC, estimated annual percentage change, SDl, Sociodemographic Index; Ul, uncertainty interval. EAPC is expressed as 95% CIs.

**Supplementary Table 4** The DALYs cases and age-standardized DALYs rate of Urogenital Congenital Anomalies in Children Under 9 Years in 1990 and 2021 across 204 countries worldwide, along with their temporal trend.

|  | Rate per 100 000(95%UI) | | 2021 | | 1990-2021 |
| --- | --- | --- | --- | --- | --- |
|  | 1990 | |  | |  |
|  | DALYs cases | The age-standardized DALYs rate | DALYs cases | The age-standardized DALYs rate | EAPC |

| Afghanistan | 23006 (8460 to 38642) | | 744.22 (282.07 to 1244.23) | | 55154 (36272 to 81479) | 538.7 (355.58 to 792.37) | | -0.72 (-0.97 to -0.48) | |
| --- | --- | --- | --- | --- | --- | --- | --- | --- | --- |
| Albania | 7400 (4939 to 9343) | | 949.02 (634.32 to 1197.48) | | 1494 (1096 to 2028) | 520.63 (381.86 to 707.07) | | -1.96 (-2.22 to -1.7) | |
| Algeria | 40455 (28323 to 55631) | | 541.08 (378.85 to 744.68) | | 22159 (15627 to 30297) | 236.97 (167.05 to 324.18) | | -2.43 (-2.58 to -2.29) | |
| American Samoa | 25 (19 to 33) | | 183.14 (136.97 to 241.02) | | 17 (11 to 23) | 197.33 (130.54 to 277.73) | | 0.44 (0.32 to 0.56) | |
| Andorra | 36 (22 to 52) | | 630.74 (385.03 to 921.17) | | 12 (9 to 16) | 212.62 (157.63 to 274.93) | | -2.99 (-3.23 to -2.74) | |
| Angola | 23245 (11493 to 36615) | | 634.94 (317.64 to 995.31) | | 28974 (19133 to 41307) | 265.14 (175.17 to 377.92) | | -2.67 (-2.88 to -2.47) | |
| Antigua and Barbuda | 38 (30 to 47) | | 309.79 (243.63 to 382.26) | | 33 (27 to 39) | 307.38 (253.2 to 368.62) | | 0.71 (0.45 to 0.97) | |
| Argentina | 33923 (30994 to 36950) | | 496.48 (453.61 to 540.81) | | 19206 (16027 to 22852) | 287.52 (239.03 to 343.58) | | -1.29 (-1.47 to -1.1) | |
| Armenia | 4933 (3999 to 5921) | | 669.08 (542.61 to 802.44) | | 1270 (961 to 1690) | 324.77 (244.26 to 435.35) | | -1.27 (-1.55 to -1) | |
| Australia | 8248 (7627 to 8900) | | 325.77 (301.2 to 351.53) | | 5282 (4468 to 6168) | 168.29 (142.02 to 196.89) | | -1.73 (-1.87 to -1.59) | |
| Austria | 3181 (2883 to 3500) | | 350.32 (317.49 to 385.54) | | 1372 (1162 to 1607) | 158.64 (134.25 to 186) | | -2.09 (-2.3 to -1.88) | |
| Azerbaijan | 16338 (12076 to 21772) | | 950.19 (702.69 to 1265.31) | | 10327 (7329 to 14655) | 657.75 (463.96 to 937.75) | | -1.3 (-1.61 to -0.99) | |
| Bahamas | 196 (156 to 242) | | 368.4 (291.95 to 456.4) | | 119 (90 to 160) | 251.69 (188.23 to 342.66) | | -1.17 (-1.49 to -0.84) | |
| Bahrain | 351 (282 to 434) | | 297.19 (238.74 to 368.06) | | 261 (202 to 339) | 131.13 (101.46 to 170.3) | | -2.47 (-2.66 to -2.28) | |
| Bangladesh | 291084 (145461 to 458441) | | 814.16 (408.3 to 1281.88) | | 117859 (85200 to 159356) | 391.11 (282.82 to 528.89) | | -2.29 (-2.41 to -2.16) | |
| Barbados | 208 (170 to 249) | | 515.47 (420.83 to 617.17) | | 96 (66 to 134) | 330.48 (227.62 to 464.77) | | -0.8 (-1.12 to -0.49) | |
| Belarus | 7585 (6398 to 8923) | | 462.99 (390.29 to 544.83) | | 2452 (1804 to 3218) | 236.66 (171.05 to 314.39) | | -1.53 (-1.96 to -1.09) | |
| Belgium | 4846 (4366 to 5402) | | 405.11 (364.81 to 451.83) | | 3038 (2535 to 3571) | 241.48 (200.77 to 284.56) | | -2.02 (-2.45 to -1.59) | |
| Belize | 350 (298 to 407) | | 607.81 (518.7 to 706.75) | | 190 (153 to 237) | 242.43 (194.73 to 302.66) | | -2.46 (-2.71 to -2.22) | |
| Benin | 12209 (8736 to 15737) | | 647.21 (462.72 to 835.67) | | 22030 (12962 to 31777) | 492.04 (290.74 to 708.04) | | -0.68 (-0.82 to -0.55) | |
| Bermuda | 31 (24 to 39) | | 377.57 (288.91 to 473.53) | | 15 (12 to 19) | 292.88 (228.07 to 360.98) | | -0.11 (-0.46 to 0.25) | |
| Bhutan | 1189 (624 to 1785) | | 646.81 (337.63 to 970.99) | | 529 (352 to 776) | 434.18 (288.46 to 637.15) | | -1.56 (-1.87 to -1.24) | |
| Bolivia (Plurinational State of) | 25397 (16413 to 35483) | | 1321.77 (857.33 to 1842.49) | | 17177 (12536 to 23176) | 726.12 (529.68 to 979.99) | | -1.94 (-2 to -1.87) | |
| Bosnia and Herzegovina | 2705 (1999 to 3452) | | 372.41 (274.74 to 476.18) | | 579 (452 to 726) | 179.31 (139.61 to 225.58) | | -2.08 (-2.34 to -1.82) | |
| Botswana | 957 (642 to 1355) | | 232.8 (156.28 to 329.54) | | 1564 (1038 to 2302) | 334.65 (221.62 to 493.3) | | 1.8 (1.49 to 2.12) | |
| Brazil | 214020 (184762 to 243546) | | 628.44 (542.19 to 716.17) | | 99336 (76063 to 121361) | 304.79 (233.37 to 372.4) | | -1.6 (-2.02 to -1.18) | |
| Brunei Darussalam | 280 (219 to 353) | | 432.14 (338.95 to 546.06) | | 190 (148 to 246) | 305.66 (237.35 to 395.77) | | -0.34 (-0.61 to -0.07) | |
| Bulgaria | 6285 (5454 to 7048) | | 573.36 (497.08 to 643.49) | | 1591 (1279 to 1958) | 252.56 (202.59 to 311.16) | | -2.34 (-2.64 to -2.04) | |
| Burkina Faso | 22862 (16231 to 29996) | | 635.95 (450.96 to 835.22) | | 40119 (25452 to 56182) | 515.43 (328.41 to 720.62) | | -0.29 (-0.47 to -0.11) | |
| Burundi | 21725 (16391 to 28796) | | 1075.98 (810.68 to 1429.34) | | 21024 (10655 to 36136) | 507.22 (258.62 to 869.06) | | -1.64 (-1.99 to -1.29) | |
| Cabo Verde | 448 (281 to 639) | | 390.26 (243.94 to 557.44) | | 339 (235 to 511) | 371.32 (256.3 to 563.16) | | -0.46 (-0.72 to -0.21) | |
| Cambodia | 24707 (12386 to 38975) | | 723.15 (367.11 to 1136.02) | | 13983 (10322 to 19019) | 403.76 (298.1 to 549.2) | | -2.17 (-2.32 to -2.02) | |
| Cameroon | 20093 (14105 to 26252) | | 533.27 (373.54 to 698.3) | | 39944 (26823 to 54401) | 422.26 (283.6 to 574.92) | | -0.25 (-0.45 to -0.05) | |
| Canada | 11562 (10760 to 12527) | | 299.41 (278.58 to 324.49) | | 6579 (5650 to 7723) | 163.25 (139.82 to 192.14) | | -1.54 (-1.7 to -1.39) | |
| Central African Republic | 4744 (2502 to 7244) | | 504.29 (268.27 to 768.13) | | 5664 (3443 to 8450) | 351.76 (214.4 to 523.74) | | -1.03 (-1.14 to -0.91) | |
| Chad | 11454 (7675 to 15386) | | 492.82 (329.09 to 664.13) | | 32087 (21079 to 43704) | 470.25 (308.72 to 641.14) | | 0.16 (0.06 to 0.26) | |
| Chile | 10198 (9283 to 11167) | | 369.52 (336.48 to 404.62) | | 4118 (3513 to 4792) | 171.5 (145.53 to 200.79) | | -1.96 (-2.22 to -1.71) | |
| China | 3134060 (2405214 to 3883249) | | 1440.94 (1106.16 to 1784.58) | | 600081 (449796 to 777997) | 348.04 (258.99 to 454.6) | | -4.73 (-4.99 to -4.47) | |
| Colombia | 55245 (47639 to 63142) | | 687.39 (593.07 to 785.28) | | 24617 (19036 to 31331) | 352.75 (271.96 to 450.09) | | -0.78 (-1.25 to -0.31) | |
| Comoros | 1424 (959 to 1949) | | 912.88 (611.96 to 1250.17) | | 1152 (761 to 1635) | 715.15 (471.75 to 1015.93) | | -0.82 (-1.09 to -0.54) | |
| Congo | 3114 (1993 to 4366) | | 412.28 (264.34 to 577.46) | | 2720 (1973 to 3684) | 212.61 (154.06 to 288.01) | | -2.13 (-2.31 to -1.95) | |
| Cook Islands | 4 (3 to 6) | | 93.6 (64.01 to 132.4) | | 2 (1 to 4) | 99.22 (54.14 to 167.81) | | -2.69 (-3.65 to -1.72) | |
| Costa Rica | 3735 (3421 to 4106) | | 474.74 (434.84 to 521.92) | | 1789 (1497 to 2119) | 272.81 (227.06 to 324.49) | | -1.23 (-1.42 to -1.04) | |
| Cote d’Ivoire | 18034 (12537 to 25333) | | 413.95 (287.69 to 581.07) | | 26026 (15286 to 43883) | 313.14 (184.87 to 526.64) | | -0.64 (-0.89 to -0.39) | |
| Croatia | 2313 (1966 to 2704) | | 359.74 (305.21 to 421.37) | | 797 (634 to 1013) | 210.09 (166.41 to 268.16) | | -1.46 (-1.76 to -1.16) | |
| Cuba | 7788 (7006 to 8773) | | 460.43 (414.32 to 518.38) | | 3105 (2496 to 3776) | 263.88 (210.81 to 322.47) | | -1.14 (-1.36 to -0.92) | |
| Cyprus | 374 (291 to 486) | | 282.08 (219.11 to 367.28) | | 193 (137 to 256) | 130.58 (92.69 to 172.77) | | -1.36 (-1.78 to -0.93) | |
| Czechia | 5286 (4656 to 6008) | | 392.95 (345.53 to 447.4) | | 1695 (1346 to 2151) | 150.43 (119.26 to 191.14) | | -2.69 (-2.94 to -2.43) | |
| Democratic People's Republic of Korea | 35313 (23353 to 49796) | | 808.5 (535.21 to 1138.19) | | 12555 (7763 to 20661) | 412.11 (254.6 to 678.51) | | -1.95 (-2.15 to -1.76) | |
| Democratic Republic of the Congo | 56019 (30673 to 80415) | | 411.48 (227.53 to 589.68) | | 50719 (35003 to 72025) | 191.7 (132.34 to 272.17) | | -1.95 (-2.17 to -1.72) | |
| Denmark | 2057 (1838 to 2268) | | 367.73 (328.54 to 405.44) | | 1082 (880 to 1341) | 175.14 (142.27 to 217.14) | | -2.55 (-2.91 to -2.19) | |
| Djibouti | 1126 (800 to 1562) | | 910.02 (646.62 to 1260.53) | | 1653 (949 to 2583) | 580.13 (333.44 to 906.61) | | -1.16 (-1.54 to -0.78) | |
| Dominica | 69 (53 to 87) | | 410.97 (313.43 to 520.82) | | 59 (44 to 76) | 729.35 (537.16 to 953.35) | | 2.16 (1.94 to 2.37) | |
| Dominican Republic | 16384 (11594 to 20958) | | 866.07 (614.4 to 1106.81) | | 9469 (6470 to 13569) | 471.74 (322.52 to 675.69) | | -1.88 (-2.08 to -1.68) | |
| Ecuador | 13565 (11729 to 15649) | | 513.41 (443.9 to 592.29) | | 13161 (10150 to 16930) | 388.82 (298.91 to 501.62) | | -0.48 (-0.91 to -0.05) | |
| Egypt | 101929 (73495 to 162306) | | 645.77 (466.07 to 1026.37) | | 91961 (70444 to 121368) | 356.98 (273.37 to 471.22) | | -1.1 (-1.53 to -0.68) | |
| El Salvador | 10195 (7878 to 13044) | | 690.07 (533.34 to 882.59) | | 3605 (2598 to 5062) | 290.26 (208.39 to 409.08) | | -2.63 (-2.76 to -2.5) | |
| Equatorial Guinea | 614 (318 to 922) | | 405.49 (212.69 to 606.71) | | 760 (404 to 1299) | 198.35 (105.28 to 337.98) | | -2.81 (-3.05 to -2.57) | |
| Eritrea | 10297 (6800 to 14387) | | 870.67 (575.75 to 1216.16) | | 13029 (8405 to 19148) | 732.34 (472.59 to 1076.19) | | -0.51 (-0.71 to -0.31) | |
| Estonia | 1291 (1140 to 1453) | | 543.03 (479.57 to 610.87) | | 280 (221 to 350) | 200.81 (158.22 to 250.97) | | -2.93 (-3.41 to -2.45) | |
| Eswatini | 862 (558 to 1280) | | 312.37 (202.76 to 463.13) | | 928 (644 to 1287) | 332.73 (231.04 to 461.89) | | 0.55 (0.33 to 0.77) | |
| Ethiopia | 250575 (99874 to 400296) | | 1377.59 (551.95 to 2200.34) | | 219646 (144030 to 340302) | 715.02 (468.95 to 1105.5) | | -2.33 (-2.61 to -2.05) | |
| Fiji | 591 (381 to 848) | | 306.69 (197.7 to 440.74) | | 665 (433 to 964) | 361.89 (235.43 to 524.58) | | 0.35 (0.11 to 0.6) | |
| Finland | 1473 (1318 to 1634) | | 228.68 (204.64 to 253.89) | | 945 (798 to 1139) | 172.04 (144.62 to 208.11) | | -1.51 (-1.85 to -1.18) | |
| France | 27079 (25238 to 29072) | | 345.18 (321.66 to 370.66) | | 16277 (13944 to 18747) | 219.88 (187.82 to 253.79) | | -1.36 (-1.6 to -1.12) | |
| Gabon | 913 (619 to 1228) | | 308.51 (209.66 to 414.54) | | 942 (610 to 1399) | 219.28 (141.64 to 326.22) | | -0.57 (-0.8 to -0.34) | |
| Gambia | 2133 (1512 to 2785) | | 601.91 (425.56 to 786.75) | | 2609 (1696 to 4086) | 376.16 (244.69 to 588.63) | | -1.72 (-2.02 to -1.43) | |
| Georgia | 5536 (4513 to 6575) | | 596.7 (486.16 to 708.9) | | 1132 (811 to 1491) | 226.19 (161.84 to 298.57) | | -2.14 (-2.48 to -1.8) | |
| Germany | 32988 (30754 to 35189) | | 375.57 (350.13 to 400.62) | | 15721 (13655 to 17885) | 194.46 (168.73 to 221.42) | | -1.93 (-2.17 to -1.69) | |
| Ghana | 33422 (20775 to 47612) | | 670.87 (415.67 to 957.67) | | 27904 (17297 to 42664) | 308.94 (191.58 to 472) | | -2.9 (-3.34 to -2.45) | |
| Greece | 4391 (4003 to 4753) | | 347.71 (316.96 to 376.57) | | 2005 (1681 to 2343) | 230.86 (193.39 to 269.96) | | -0.74 (-1.01 to -0.47) | |
| Greenland | 64 (44 to 85) | | 618.28 (430.6 to 824.22) | | 19 (14 to 26) | 243.1 (181.96 to 324.54) | | -2.86 (-3.12 to -2.59) | |
| Grenada | 126 (102 to 156) | | 542.15 (438.2 to 668.82) | | 52 (41 to 65) | 367.23 (292.78 to 460.22) | | -0.44 (-0.68 to -0.2) | |
| Guam | 55 (42 to 70) | | 186.65 (144.12 to 236.24) | | 41 (28 to 57) | 163.88 (111.85 to 229.28) | | 1.19 (0.72 to 1.65) | |
| Guatemala | 22304 (19661 to 25194) | | 761.17 (671.11 to 859.75) | | 11144 (8867 to 13893) | 342.05 (270.94 to 427.95) | | -1.96 (-2.15 to -1.77) | |
| Guinea | 21633 (15549 to 28615) | | 979.93 (702.15 to 1297.89) | | 22296 (12423 to 37025) | 511.02 (285.24 to 848.17) | | -1.61 (-1.78 to -1.44) | |
| Guinea-Bissau | 2510 (1712 to 3418) | | 695.8 (473.52 to 948.12) | | 2132 (1217 to 3250) | 332.92 (190.23 to 507.24) | | -1.95 (-2.31 to -1.59) | |
| Guyana | 958 (761 to 1168) | | 471.02 (374.65 to 572.53) | | 506 (382 to 667) | 346.88 (262.2 to 457.16) | | 0.46 (-0.08 to 1) | |
| Haiti | 31268 (12445 to 49830) | | 1567.58 (633.96 to 2493.35) | | 31073 (15139 to 49418) | 1025.47 (501.88 to 1629.23) | | -1.02 (-1.18 to -0.85) | |
| Honduras | 12170 (9637 to 15456) | | 778.15 (616.18 to 988.45) | | 6472 (3880 to 10256) | 295.72 (177.07 to 469.29) | | -3.21 (-3.35 to -3.07) | |
| Hungary | 5459 (4953 to 6008) | | 427.47 (387.58 to 470.88) | | 1510 (1216 to 1887) | 165.47 (133.05 to 207.19) | | -2.53 (-2.74 to -2.32) | |
| Iceland | 159 (138 to 181) | | 370.58 (323.03 to 422.2) | | 54 (41 to 68) | 123.83 (94.22 to 157.74) | | -2.33 (-2.75 to -1.91) | |
| India | 1188755 (835406 to 1609591) | | 517.91 (363.9 to 701.37) | | 563264 (418638 to 741575) | 246.35 (183.03 to 324.63) | | -2.38 (-2.52 to -2.24) | |
| Indonesia | 240001 (130017 to 377388) | | 530.71 (284.92 to 838.66) | | 150582 (101958 to 207268) | 339.19 (228.74 to 468.22) | | -1.4 (-1.47 to -1.33) | |
| Iran (Islamic Republic of) | 150403 (109602 to 200130) | | 836.37 (608.38 to 1115.98) | | 44165 (27973 to 59570) | 318.32 (198.98 to 433.85) | | -2.36 (-2.83 to -1.88) | |
| Iraq | 39169 (26309 to 55363) | | 668.41 (449.61 to 943.99) | | 35475 (25565 to 47789) | 400.6 (287.93 to 540.72) | | -1.27 (-1.49 to -1.04) | |
| Ireland | 2030 (1821 to 2263) | | 323.37 (289.88 to 360.69) | | 866 (729 to 1006) | 135.07 (113.33 to 157.63) | | -2.07 (-2.35 to -1.78) | |
| Israel | 4889 (4447 to 5360) | | 473.56 (430.62 to 519.33) | | 3114 (2590 to 3722) | 171.82 (142.87 to 205.33) | | -2.63 (-2.85 to -2.41) | |
| Italy | 26769 (25544 to 28126) | | 471.55 (449.62 to 495.85) | | 10210 (8722 to 11730) | 216.38 (183.39 to 250.07) | | -2.73 (-2.88 to -2.58) | |
| Jamaica | 3350 (2801 to 3940) | | 597.4 (499.04 to 703.17) | | 1189 (926 to 1511) | 323.06 (250.24 to 413.02) | | -1.83 (-2.02 to -1.65) | |
| Japan | 44309 (42630 to 45891) | | 310.31 (298.31 to 321.62) | | 17403 (15908 to 18923) | 177.19 (161.32 to 193.26) | | -1.66 (-1.82 to -1.5) | |
| Jordan | 5994 (4869 to 7418) | | 524.67 (426.13 to 649.49) | | 7562 (5651 to 9992) | 324.78 (241.41 to 431.48) | | -1.8 (-2.01 to -1.58) | |
| Kazakhstan | 25635 (22513 to 28865) | | 711.04 (624.73 to 800.35) | | 9423 (7583 to 11667) | 249.2 (200.63 to 308.38) | | -2.84 (-3.14 to -2.54) | |
| Kenya | 48128 (35052 to 63502) | | 586.27 (427.19 to 773.25) | | 49538 (36048 to 67372) | 411.86 (298.72 to 561.52) | | -0.27 (-0.63 to 0.1) | |
| Kiribati | 74 (46 to 106) | | 333.11 (206.15 to 482.61) | | 73 (33 to 139) | 255.96 (115.87 to 487.25) | | -0.95 (-1.14 to -0.76) | |
| Kuwait | 1672 (1436 to 1975) | | 425.92 (366.18 to 502.96) | | 959 (777 to 1172) | 170.13 (137.38 to 208.75) | | -2.04 (-2.44 to -1.64) | |
| Kyrgyzstan | 6646 (5321 to 8263) | | 549.2 (440.48 to 681.79) | | 4312 (3526 to 5253) | 271.21 (221.69 to 330.4) | | -1.88 (-2.23 to -1.52) | |
| Lao People's Democratic Republic | 10144 (3804 to 18042) | | 759.7 (290.03 to 1344.1) | | 6712 (4401 to 9830) | 422.54 (277.63 to 617.76) | | -1.9 (-1.99 to -1.81) | |
| Latvia | 1967 (1754 to 2207) | | 496.67 (443.04 to 557.33) | | 334 (262 to 417) | 172.06 (134.51 to 215.61) | | -2.35 (-2.69 to -2) | |
| Lebanon | 3070 (2204 to 4154) | | 416.94 (299.72 to 563.78) | | 2189 (1309 to 3427) | 259.52 (153.43 to 409.63) | | -1.59 (-1.79 to -1.38) | |
| Lesotho | 1035 (735 to 1432) | | 216.69 (153.85 to 299.93) | | 1276 (862 to 1828) | 305.58 (206.18 to 438.18) | | 1.7 (1.44 to 1.95) | |
| Liberia | 6554 (3977 to 8992) | | 745.24 (454.22 to 1022.34) | | 5240 (3134 to 7520) | 350.36 (209.66 to 502.65) | | -2.39 (-3.02 to -1.77) | |
| Libya | 7771 (5830 to 10130) | | 628.25 (471.6 to 818.75) | | 5388 (3712 to 7527) | 591.76 (403.06 to 833.67) | | 0.16 (-0.01 to 0.33) | |
| Lithuania | 2587 (2325 to 2880) | | 456.02 (409.78 to 507.52) | | 630 (520 to 761) | 229.35 (188.83 to 277.99) | | -2.03 (-2.41 to -1.64) | |
| Luxembourg | 206 (181 to 234) | | 457.4 (401.44 to 518.76) | | 125 (102 to 154) | 185.81 (151.67 to 229.86) | | -3.46 (-4.02 to -2.9) | |
| Madagascar | 35223 (26400 to 45088) | | 860.31 (642.17 to 1103.42) | | 41901 (27587 to 61516) | 520.18 (342.32 to 764.15) | | -1.27 (-1.45 to -1.09) | |
| Malawi | 61147 (46279 to 77765) | | 1710.35 (1290.58 to 2181.06) | | 49535 (23733 to 88759) | 920.72 (440.41 to 1650.57) | | -1.72 (-1.91 to -1.54) | |
| Malaysia | 15971 (10174 to 22883) | | 348.66 (222.25 to 499.3) | | 9565 (6329 to 13079) | 189.72 (125.53 to 259.49) | | -1.66 (-2.09 to -1.22) | |
| Maldives | 372 (181 to 619) | | 481.15 (237.97 to 795.65) | | 166 (115 to 230) | 251.69 (173.82 to 350.13) | | -1.84 (-1.99 to -1.68) | |
| Mali | 27762 (19094 to 37519) | 841.45 (576.68 to 1139.83) | | 39448 (22403 to 66145) | | | 451.99 (257.76 to 756.22) | | -1.73 (-1.88 to -1.57) |
| Malta | 195 (145 to 228) | 338.87 (250.8 to 395.58) | | 126 (100 to 155) | | | 290.54 (230.77 to 355.17) | | -0.64 (-1.22 to -0.04) |
| Marshall Islands | 25 (16 to 36) | 161.59 (104.31 to 235.95) | | 23 (14 to 34) | | | 196.7 (119.16 to 299.23) | | 0.48 (0.1 to 0.87) |
| Mauritania | 3137 (2105 to 4745) | 447.13 (299.96 to 674.5) | | 3824 (2361 to 5568) | | | 296.89 (183.29 to 432.23) | | -1.45 (-1.73 to -1.17) |
| Mauritius | 499 (445 to 562) | 235.58 (210.1 to 265.43) | | 168 (138 to 198) | | | 128.67 (105.92 to 151.58) | | -2.08 (-2.52 to -1.64) |
| Mexico | 185892 (167717 to 209428) | 815.49 (736.14 to 918.22) | | 75757 (59587 to 95994) | | | 365.29 (285.36 to 465.65) | | -2.13 (-2.41 to -1.85) |
| Micronesia (Federated States of) | 81 (53 to 116) | 258 (168.61 to 369.99) | | 32 (20 to 49) | | | 162.53 (102.11 to 250.7) | | -1.36 (-1.51 to -1.2) |
| Monaco | 12 (8 to 16) | 509.03 (352.08 to 704.32) | | 16 (12 to 20) | | | 481.21 (370.12 to 605.59) | | -2.03 (-2.78 to -1.27) |
| Mongolia | 7430 (5202 to 10035) | 1145.68 (803.34 to 1547.25) | | 2905 (2126 to 3804) | | | 372.52 (272.39 to 488.27) | | -3.47 (-3.82 to -3.12) |
| Montenegro | 592 (463 to 735) | 552.06 (430.63 to 687.38) | | 122 (65 to 188) | | | 166.69 (88.65 to 255.77) | | -3.4 (-3.81 to -2.99) |
| Morocco | 24347 (15949 to 34014) | 356.61 (233.79 to 497.91) | | 12243 (8111 to 18214) | | | 187.78 (124.36 to 279.81) | | -1.67 (-1.89 to -1.45) |
| Mozambique | 61127 (41946 to 83013) | 1314.22 (900.71 to 1788.4) | | 72128 (39297 to 128650) | | | 712.56 (388.42 to 1270.09) | | -1.6 (-1.8 to -1.4) |
| Myanmar | 88118 (33566 to 152289) | 883.82 (335.51 to 1528.71) | | 52956 (34039 to 78863) | | | 509.64 (327.08 to 759.91) | | -1.98 (-2.12 to -1.84) |
| Namibia | 1487 (998 to 2085) | 346.38 (232.77 to 485.24) | | 2386 (1578 to 3585) | | | 432.18 (285.72 to 649.71) | | 1.38 (0.99 to 1.78) |
| Nauru | 10 (7 to 13) | 321.31 (230.25 to 433.54) | | 9 (6 to 12) | | | 318.28 (215.56 to 449.4) | | -0.09 (-0.46 to 0.28) |
| Nepal | 40280 (21803 to 61448) | 651.91 (354.55 to 992.31) | | 18614 (13188 to 26123) | | | 303.25 (214.83 to 425.68) | | -2.19 (-2.35 to -2.03) |
| Netherlands | 6254 (5714 to 6775) | 342.81 (313.2 to 371.34) | | 2976 (2548 to 3460) | | | 170.34 (145.57 to 198.37) | | -2.5 (-2.69 to -2.31) |
| New Zealand | 1967 (1754 to 2205) | 366.59 (327.12 to 410.74) | | 1117 (973 to 1293) | | | 174.32 (151.67 to 202.11) | | -2.04 (-2.27 to -1.8) |
| Nicaragua | 11331 (8696 to 14576) | 877.92 (673.98 to 1129.19) | | 3732 (2693 to 5222) | | | 282.03 (202.96 to 395.93) | | -2.99 (-3.19 to -2.78) |
| Niger | 25979 (17804 to 35122) | 811.61 (554.93 to 1098.18) | | 34628 (19698 to 54040) | | | 359.91 (205.35 to 560.68) | | -2.56 (-2.8 to -2.33) |
| Nigeria | 230446 (177761 to 292368) | 771.06 (593.69 to 978.41) | | 446154 (241686 to 649061) | | | 621.34 (337.23 to 903.1) | | -0.42 (-0.56 to -0.29) |
| Niue | 1 (1 to 2) | 265.1 (180.68 to 381.87) | | 3 (2 to 4) | | | 1218.7 (917.83 to 1621.21) | | 1.63 (0.47 to 2.8) |
| North Macedonia | 2760 (2178 to 3400) | 797.78 (628.74 to 983.66) | | 573 (396 to 787) | | | 268.51 (184.16 to 371.25) | | -2.73 (-3.14 to -2.31) |
| Northern Mariana Islands | 10 (7 to 14) | 117.47 (81.6 to 166.3) | | 7 (5 to 10) | | | 98.47 (69 to 136.52) | | -0.06 (-0.26 to 0.14) |
| Norway | 1973 (1846 to 2105) | 369.94 (346.19 to 394.74) | | 793 (691 to 919) | | | 133.85 (116.24 to 155.65) | | -2.67 (-2.91 to -2.43) |
| Oman | 1686 (1219 to 2299) | 275.51 (199.17 to 375.62) | | 1094 (812 to 1387) | | | 126.19 (93.62 to 160.18) | | -1.71 (-2.08 to -1.35) |
| Pakistan | 257326 (173410 to 353416) | 730.04 (492.35 to 1002.14) | | 432322 (325550 to 566661) | | | 737.49 (555.27 to 966.84) | | 0.6 (0.4 to 0.81) |
| Palau | 5 (4 to 7) | 178.11 (122.9 to 248.53) | | 2 (2 to 3) | | | 125.48 (93.19 to 171.67) | | -0.92 (-1.08 to -0.76) |
| Palestine | 4190 (2761 to 5863) | 587.95 (390.43 to 818.63) | | 4301 (3224 to 5624) | | | 342.77 (256.46 to 449.03) | | -1.15 (-1.44 to -0.86) |
| Panama | 4019 (3390 to 4722) | 710.85 (599.36 to 835.37) | | 3481 (2811 to 4299) | | | 453.43 (365.06 to 562) | | -1.53 (-1.69 to -1.38) |
| Papua New Guinea | 3938 (2068 to 6390) | 322.54 (169.44 to 522.76) | | 8808 (5326 to 14290) | | | 309.91 (187.65 to 502.65) | | -0.09 (-0.35 to 0.18) |
| Paraguay | 6507 (5054 to 8331) | 547.63 (425.58 to 700.51) | | 5082 (3572 to 7217) | | | 384.86 (269.48 to 548.82) | | -0.61 (-0.97 to -0.25) |
| Peru | 54064 (42567 to 71991) | 948.45 (746.96 to 1262.4) | | 28409 (18790 to 40015) | | | 436.63 (288.62 to 615.2) | | -2.13 (-2.28 to -1.98) |
| Philippines | 116658 (78010 to 164861) | 662.24 (444.46 to 934.02) | | 94339 (72578 to 118377) | | | 417.3 (320.59 to 524.46) | | -0.89 (-1.08 to -0.69) |
| Poland | 33977 (31351 to 36095) | 545.47 (502.36 to 580.44) | | 6690 (5702 to 7758) | | | 175.29 (149 to 203.72) | | -3.47 (-3.64 to -3.31) |
| Portugal | 7680 (6954 to 8407) | 604.3 (546.32 to 662.76) | | 2022 (1728 to 2377) | | | 234.9 (200.56 to 276.34) | | -3.52 (-3.92 to -3.12) |
| Puerto Rico | 2177 (1943 to 2434) | 338.03 (301.66 to 378.03) | | 302 (250 to 364) | | | 126.49 (104.15 to 154.01) | | -2.17 (-2.46 to -1.88) |
| Qatar | 205 (151 to 280) | 227.88 (168.65 to 310.36) | | 375 (274 to 513) | | | 107.18 (78.36 to 146.3) | | -2.2 (-2.35 to -2.04) |
| Republic of Korea | 36780 (27945 to 45984) | 496.71 (376.28 to 621.93) | | 6230 (4188 to 8088) | | | 168.73 (111.58 to 222.16) | | -3.62 (-3.9 to -3.35) |
| Republic of Moldova | 9018 (7795 to 10495) | 1059.52 (915.63 to 1233.18) | | 1156 (906 to 1472) | | | 350.81 (273.02 to 450.05) | | -3.05 (-3.49 to -2.6) |
| Romania | 27709 (24355 to 31029) | 789.78 (693.97 to 884.77) | | 4717 (3995 to 5532) | | | 245.73 (207.6 to 288.82) | | -3.64 (-3.93 to -3.36) |
| Russian Federation | 167617 (161675 to 174098) | 711.13 (685.53 to 739.09) | | 38396 (34268 to 42448) | | | 228.21 (202.85 to 253.5) | | -4.03 (-4.37 to -3.69) |
| Rwanda | 31285 (23082 to 40548) | 1227.09 (905.86 to 1590.33) | | 21206 (14022 to 30937) | | | 621.37 (411.08 to 906.27) | | -2.38 (-2.65 to -2.12) |
| Saint Kitts and Nevis | 46 (39 to 54) | 487.64 (410.78 to 572.82) | | 23 (18 to 29) | | | 361.78 (281.92 to 459.2) | | -0.56 (-0.76 to -0.35) |
| Saint Lucia | 163 (132 to 197) | 467.27 (377.63 to 563.93) | | 65 (51 to 84) | | | 341.09 (262.84 to 444.73) | | -0.98 (-1.21 to -0.76) |
| Saint Vincent and the Grenadines | 172 (137 to 217) | 637.43 (507.04 to 809.98) | | 71 (58 to 87) | | | 431.49 (346.26 to 534.26) | | -1.14 (-1.5 to -0.78) |
| Samoa | 170 (108 to 240) | 347.65 (222.42 to 492.82) | | 134 (76 to 227) | | | 239.06 (135.92 to 404.5) | | -1.18 (-1.31 to -1.06) |
| San Marino | 17 (12 to 22) | 676.34 (490.33 to 907.37) | | 8 (5 to 10) | | | 287.86 (197.75 to 399.93) | | -2.34 (-2.47 to -2.21) |
| Sao Tome and Principe | 223 (156 to 305) | 557.22 (389.69 to 764.71) | | 94 (49 to 156) | | | 186.88 (96.72 to 309.51) | | -3.17 (-3.76 to -2.58) |
| Saudi Arabia | 17751 (12600 to 24539) | 378.99 (269.08 to 523.69) | | 4996 (3363 to 7355) | | | 98.76 (66.37 to 145.48) | | -4.43 (-4.52 to -4.35) |
| Senegal | 16598 (12558 to 21582) | 595.18 (449.5 to 775.95) | | 14528 (8916 to 22873) | | | 329.66 (202.57 to 518.66) | | -1.58 (-1.83 to -1.32) |
| Serbia | 9066 (6552 to 11903) | 647.05 (466.3 to 851.34) | | 1287 (962 to 1706) | | | 161.75 (120.35 to 215.24) | | -5.07 (-5.55 to -4.58) |
| Seychelles | 47 (35 to 61) | 292.7 (218.09 to 385.35) | | 28 (21 to 38) | | | 182.14 (134.47 to 241.69) | | -0.88 (-1.37 to -0.38) |
| Sierra Leone | 10876 (7467 to 14573) | 747.39 (513.45 to 1001.06) | | 11466 (7571 to 16315) | | | 446.42 (295.19 to 634.64) | | -1.56 (-1.7 to -1.41) |
| Singapore | 1556 (1380 to 1754) | 370.55 (328.62 to 417.97) | | 800 (668 to 956) | | | 138.47 (115.46 to 165.73) | | -2.32 (-2.71 to -1.93) |
| Slovakia | 3289 (2830 to 3842) | 385.02 (331.17 to 449.95) | | 1357 (1022 to 1773) | | | 239.91 (180.59 to 313.51) | | -1.41 (-1.53 to -1.29) |
| Slovenia | 751 (666 to 846) | 284.19 (251.57 to 320.41) | | 218 (178 to 263) | | | 105.8 (86.34 to 128.51) | | -2.71 (-3.02 to -2.39) |
| Solomon Islands | 240 (143 to 362) | 216.77 (128.67 to 326.85) | | 362 (210 to 571) | | | 199.56 (116.04 to 314.69) | | -0.2 (-0.54 to 0.14) |
| Somalia | 23400 (13666 to 34776) | 797.47 (465.65 to 1184.2) | | 34728 (18672 to 55242) | | | 450.2 (242.02 to 715.54) | | -1.31 (-1.72 to -0.91) |
| South Africa | 28839 (20590 to 37914) | 306.2 (219.15 to 402.06) | | 24455 (18957 to 30645) | | | 241.63 (186.91 to 303.23) | | -0.59 (-0.9 to -0.29) |
| South Sudan | 20428 (13291 to 28576) | 1047.17 (681.93 to 1463.53) | | 26835 (17390 to 37693) | | | 898.38 (583.24 to 1261.23) | | 0.08 (-0.43 to 0.59) |
| Spain | 18885 (17380 to 20450) | 406.53 (373.69 to 440.65) | | 8912 (7929 to 10121) | | | 218.59 (193.63 to 249.36) | | -2.19 (-2.3 to -2.08) |
| Sri Lanka | 16185 (12550 to 21047) | 437.42 (339.29 to 568.11) | | 5064 (3555 to 7227) | | | 152.14 (106.29 to 217.86) | | -3.82 (-4.27 to -3.37) |
| Sudan | 67134 (30751 to 110906) | 1026.71 (475.43 to 1691.03) | | 64782 (43442 to 92488) | | | 577.59 (387.19 to 825.22) | | -1.52 (-1.68 to -1.36) |
| Suriname | 550 (379 to 689) | 619.72 (426.87 to 776.26) | | 467 (344 to 607) | | | 501.17 (367.59 to 653.36) | | -0.51 (-0.74 to -0.29) |
| Sweden | 3910 (3524 to 4343) | 370.53 (334.12 to 411.45) | | 1826 (1552 to 2197) | | | 152.33 (129.33 to 183.32) | | -2.47 (-2.64 to -2.3) |
| Switzerland | 2620 (2377 to 2891) | 335.14 (304.08 to 369.82) | | 1714 (1443 to 2059) | | | 191.87 (161.17 to 230.93) | | -2.06 (-2.38 to -1.74) |
| Syrian Arab Republic | 25533 (16162 to 35838) | 611.74 (387.96 to 857.95) | | 5538 (3739 to 7865) | | | 260.11 (173.77 to 371.7) | | -2.48 (-2.82 to -2.14) |
| Taiwan (Province of China) | 11055 (10182 to 12054) | 316.64 (291.24 to 345.48) | | 3315 (2805 to 3893) | | | 172.05 (145.07 to 202.78) | | -1.63 (-1.84 to -1.42) |
| Tajikistan | 17664 (12454 to 24405) | 1010.4 (717.85 to 1388.81) | | 17974 (12707 to 25356) | | | 702.07 (497.46 to 988.5) | | -1.34 (-1.58 to -1.11) |
| Thailand | 59482 (43056 to 77847) | 545.02 (394.07 to 713.85) | | 16471 (12813 to 20486) | | | 269 (208.43 to 335.57) | | -2.58 (-2.85 to -2.31) |
| Timor-Leste | 1828 (737 to 3136) | 710.55 (297.11 to 1206.9) | | 1393 (887 to 2107) | | | 391.75 (250.21 to 591.88) | | -2.13 (-2.42 to -1.84) |
| Togo | 5904 (4391 to 7820) | 449.59 (334 to 596.06) | | 6998 (3810 to 11127) | | | 304.25 (165.63 to 483.73) | | -1.09 (-1.21 to -0.96) |
| Tokelau | 1 (1 to 2) | 261.31 (165.36 to 390.48) | | 4 (2 to 6) | | | 1774.23 (952.76 to 2866.89) | | 1.04 (-0.94 to 3.06) |
| Tonga | 82 (56 to 114) | 281.74 (190.72 to 389.18) | | 72 (50 to 111) | | | 261.3 (179.08 to 399.27) | | -0.42 (-0.76 to -0.07) |
| Trinidad and Tobago | 1525 (1284 to 1774) | 541.88 (455.32 to 631.42) | | 590 (462 to 763) | | | 336.38 (260.59 to 439.66) | | -1.19 (-1.37 to -1.01) |
| Tunisia | 13004 (9758 to 16862) | 613.73 (460.29 to 796.03) | | 4462 (3162 to 6158) | | | 239.98 (169.62 to 332.55) | | -2.79 (-2.88 to -2.7) |
| Turkey | 132317 (92004 to 178306) | 966.71 (672.12 to 1302.71) | | 39088 (30012 to 50583) | | | 326.65 (250.03 to 424.14) | | -3.48 (-3.73 to -3.23) |
| Turkmenistan | 9349 (7781 to 10989) | 849.47 (709.35 to 996.25) | | 5173 (4183 to 6269) | | | 490.46 (396.64 to 594.28) | | -1.29 (-1.54 to -1.04) |
| Tuvalu | 9 (4 to 14) | 323.38 (168.28 to 518.55) | | 5 (3 to 6) | | | 178.39 (119.33 to 256.05) | | -1.7 (-1.84 to -1.57) |
| Uganda | 72113 (51405 to 102646) | 1081.23 (768.85 to 1536.54) | | 123283 (68513 to 200057) | | | 875.95 (489.04 to 1417.01) | | -0.45 (-0.65 to -0.24) |
| Ukraine | 61573 (51278 to 72561) | 805.6 (670.24 to 950.05) | | 13672 (10954 to 16654) | | | 363.43 (285.61 to 448.72) | | -2.46 (-2.65 to -2.26) |
| United Arab Emirates | 1415 (996 to 1970) | 318.9 (224.5 to 444.01) | | 1285 (867 to 1827) | | | 139.34 (93.58 to 198.96) | | -1.66 (-2.02 to -1.3) |
| United Kingdom | 26927 (25912 to 28020) | 360.16 (346.63 to 374.73) | | 13076 (11831 to 14154) | | | 170.74 (154.26 to 185.12) | | -1.8 (-2.11 to -1.49) |
| United Republic of Tanzania | 103270 (78992 to 128410) | 1140.95 (873.23 to 1420.45) | | 139507 (92362 to 200634) | | | 813.07 (539.17 to 1167.85) | | -0.48 (-0.74 to -0.23) |
| United States of America | 122927 (119806 to 126080) | 320.75 (312.63 to 328.95) | | 71421 (65277 to 78209) | | | 186.37 (170.11 to 204.35) | | -1.38 (-1.49 to -1.27) |
| United States Virgin Islands | 71 (51 to 92) | 333.32 (239.84 to 433.32) | | 11 (6 to 19) | | | 146.08 (82.32 to 239.41) | | -1.17 (-1.72 to -0.62) |
| Uruguay | 2470 (2199 to 2785) | 454.69 (404.78 to 512.59) | | 1003 (810 to 1254) | | | 230.31 (184.64 to 290.07) | | -2.36 (-2.71 to -2.02) |
| Uzbekistan | 40061 (32196 to 49245) | 640.07 (515.7 to 784.62) | | 40154 (31983 to 50067) | | | 556.58 (444.37 to 692.64) | | -0.23 (-0.34 to -0.12) |
| Vanuatu | 81 (50 to 122) | 163.18 (100.91 to 246.31) | | 125 (74 to 188) | | | 153.24 (90.91 to 228.94) | | 0.09 (-0.35 to 0.52) |
| Venezuela (Bolivarian Republic of) | 28832 (26545 to 31218) | 591.03 (544.29 to 639.75) | | 18134 (13347 to 23645) | | | 412.35 (302.28 to 539.24) | | -0.69 (-1.01 to -0.37) |
| Viet Nam | 82703 (58984 to 109876) | 447.4 (319.07 to 594.39) | | 41723 (27236 to 59703) | | | 249.72 (162.98 to 357.53) | | -1.74 (-1.83 to -1.64) |
| Yemen | 33735 (17028 to 55795) | 642 (326.05 to 1059.02) | | 38602 (25460 to 55428) | | | 412.95 (272.34 to 592.96) | | -1.25 (-1.39 to -1.1) |
| Zambia | 35513 (27055 to 44930) | 1244.68 (948.33 to 1575.48) | | 38873 (24616 to 59197) | | | 677.76 (429.21 to 1032.11) | | -1.71 (-1.87 to -1.55) |
| Zimbabwe | 9871 (6597 to 13169) | 289.92 (193.45 to 387.2) | | 20874 (14358 to 29021) | | | 481.55 (331.24 to 669.49) | | 2.98 (2.3 to 3.67) |

EAPC, estimated annual percentage change, SDl, Sociodemographic Index; Ul, uncertainty interval. EAPC is expressed as 95% CIs.

**Figure S1** Trends in the disease burden of urogenital congenital anomalies (UGCAs) in children under 9 years old from 1990 to 2021 across different age groups and SDI regions (A. Different age groups; B. Age-Standardized Rates (ASRs) across different SDI regions; C. Absolute numbers across different SDI regions).


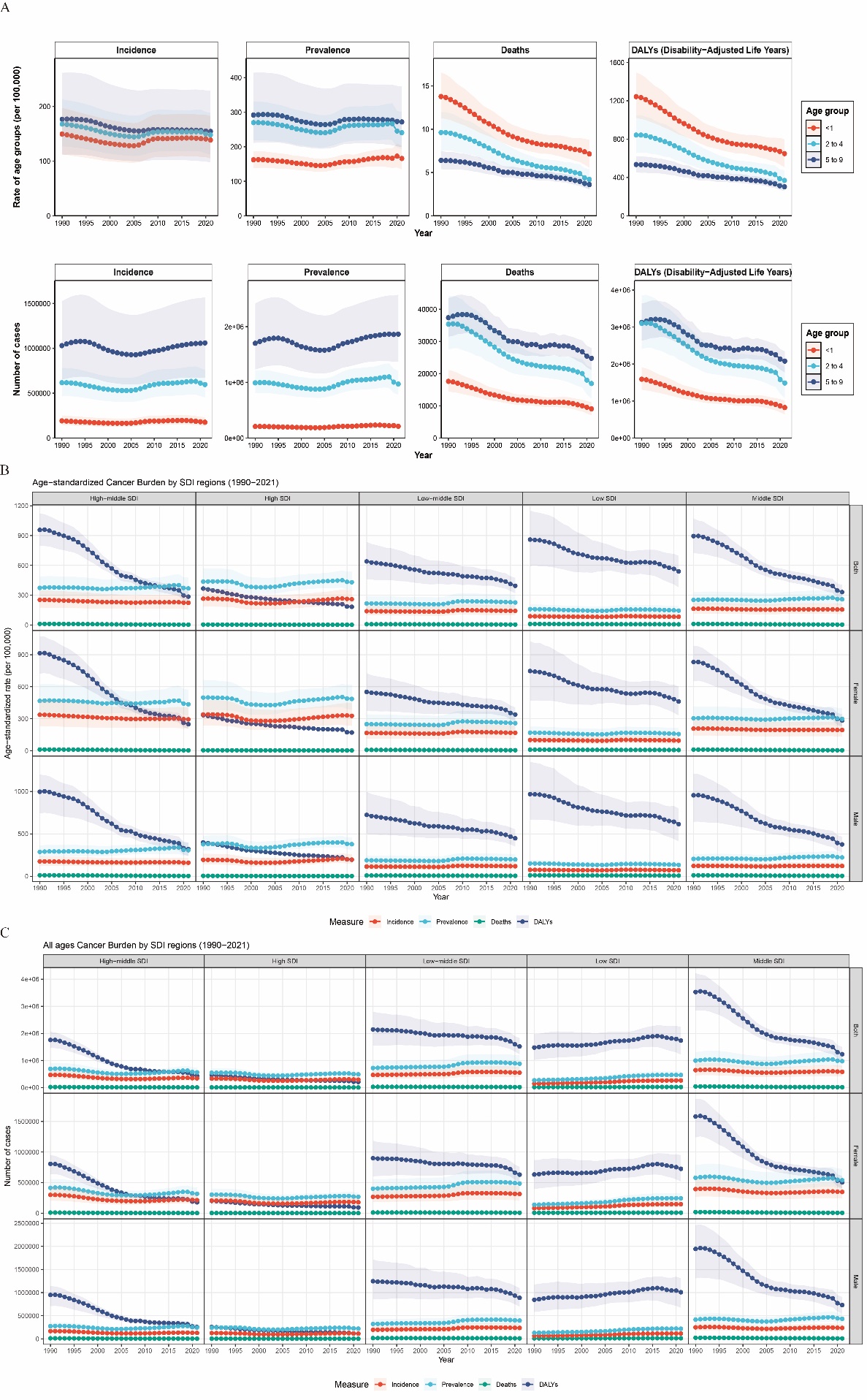


**Figure S2** Holt-Winters model forecast of the disease burden of urogenital congenital anomalies (UGCAs) in children under 9 years old globally by 2035(A. Incidence; B. Prevalence; C. Deaths; D. DALYs).

**
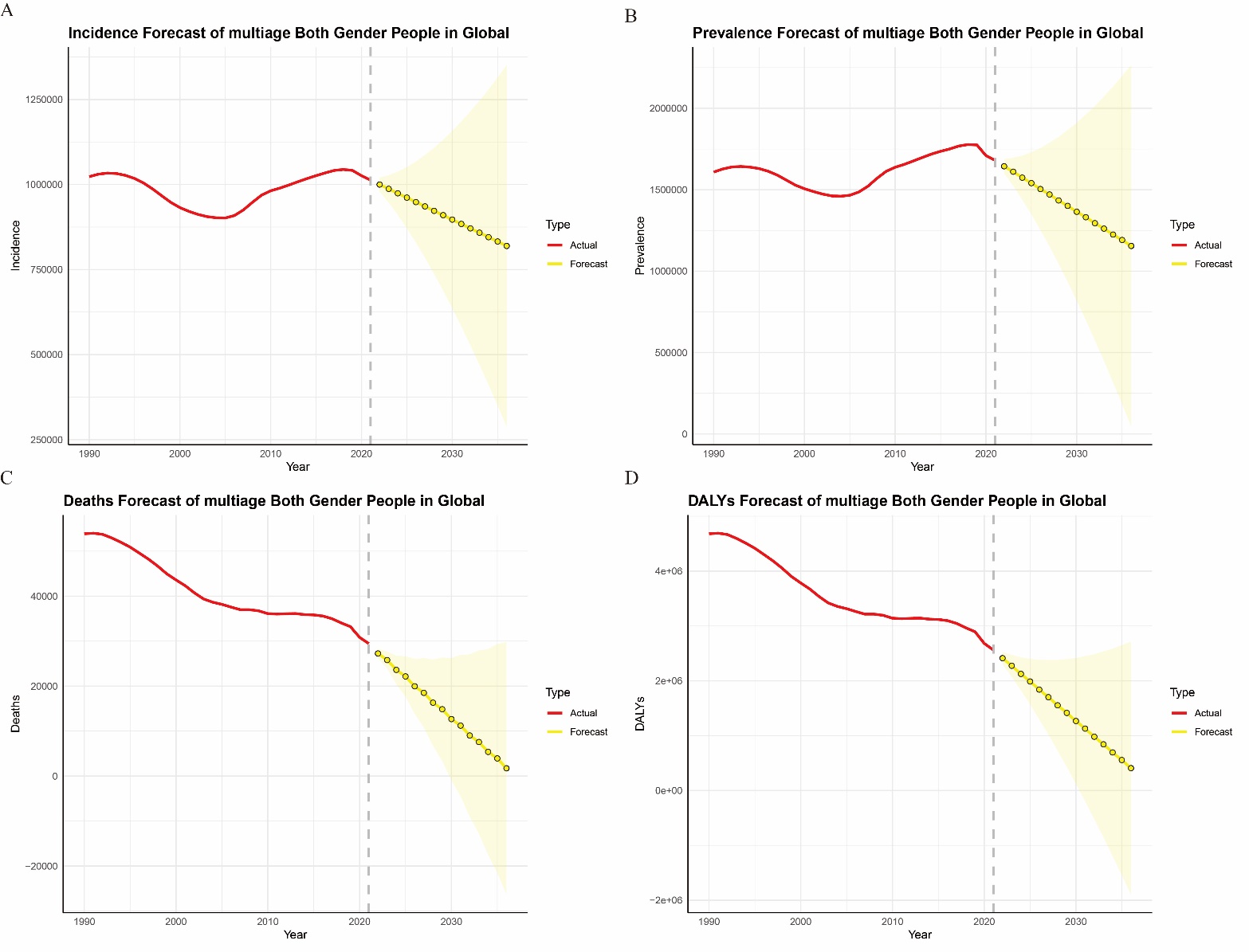
**
